# Effects of Aging on Muscle Development Based on Transcriptome Sequencing in Dahen Broilers

**DOI:** 10.3390/ani16172743

**Published:** 2026-09-02

**Authors:** Jiang-Xian Wang, Mo-Han Qiu, Zeng-Rong Zhang, Shi-Liang Zhu, Xia Xiong, Chen-Ming Hu, Li Yang, Han Peng, Xiao-Yan Song, Jia-Lei Chen, Bo Xia, Long-Huan Du, Jia-Yi Zhao, Yue-Ying Zhang, Jia-Lu Li, Chun-Lin Yu, Chao-Wu Yang

**Affiliations:** 1 Animal Genetic Breeding and Reproduction Key Laboratory of Sichuan Province, Sichuan Animal Science Academy, Chengdu 610066, China; wangjiangxian@scsaas.cn (J.-X.W.); mohan.qiu@163.com (M.-H.Q.); zhangzengrong2004@163.com (Z.-R.Z.); zhushiliang1994@163.com (S.-L.Z.); xiongxia20120904@163.com (X.X.); huchenming@126.com (C.-M.H.); yangli_sasa@163.com (L.Y.); penghan0706@163.com (H.P.); babalasxy@163.com (X.-Y.S.); qiaoqiaowo110@163.com (J.-L.C.); allanbobo777@163.com (B.X.); longhuan_du@scsaas.cn (L.-H.D.); 18383247615@163.com (J.-Y.Z.); z226089@126.com (Y.-Y.Z.); 15528073191@163.com (J.-L.L.); 2 Sichuan Dahen Poultry Breeding Co., Ltd., Chengdu 610066, China

**Keywords:** Dahen chicken, muscle development, meat quality, muscle fiber characteristics, transcriptome analysis

## Abstract

Skeletal muscle growth and development represent a pivotal biological process that determines both the yield and quality of poultry meat. As chickens age, there is an observable shift in their muscle structure, function and metabolic properties. However, the molecular mechanisms by which age regulates chicken muscle development and meat quality formation are not yet fully understood. The objective of this study was to elucidate the fundamental molecular mechanisms through which the aging process influences the development of chicken muscle and the quality of meat. A total of 20 female Dahen chickens, comprising 10 birds at 3 months of age and 10 at 72 months of age, were subjected to systematic measurements of meat quality indexes and muscle fiber characteristics. From each age group, 3 out of the 10 birds were randomly selected for breast muscle transcriptome analysis. The results demonstrated that aged chicken breast muscle exhibited reduced tenderness, augmented intramuscular fat content and water-holding capacity, while muscle fibers demonstrated significant hypertrophy and reduced density. A total of 819 differentially expressed genes (DEGs) were identified by RNA-seq, which were predominantly enriched in the biological processes of muscle system process, muscle organ development, and skeletal muscle cell differentiation, and were involved in the pathways of cytoskeleton in muscle cells, and extracellular matrix (ECM)–receptor interaction signaling, among others. The expression of key genes, such as *COL1A2*, *THBS2*, *FN1,* and *MYBPC1*, was found to be significantly altered, a finding that was verified by qPCR in agreement with the sequencing results. The study demonstrated that age impacts meat quality traits by inducing muscle fiber hypertrophy, ECM remodeling and metabolic reprogramming. The related genes provide a valuable reference point for understanding the genetic regulation of avian muscle development and meat quality.

## Simple Summary

This study compared breast muscle from 3- and 72-month-old chickens to reveal age-related effects. Aged muscle exhibited fiber hypertrophy, lower density, reduced tenderness, and increased fat and water-holding capacity. Transcriptomics identified 819 DEGs enriched in muscle development and ECM pathways, with key genes (*COL1A2*, *FN1*) validated. Aging modulates meat quality via myofiber remodeling and metabolic reprogramming, offering a genetic basis for poultry improvement.

## 1. Introduction

Skeletal muscles are the most abundant tissue in the bodies of most vertebrates and play a crucial role in numerous physiological processes essential for life. The primary function is movement and posture. They also plays a pivotal role as a major metabolic organ, responsible for a significant portion of energy consumption, glucose processing, and amino acid storage in the body [1]. Within the domain of agricultural production, particularly in the context of the poultry industry, skeletal muscle constitutes the primary economic product, namely meat. Consequently, the growth, development, and ultimate quality of muscle tissue are of paramount importance, which has resulted in extensive research on the genetic, nutritional, and environmental factors that control these characteristics [2,3]. The process of muscle development is an intricate biological process that commences during embryogenesis and continues post-hatch. The process entails the proliferation of myoblasts, their subsequent withdrawal from the cell cycle, differentiation, and fusion to form multinucleated myotubes, which then mature into functional muscle fibers [4]. The number and size of these muscle fibers are the primary determinants of muscle mass. This entire process is regulated by a complex network of signaling pathways [4]. The final characteristics of the meat, including its texture, flavor, and nutritional value, are influenced by a multitude of factors throughout the chickens’ lives. These factors include genetic background [5], maternal and post-hatch nutrition [6], and environmental conditions like incubation temperature [7]. However, poultry meat quality is increasingly compromised by muscle fiber structural defects and myopathies, such as white striping (WS), wooden breast (WB), spaghetti meat (SM), deep pectoral myopathy (DPM), and pale, soft, exudative (PSE) meat [8,9,10,11]. They are highly prevalent, frequently found together, and represent a considerable financial cost to the poultry sector.

Beyond genetic and age-related factors, nutrition plays a pivotal role in modulating skeletal muscle development and meat quality in poultry. Dietary composition (particularly protein and amino acid intake) affects muscle growth, postmortem metabolism, and the occurrence of myodegenerative defects [12,13]. Furthermore, nutritional interventions, including the regulation of protein intake, fatty acid balance, and antioxidant levels, are crucial for maintaining muscle integrity and oxidative stability during aging [14,15].

Among the numerous factors influencing muscle physiology, aging is a universal and inexorable process that leads to a progressive decline in tissue structure and function [16]. In skeletal muscle, the hallmark of aging is sarcopenia, a syndrome characterized by the accelerated and generalized loss of muscle mass, strength, and function [17,18]. The risk of sarcopenia can be influenced by various factors, including age and pre-existing conditions [19,20]. It is evident that regular physical activity can mitigate some of the aforementioned effects and promote healthy muscle aging [16,21]. However, the underlying biological deterioration persists.

In the avian model, particularly the chicken, the study of aging presents a unique paradigm. Modern commercial broilers have been the subject of intensive selection for rapid growth, and are typically slaughtered at a very young age, often between 35 and 49 days, in order to maximize feed efficiency and muscle yield [22,23]. Conversely, a significant proportion of local or dual-purpose breeds, in addition to laying hens approaching the conclusion of their productive cycle, are processed at considerably older ages [24]. The age at which poultry is slaughtered is a critical determinant of meat quality [25,26]. A previous study demonstrated that shear force was a significant predictor of meat tenderness, with the magnitude of this relationship increasing in a stepwise manner with advancing age [27]. Furthermore, other meat quality attributes, such as the intramuscular fat (IMF) content, which contributes to juiciness and flavor, also undergo age-dependent changes [28].

The development of the market has resulted in the evolution of high-throughput sequencing from a cutting-edge technology to a fundamental industry with applications across various fields. Transcriptome sequencing (RNA-seq) employs high-throughput technology to comprehensively analyze RNA, enabling precise quantification of gene expression, identification of differentially expressed genes, and variable splicing. This facilitates the elucidation of the molecular mechanisms underlying life activities [29,30]. In the domain of poultry science, RNA sequencing technology has been extensively utilized to analyze muscle biological processes [31,32]. Despite the extensive research that has been conducted on the early muscle development and meat quality of broilers, there remains a paucity of knowledge concerning the molecular mechanisms of skeletal muscle during extreme aging. By directly comparing young mature individuals with extremely aged individuals, the biological basis of muscle aging distinct from early development can be revealed.

## 2. Materials and Methods

### 2.1. Ethical Statement

All animal experimental procedures were approved by the Institutional Animal Care and Utilization Committee (IACUC) of Sichuan Animal Science Academy (permit number: 2025013).

### 2.2. Experimental Design and Sample Collection

This study aimed to investigate the impact of aging on muscle development and meat quality in Dahen broilers by comparing two biologically distinct age extremes, namely 3 months (young adulthood) and 72 months (extreme senescence), to identify the major molecular signatures of muscle senescence via integrated phenotyping and transcriptome analysis. The chickens used in this experiment were provided by Sichuan Dahen Poultry Breeding Co., Ltd. (Chengdu, China). The Dahen broiler is a Chinese nationally certified three-line hybrid meat breed, developed in Sichuan Province, with green shanks and variegated plumage. At 10 weeks of age, males average 2668 g and females 2269 g body weight, with a feed conversion ratio of approximately 2.3 kg of feed/kg BW during the 1–70-day period; the breed has been extensively distributed across 18 provinces [33]. A total of 200 females from the Dahen broilers were included in this study. The chickens were raised under identical conditions, had unrestricted access to water, and were fed different granular feeds according to their growth stages, with the same nutritional level of the feed. The complete feed ingredients and calculated nutritional values are presented in Table S1. The experiment was divided into two groups: the first group consisted of 3-month-old Dahen chickens, while the second group consisted of 72-month-old Dahen chickens, with 100 birds in each group. Ten chickens were randomly selected from each group for the subsequent experiments. Following a 12 h fast, all chickens were euthanized by electric shock and then had their cervical vertebrae severed to ensure death. Subsequently, the chickens were dissected. The left pectoral muscle tissue was utilized for the purpose of meat quality assessment. Three samples of the right pectoral muscle tissue were collected. One sample was preserved in a tissue preservation solution, while the remaining two were immediately frozen in liquid nitrogen and subsequently stored at −80 °C in a refrigerator.

### 2.3. Measurement

Subsequent to the slaughtering process, the left pectoral muscle was meticulously separated. The pH value, meat color, drip loss, cooking loss, and shear force were measured in accordance with the methods reported by Wang et al. [24,34,35]. The intramuscular fat (IMF) and inosine monophosphate (IMP) were determined by the method of Ding et al. [36,37,38]. The meat color of the pectoral muscle was measured using an Opto-Star colorimeter (Matthaus Company, Eckelsheim, Germany) within 45 min of slaughter. Measurements were taken on the inner side at three positions (upper, middle, lower), each with an area of 2 cm^2^, and the three values were averaged. The pH value of the pectoral muscle was measured after slaughter using a meat pH-Star meter (Matthaus Company, Eckelsheim, Germany). The measurement was repeated three times and averaged. Fresh pectoralis major muscle samples were trimmed of visible fat, tendons, and connective tissue. Each sample (approximately 30 g) was weighed, placed in a plastic self-sealing bag, and cooked in a water bath at 80 °C for 30 min. After cooking, the samples were removed from the bags, cooled to room temperature for 30 min, blotted dry with filter paper, and reweighed. Cooking loss was calculated as the percentage of weight loss relative to the initial weight. Subsequently, shear force was measured on the cooked samples using a meat tenderness tester (Guangzhou Runhu Instruments Co., Ltd., Guangzhou, China). The cooked muscle was cut into blocks of approximately 3 cm × 1 cm × 1 cm, and the measurement was repeated three times per sample, with the average value recorded as the final shear force. The weight of a section of the meat sample was measured by suspending it in a dry plastic bottle, which was then sealed and stored in a 4 °C refrigerator. Subsequent to a 24 h period, the weight of the muscle sample was measured and expressed as a percentage of the initial weight, thereby defining the drip loss. IMF was determined using the Soxhlet extraction method, and IMP was measured by high-performance liquid chromatography.

The left pectoral muscle was sampled by incising a piece measuring approximately 1 cm × 1 cm × 1 cm along the direction of the muscle fibers. The sample was then fixed in 4% paraformaldehyde solution for a period of 24 h. The fixed tissue was then dehydrated using an automatic dehydration machine (Wuhan Junjie Electronic Co., Ltd., Wuhan, China), after which it was embedded in paraffin. The sections were stained with hematoxylin–eosin (HE). The observations of the sections were conducted utilizing a digital three-microscope (Makedei Industrial Group Co., Ltd., Xiamen, China). The images were processed using Motic Images Advanced 3.2 software. For each sample, three images were collected, and the diameter, area, and density of the muscle fibers were measured and calculated using Motic Images Advanced 3.2 software. The muscle fiber area is defined as the actual area of the selected muscle fibers that has been calculated directly. The muscle fiber diameter is defined as the short diameter of the muscle fiber. The muscle fiber density refers to the number of muscle fibers present within an area at 400× magnification (400 times magnification covers an area of 53,660.72 µm^2^).

### 2.4. RNA-Seq

The TRIzol method (Invitrogen, Carlsbad, CA, USA) was utilized for the extraction of total RNA from six pectoral muscle tissue samples (three randomly selected from each of two age groups). The RNA quality was checked using the kaiaoK5500^®^ Spectrophotometer (Kaiao, Beijing, China). RNA integrity and concentration were assessed using the RNA Nano 6000 Assay Kit of the Bioanalyzer 2100 system (Agilent Technologies, Santa Clara, CA, USA). The preparation of RNA-seq transcriptome libraries was undertaken using a VAHTS Universal V6 RNA-seq Library Prep Kit for MGI^®^ (#NRM605) from Illumina (Vazyme Biotech Co., Ltd., Nanjing, China), with this process being conducted in accordance with the manufacturer’s recommendations. Paired-end libraries were sequenced on a DNBSEQ T7 sequencer. This experiment was completed by Wuhan Benagen Technology Co., Ltd. (Wuhan, China).

### 2.5. RNA-Seq Data Analysis

The raw data were first processed with FastQC (v0.11.9, default) (http://www.bioinformatics.babraham.ac.uk/projects/fastqc/ accessed on 3 July 2025) to filter out adapters and low-quality sequences. Then, the clean reads were mapped to the reference genome (Galgal 7.0) using Star (v2.7.9a, default) [39]. RSEM (v1.3.3, default) was used to calculate gene expression levels for each sample, expressed as fragments per kilobase of transcript per million fragments mapped (FPKM) [40]. The gene expression levels in 72-month-old samples were compared with those in 3-month-old samples in order to identify the differentially expressed genes (DEGs) by DESeq2 (v1.34.0, default) [41]. The DEGs were detected as previously described based on the parameters: |log2fold-change| > 1 with a significant false discovery rate-adjusted *p*-value (FDR) < 0.05 based on the three biological replicates. Gene Ontology (GO) and Kyoto Encyclopedia of Genes and Genomes (KEGG) enrichment analyses for the DEGs were performed using the clusterProfiler version 3.8 [42].

### 2.6. Real-Time Fluorescence Quantitative PCR

To verify the accuracy of the transcriptome data, 6 muscle tissue samples from Dahen chickens (3-month-old group and 72-month-old group with 3 samples, respectively) were subjected to quantitative real-time PCR (qPCR) analysis. qPCR analysis was conducted on the *PDGFD*, *MYBPC1*, *TPM2*, *MYOZ2*, *FHL1*, *COL1A2*, *THBS2*, *FN1*, *COL3A1*, and *COL1A1* genes. The specific primers for gene amplification are shown in Supplementary Table S2 and were designed using the NCBI database (https://www.ncbi.nlm.nih.gov/). Total RNA was reverse transcribed using a PrimeScript™ RT reagent kit (Takara Bio Inc., Beijing, China) in accordance with the manufacturer’s instructions. qPCR analysis was performed on the ABI Prism 7500 instrument (Applied Biosystems, Carlsbad, CA, USA) using SuperReal PreMix SYBR Green (TIANGEN BIOTECH (Beijing) Co., Ltd., Beijing, China). The relative gene expression levels were determined by utilizing the 2^−ΔΔCt^ method, with the *GAPDH* gene serving as the reference for normalization [43].

### 2.7. Statistical Analysis

Following the processing of the experimental data in Excel 2019, a *t*-test analysis was conducted using the software. The data were presented as the mean ± standard deviation. The data were plotted using GraphPad Prism 8.00 (GraphPad Software, Boston, MA, USA). A *p*-value of less than 0.05 was considered to indicate a statistically significant difference.

## 3. Results

### 3.1. Meat Quality

The meat quality from different growth stages of Dahen chickens is shown in Table 1. The results of the study demonstrated that the meat color, pH, shear force, and IMF of 3-month-old Dahen chickens were significantly lower than those of 72-month-old Dahen chickens (*p* < 0.01). In contrast, the drip loss, cooking loss, and IMP of 3-month-old Dahen chickens were significantly higher than those of 72-month-old Dahen chickens (*p* < 0.01).

### 3.2. Muscle Fiber Characteristics

**Figure 1:**
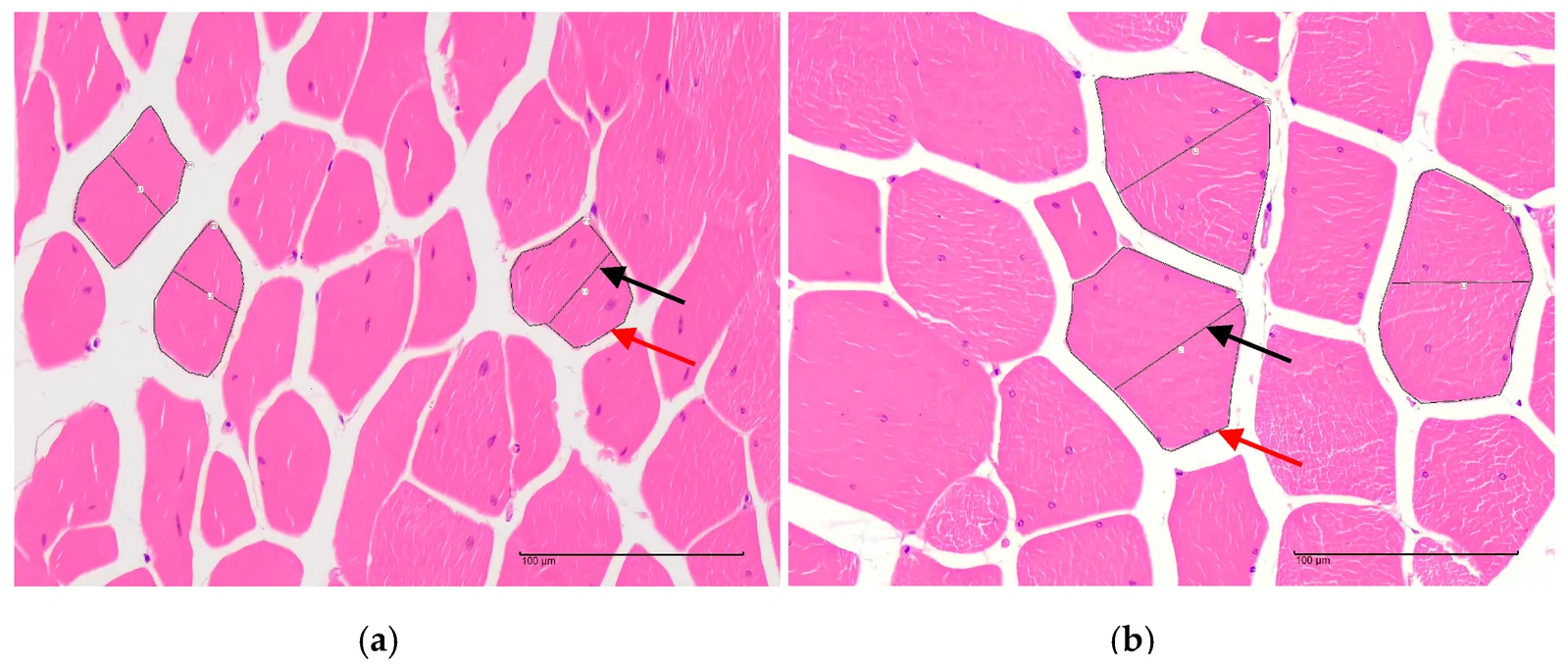
Schematic diagram of HE staining of pectoral muscle tissue observation (400×). (**a**) The pectoral muscle tissue of 3-month-old Dahen chickens; (**b**) The pectoral muscle tissue of 72-month-old Dahen chickens. The black arrow denotes the measurement of muscle fiber diameter, which is typically the measurement of the short diameter of the muscle fiber. The red arrow denotes the measurement of muscle fiber area.

The muscle fiber characteristics from different growth stages of Dahen chickens are shown in Table 2 and Figure 1. The results indicate that the muscle fiber diameter and area of 3-month-old Dahen chickens are significantly lower than those of 72-month-old Dahen chickens (*p* < 0.01), while the muscle fiber density of 3-month-old Dahen chickens is significantly higher than that of 72-month-old Dahen chickens (*p* < 0.01).

### 3.3. Sequencing Data Analysis

**Figure 2:**
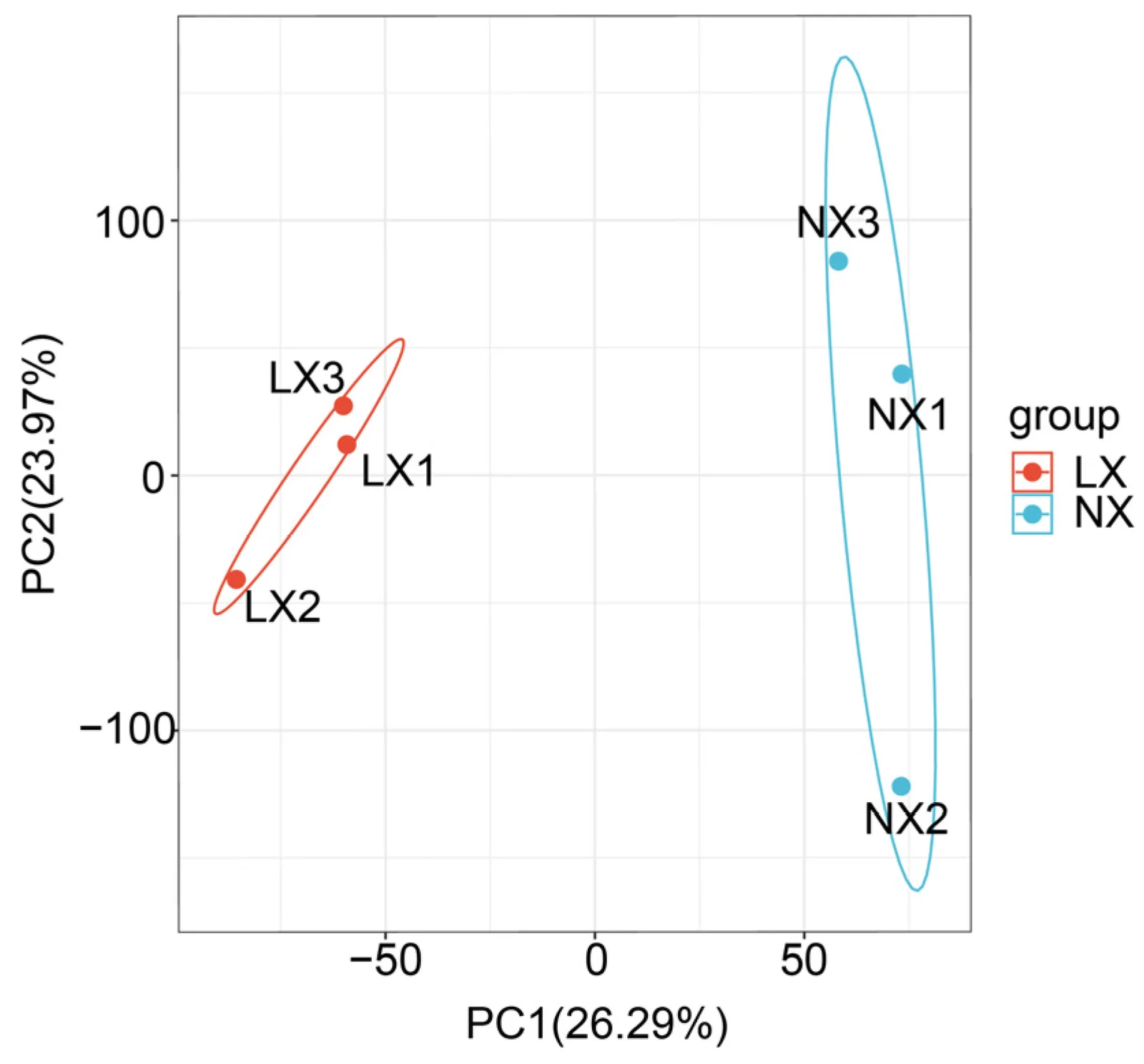
PCA of the transcriptome profiles for the 3-month-old group and the 72-month-old group. LX, LX1, LX2 and LX3 is the 72-month-old group, NX, NX1, NX2 and NX3 is 3-month-old group.

The evaluation of the transcriptome data is demonstrated in Table S3. Six cDNA libraries of 6 GB each were constructed from the total RNA of pectoral muscle tissue samples collected from three pectoral muscle tissues at 3 months old and three pectoral muscle tissues at 72 months old. The proportions of Q20 bases of six samples ranged from 99.01% to 99.24%. The proportions of Q30 bases of six samples ranged from 96.59% to 97.27%. Subsequent to the implementation of quality control measures, the data were then mapped to the chicken reference genome (Galgal 7.0). The GC content ranged from 48.47% to 49.91%, and the proportion of reads with a unique alignment position on the reference sequence for six samples ranged from 89.64% to 92.57%. Principal Component Analysis (PCA) of the transcriptome profiles showed a clear separation between the 3-month-old group and the 72-month-old group, indicating distinct gene expression patterns associated with age (Figure 2).

### 3.4. Differentially Expressed Genes (Degs) of Muscle Development

**Figure 3:**
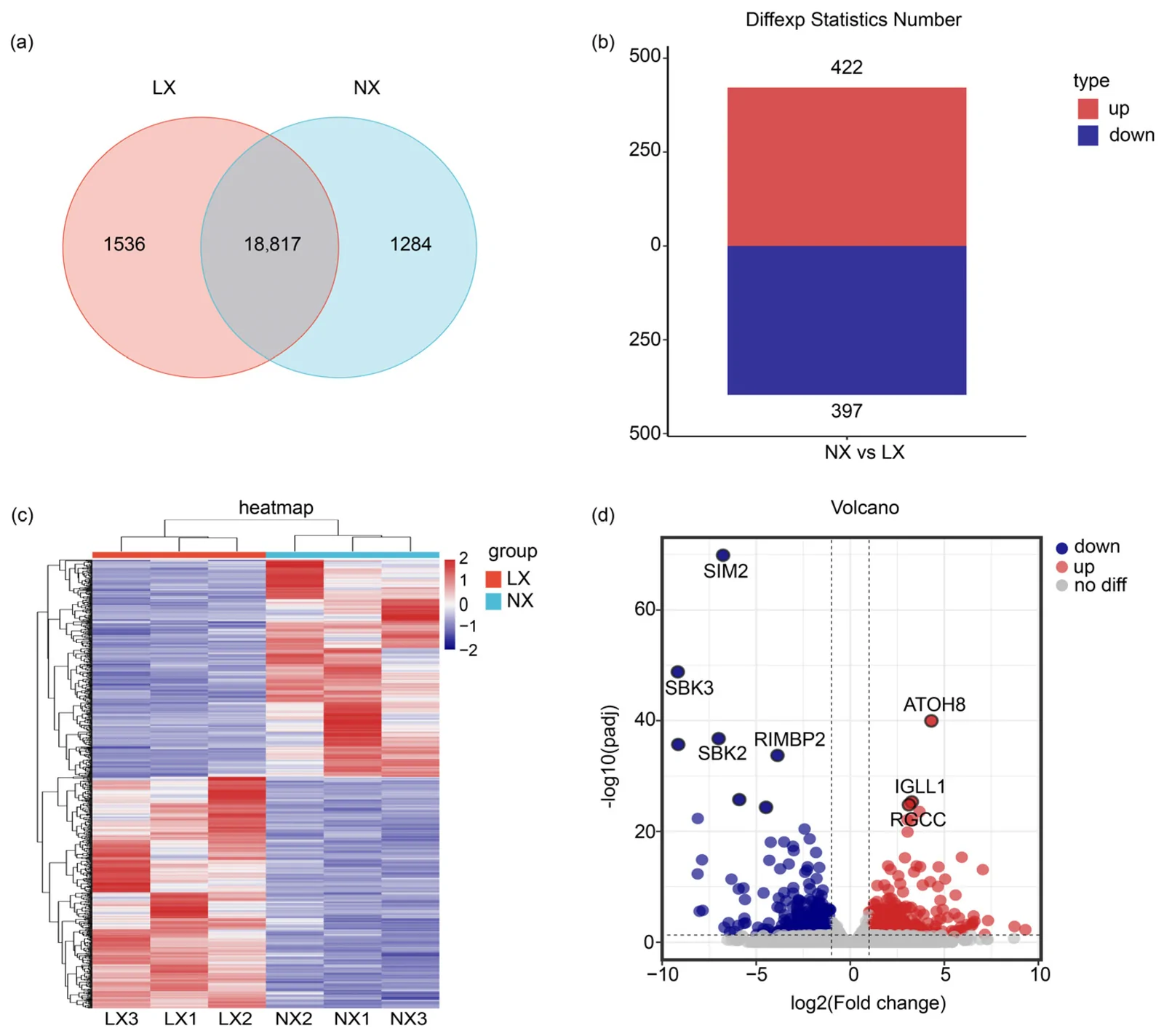
The analysis of DEGs in the transcriptomes of pectoral muscle of the 3-month-old and 72-month-old groups. (**a**) Venn diagram for genes at different stages of pectoral muscle development. (**b**) Number of DEGs in the 72-month-old group compared to the 3-month-old group. (**c**) Heat maps were generated to compare the 72-month-old group with the 3-month-old group. Each row of data represents a sample, with each line indicating a differential gene. The intensity of color, ranging from red to blue, corresponds to the expression level of the gene, with reddening indicating higher expression and blueness indicating lower expression. (**d**) Volcanic maps of the 72-month-old group compared to the 3-month-old group. Blue represents a significantly downregulated gene, red represents a significantly upregulated gene, Gray is not DEGs. LX and NX stand for the 72-month-old group and the 3-month-old group, respectively.

The DEGs of the 3-month-old group and 72-month-old group pectoral muscle tissues were compared. A total of 21,637 genes were detected in the muscle tissues (Figure 3a). A total of 819 DEGs were identified between the 3-month-old and 72-month-old groups (Figure 3b and Table S4). Among these, 422 genes were significantly upregulated, and 397 genes were significantly downregulated in the 72-month-old group compared to the 3-month-old group (Figure 3b and Table S4). The volcano plot in Figure 1b illustrates the distribution of these DEGs (Figure 3d). A heatmap of DEGs further demonstrates the consistent and distinct expression patterns between the two age groups (Figure 3c).

### 3.5. Go and Kegg Analysis of Degs

**Figure 4:**
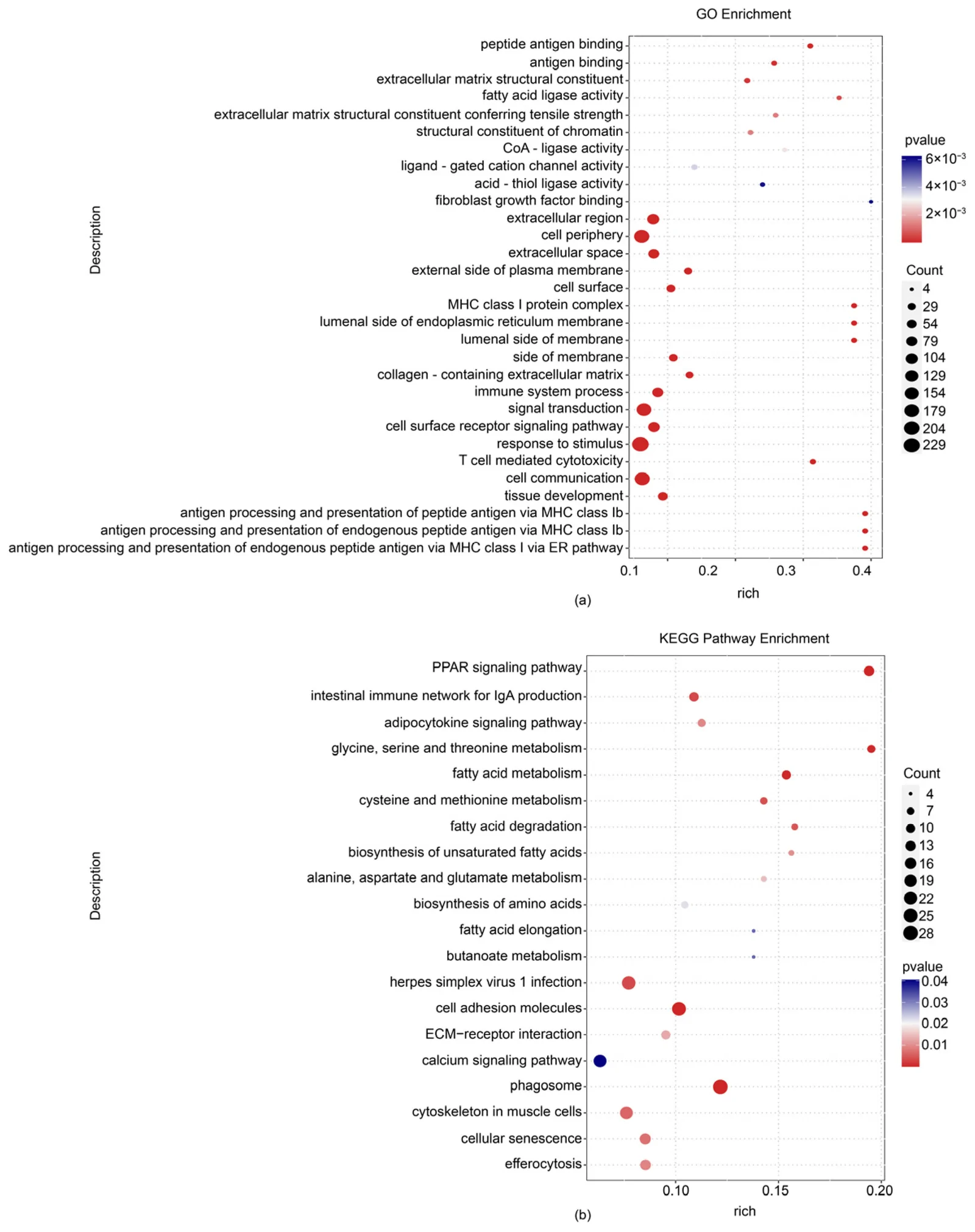
GO and KEGG enrichment of DEGs. (**a**) GO pathway assignment of the DEGs in 3-month-old and 72-month-old comparison groups. (**b**) KEGG pathway assignment of the DEGs in 3-month-old and 72-month-old comparison groups.

GO enrichment analysis revealed that the DEGs were significantly enriched in 815 GO terms, as depicted in Figure 4a and Table S5. The DEGs in the 3-month-old and 72-month-old comparison groups were notably enriched in pathways such as cell differentiation, lymphocyte-mediated immunity, cellular developmental processes, and regulation of lymphocyte-mediated immunity, among other GO terms.

Furthermore, KEGG analysis assigned the DEGs to 24 metabolic pathways. The pathway enrichment analysis, illustrated in Figure 4b and Table S6, showed that the DEGs in the 3-month-old and 72-month-old comparison groups were notably enriched in pathways such as glycine, serine, and threonine metabolism, fatty acid metabolism, intestinal immune network for IgA production, herpes simplex virus 1 infection, cysteine and methionine metabolism, and other KEGG terms.

### 3.6. Quantitative Real-Time PCR Validation

**Figure 5:**
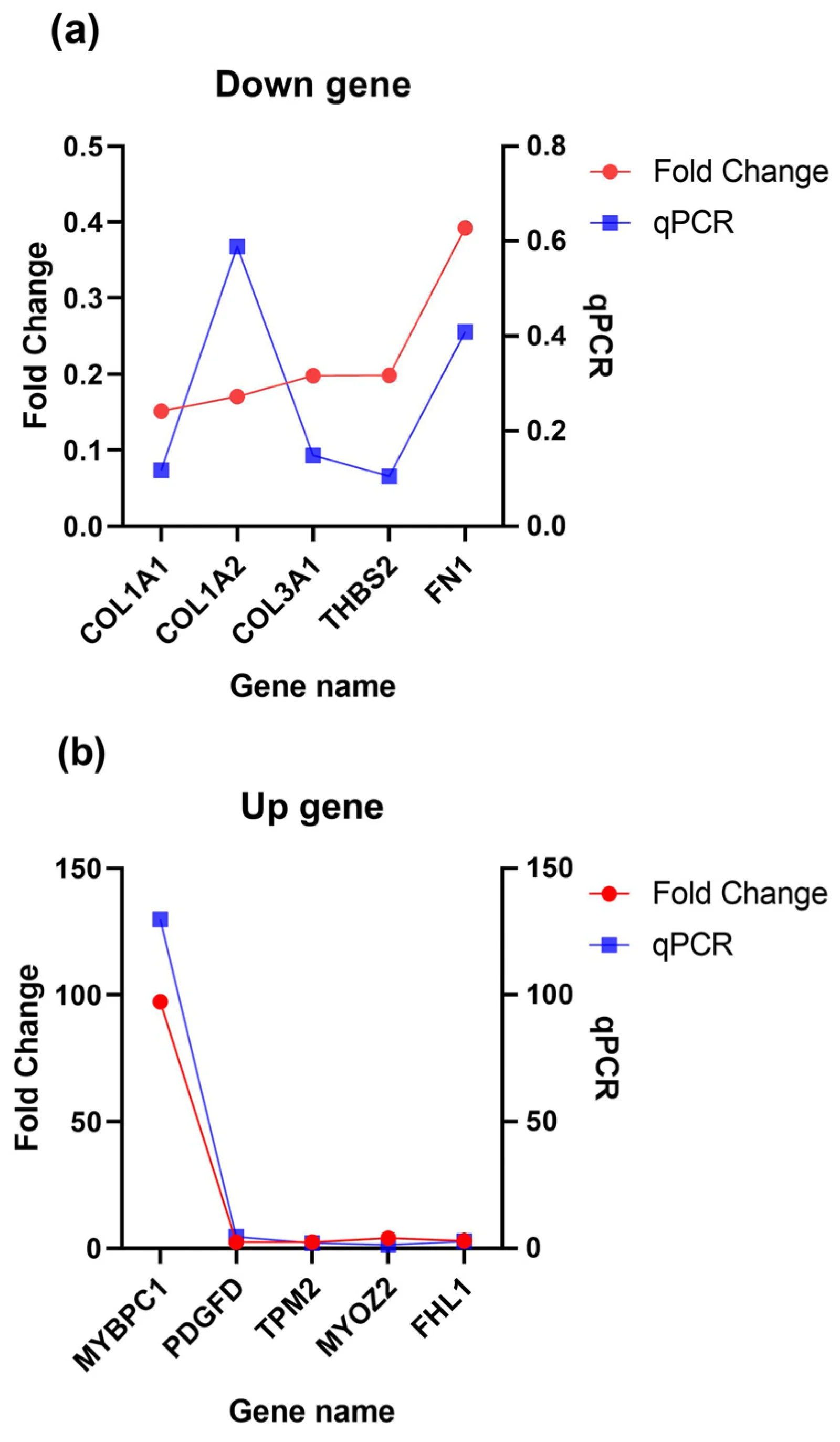
qRT-PCR validation of gene expression. (**a**) qPCR validation of downregulated gene expression. (**b**) qPCR validation of upregulated gene expression.

In order to verify the precision of the RNA-Seq data, a selection of eight DEGs (five upregulated (*PDGFD*, *MYBPC1*, *TPM2*, *MYOZ2,* and *FHL1* genes) and five downregulated (*COL1A2*, *THBS2*, *FN1*, *COL3A1*, and *COL1A1* genes)) was chosen for validation using qPCR. The results demonstrated that the relative expression patterns of all ten genes exhibited a high degree of consistency with the trends observed in the RNA-Seq analysis (Figure 5). The strong correlation between the two methods validates the reliability of the transcriptomic data.

## 4. Discussion

Skeletal muscle is a sizable and intricate tissue within the domain of animal organisms. Its growth and development are intricate biological processes, regulated by numerous genes and pathways [44,45]. In the context of the broiler industry, the extent of muscle growth is a critical factor in determining meat yield and economic returns [46,47]. With age, the structure, function, and metabolic characteristics of muscle undergo significant changes, which not only affect the physiological health of the animal but also profoundly alter the quality characteristics of the meat [48]. This study aimed to elucidate the molecular mechanisms underpinning the effects of aging on muscle development and meat quality in Dahen chickens through integrated phenotypic and transcriptomic analyses of the pectoralis major muscle at 3 and 72 months of age.

The results of this study demonstrated that broilers aged 72 months exhibited darker meat color, higher shear force, and greater IMF content. Concurrently, drip loss and cooking loss were found to be significantly lower in comparison to those of 3-month-old broilers. These changes were closely related to consumers’ sensory evaluation of meat quality. Increased shear force is frequently linked to diminished muscle tenderness; this phenomenon may be attributed to the interrelationship among collagen, compression, shear force, and tenderness of muscle [49]. Conversely, augmented IMF content is widely regarded as a key contributor to meat flavor and juiciness [31]. Reduced drip loss and cooking loss are indicative of superior water-holding capacity in aged chicken meat, which may be attributable to alterations in water distribution within and between muscle cells, in addition to modifications in protein structure.

At the level of muscle fiber characteristics, significant increases in muscle fiber diameter and area were observed in 72-month-old chickens compared to 3-month-old chickens, accompanied by a corresponding decrease in muscle fiber density. This finding indicates that the development of broiler breast muscle with age is predominantly driven by myofiber hypertrophy rather than hyperplasia, which is in accordance with the postnatal muscle growth pattern observed in avian and other vertebrates [50,51]. The number of muscle fibers is largely determined before and after hatching, and the postnatal increase in muscle mass is largely dependent on an increase in the size of existing muscle fibers [51]. The sustained hypertrophy of myofibers is a process of muscle maturation and functional adaptation, accompanied by the accumulation of myofibrillar proteins and adjustments in organelle composition. Collectively, these factors form the cytological basis of changes in meat mass.

GO and KEGG enrichment analyses demonstrated that DEGs were significantly enriched in muscle system process, muscle organ development, skeletal muscle cell differentiation, cytoskeleton in muscle cells, and ECM–receptor interaction signaling pathways related to muscle development. This finding suggests that remodeling of the extracellular matrix (ECM) is a central aspect of muscle aging. The expression of several ECM-related genes, including members of the collagen gene family (*COL1A1*, *COL1A2*, *COL3A1*), as well as *FN1* and *THBS2*, was found to be significantly reduced in the 72-month-old group. The ECM plays a pivotal role in maintaining the structural integrity of muscle tissues and mechanotransduction [52]. As the organism ages, excessive deposition and compositional changes in the ECM (particularly increased collagen cross-linking) lead to increased tissue fibrosis and stiffness, which directly explain the increased shear force observed in aged chickens. Enrichment of ECM-related pathways has been reported in a variety of studies of muscle development in a wide range of animals [53,54], suggesting that the dynamics of ECM regulation are a conserved mechanism during muscle growth and maturation.

In addition to alterations in the ECM, this study identified multiple genes encoding myogenic fiber- and sarcomere-associated proteins that exhibited differential expression across various age groups. These included *MYBPC1*, *TPM2*, *MYOZ2*, and *FHL1*. The products of these genes constitute the core components that form the muscle contractile unit, known as the myotome. The composition and structure of myofibrils are subject to constant adaptation and optimization from the juvenile stage to old age. Conversion of different myosin heavy chain isoforms has been identified as a hallmark of muscle fiber type specialization and maturation [55]. These changes in the expression of myosin genes are closely linked to the process of hypertrophy and maturation of the functional properties of muscle fibers, which collectively determine the contractile properties and ultimately the fleshy characteristics of the muscle [56].

KEGG analysis also revealed significant differences in multiple metabolic pathways across age groups, including amino acid metabolism (glycine, serine and threonine metabolism) and fatty acid metabolism. During the rapid growth phase at three months of age, muscles require large amounts of amino acids for protein synthesis, and energy metabolism is high to support the rapid growth of muscle fibers [54]. Conversely, at 72 months of age, the maturation period is characterized by a decline in muscle growth, a decrease in protein synthesis, and a shift in metabolic patterns from anabolic dominance to energy storage and maintenance. The present study found that the enrichment of fatty acid metabolism-related pathways coincided with the increased IMF content in aged chicken meat. This suggests that with age, there is an increase in the activity of lipid metabolism within muscle tissue, resulting in increased fat deposition. This phenomenon not only alters the manner in which muscle energy is stored but also exerts a substantial influence on the flavor and tenderness of meat [57]. In this study, phenotypic and transcriptomic data were systematically integrated to construct a molecular map of pectoral muscle development and meat quality changes in broiler chickens across the transition from youthful to senescent stages. The findings are expected to advance the current understanding of the biological processes governing avian muscle growth and aging. Moreover, they provide novel molecular targets and a theoretical basis for the targeted improvement of meat quality in the poultry industry. Future investigations that incorporate additional intermediate age time points would be valuable for delineating the continuous trajectory of muscle development and for further validating the dynamic molecular alterations observed throughout the ageing process.

## 5. Conclusions

In conclusion, a comparison of 3-month-old and 72-month-old Dahen broilers revealed age-related changes in breast muscle. Aged birds exhibited reduced tenderness, increased intramuscular fat, improved water-holding capacity, myofiber hypertrophy, and decreased fiber density. Transcriptome analysis revealed 819 differentially expressed genes enriched in ECM–receptor interaction, cytoskeleton, and metabolic pathways. Key genes (*COL1A2*, *COL3A1*, *FN1*, *THBS2*, *MYBPC1*, *TPM2*, *MYOZ2*, *FHL1*) demonstrated significant alterations in expression with age. These findings provide molecular insights into muscle aging and potential targets for improving meat quality.

## Figures and Tables

**Table 1:** Meat quality analysis of Dahen chickens at different ages.

Meat Quality	3-Month-Old Group	72-Month-Old Group
meat color	70.73 ± 4.35	84.86 ± 2.51 **
pH	6.09 ± 0.26	6.62 ± 0.17 **
shear force, (kgf)	4.92 ± 0.46	6.51 ± 0.23 **
drip loss, (%)	2.49 ± 0.55 **	1.14 ± 0.54
cooking loss, (%)	28.37 ± 6.54 **	21.82 ± 1.86
IMF, (%)	1.43 ± 0.04	1.96 ± 0.29 **
IMP, (mg/g)	3.23 ± 0.19 **	2.27 ± 0.18

The IMF is the intramuscular fat, the IMP is the inosine monophosphate, the pH is pH value of the pectoral muscle was measured after of slaughter. ** is *p* < 0.01.

**Table 2:** Muscle fiber characteristics of Dahen chickens at different ages.

Muscle Fiber Characteristics	3-Month-Old Group	72-Month-Old Group
muscle fiber area, mm^2^	2581.18 ± 385.86	5165.46 ± 828.25 **
muscle fiber diameter, mm	52.05 ± 4.71	71.83 ± 7.69 **
muscle fiber density, root/mm^2^	560.93 ± 95.21 **	300.50 ± 68.78

The muscle fiber area is defined as the actual area of the selected muscle fibers that has been calculated directly (400×). The muscle fiber diameter is defined as the short diameter of the muscle fiber (400×). The muscle fiber density refers to the number of muscle fibers present within an area at 400× magnification (400 times magnification covers an area of 53,660.72 µm^2^). ** is *p* < 0.01.

## Data Availability

The raw data supporting the conclusions of this article will be made available by the authors on request.

## Supplementary Materials

The following supporting information can be downloaded at: https://www.mdpi.com/article/10.3390/ani16172743/s1, Table S1: Composition and nutrient levels of the basal diet (air-dry basis); Table S2: Primers for qRT-PCR; Table S3: Summary of RNA-seq data; Table S4: Detailed information on DEGs between the LX and NX comparison group in Dahen chickens; Table S5: Enriched GO terms for DEGs identified between the LX and NX comparison group; Table S6: Enriched KEGG terms for DEGs identified between the LX and NX comparison group.

## References

[1] Schnyder S., Handschin C. (2015). Skeletal muscle as an endocrine organ: PGC-1α, myokines and exercise. Bone.

[2] Hong J.Y., Raza S.H.A., Liu M.Q., Li M.Y., Ruan J.R., Jia J.J., Ge C.R., Cao W.N. (2024). Association analysis of transcriptome and quasi-targeted metabolomics reveals the regulation mechanism underlying broiler muscle tissue development at different levels of dietary guanidinoacetic acid. Front. Vet. Sci..

[3] Liu L., Cui H.X., Dong N.X., Zhu X.H., Li S.H., Ma X., Niu D. (2025). Effects of phosphatidylethanolamine on intramuscular fat deposition and key gene identification by transcriptome sequencing in broiler chickens. Poult. Sci..

[4] Schiaffino S., Mammucari C. (2011). Regulation of skeletal muscle growth by the IGF1-Akt/PKB pathway: Insights from genetic models. Skelet. Muscle.

[5] Li Z.X., Xu Y., Lin Y.Q. (2018). Transcriptome analyses reveal genes of alternative splicing associated with muscle development in chickens. Gene.

[6] Gao M.K., Han Q.Q., Zhu Q., Li D.L., Li X.M., Lv Z.P., Guo Y.M. (2025). Maternal effects of choline on skeletal muscle development and intramuscular fat deposition in broiler offspring. Poult. Sci..

[7] Vafaeinia M., Yalcin S. (2025). Temperature manipulation during incubation: Effect on embryo development and incidence of white striping and expression of related genes in broiler chickens from two commercial breeds. Br. Poult. Sci..

[8] Petracci M., Soglia F., Madruga M., Carvalho L., Ida E., Estévez M. (2019). Wooden-Breast, White striping, and spaghetti meat: Causes, consequences and consumer perception of emerging broiler meat abnormalities. Compr. Rev. Food Sci. Food Saf..

[9] Baldi G., Soglia F., Mazzoni M., Sirri F., Canonico L., Babini E., Laghi L., Cavani C., Petracci M. (2018). Implications of white striping and spaghetti meat abnormalities on meat quality and histological features in broilers. Animal.

[10] Petracci M., Bianchi M., Cavani C. (2009). The European perspective on pale, soft, exudative conditions in poultry. Poult. Sci..

[11] Giampietro-Ganeco A., de Barba B.R., Galvao A.C., Trindade M.A., Borba H., Stefani L.M., Boiago M.M., Robazza W.D. (2024). Meat quality evaluation of breast fillets of different strains of chicken broilers with deep pectoral myopathy. Int. J. Food Sci. Tech..

[12] Jlali M., Gigaud V., Métayer-Coustard S., Sellier N., Tesseraud S., Le Bihan-Duval E., Berri C. (2012). Modulation of glycogen and breast meat processing ability by nutrition in chickens: Effect of crude protein level in 2 chicken genotypes. J. Anim. Sci..

[13] Srinual O., Srinual P., Khetanun K., Loungmoon P., Pintalerd N., Chaiyaso T., Yakul K., Kanmanee C., Tapingkae W. (2026). Influence of dietary fermented coffee cherry pulp on growth performance, meat quality, and cecal microbiota in Thai Native Chickens. Animals.

[14] Choi J., Kong B.Y.W., Bowker B.C., Zhuang H., Kim W.K. (2023). Nutritional Strategies to Improve Meat Quality and Composition in the Challenging Conditions of Broiler Production: A Review. Animals.

[15] You Z.R., Bai Y.L., Bo D.D., Feng Y.Q., Shen J.M., Wang Y.Y., Li J., Bai Y.Y. (2024). A review of taste-active compounds in meat: Identification, influencing factors, and taste transduction mechanism. J. Food Sci..

[16] Cartee G.D., Hepple R.T., Bamman M.M., Zierath J.R. (2016). Exercise Promotes healthy aging of skeletal muscle. Cell Metab..

[17] Narici M.V., Maffulli N. (2010). Sarcopenia: Characteristics, mechanisms and functional significance. Br. Med. Bull..

[18] Yuan S., Larsson S.C. (2023). Epidemiology of sarcopenia: Prevalence, risk factors, and consequences. Metabolism.

[19] Linge J., Birkenfeld A.L., Neeland I.J. (2024). Muscle mass and glucagon-like peptide-1 receptor agonists: Adaptive or maladaptive response to weight loss?. Circulation.

[20] Neeland I.J., Linge J., Birkenfeld A.L. (2024). Changes in lean body mass with glucagon-like peptide-1-based therapies and mitigation strategies. Diabetes Obes. Metab..

[21] Chodzko-Zajko W.J., Proctor D.N., Singh M.A.F., Minson C.T., Nigg C.R., Salem G.J., Skinner J.S. (2009). American College of Sports Medicine position stand. Exercise and physical activity for older adults. Med. Sci. Sports Exerc..

[22] Serra A., Foggi G., Buccioni A., Amarie R.E., Tinagli S., Scicutella F., Casarosa L., Secci G., Mantino A., Mele M. (2024). Dietary supplementation with natural antioxidants: Assessment of growth performance and meat quality in broiler chickens. Poult. Sci..

[23] Xu J., Li M.Y., Li R., Zhang Z., Ma Y., Tao R., Zou L.R., Wang J., Wen L.X., Li R.F. (2025). Effect of areca nut extracts on growth performance, slaughtering performance, and meat quality of broiler chickens. Front. Vet. Sci..

[24] Freick M., Herzog M., Rump S., Vogt I., Weber J., John W., Schreiter R. (2022). Incubation characteristics, growth performance, carcass characteristics and meat quality of Saxonian Chicken and German Langshan bantam breeds in a free-range rearing system. Vet. Med. Sci..

[25] Deng S.L., Liu R., Li C.B., Xu X.L., Zhou G.H. (2022). Meat quality and flavor compounds of soft-boiled chickens: Effect of Chinese yellow-feathered chicken breed and slaughter age. Poult. Sci..

[26] Yuan C.Y., Jiang Y., Wang Z.X., Chen G.H., Bai H., Chang G.B. (2022). Indigenous, yellow-feathered chickens body measurements, carcass traits, and meat quality depending on marketable age. Animals.

[27] Gao J., Chen N., Cheng J., Yuan Z.N., Zeng W.X., Lu H.Z., Zhang T., Wang L. (2025). Influence of slaughter age on meat quality, metabolite and volatile organic compound changes in Lueyang black-bone chickens. Poult. Sci..

[28] Gai K., Ge Y., Liu D.P., Zhang H., Cong B.L., Guo S.H., Liu Y.Z., Xing K., Qi X.L., Wang X.G. (2023). Identification of key genes related to intramuscular fat deposition in Beijing-You chicken by mRNA and miRNA transcriptome analysis. Poult. Sci..

[29] Li P., Yang Y.F., Ning B., Tian Y.M., Wang L., Zeng W.X., Lu H.Z., Zhang T. (2025). Transcriptome analysis of multiple tissues and identification of tissue-specific genes in Lueyang black-bone chicken. Poult. Sci..

[30] Zhong H.A., Zhu L., Kong X.Y., Zhang K., Tang L., Zhang H., Zhang B., Gou X. (2025). Characterization and comparative transcriptomic analysis of high-altitude adaptation in Tibetan chicken using RNA-sequencing. Poult. Sci..

[31] Liu L., Liu X.J., Cui H.X., Liu R.R., Zhao G.P., Wen J. (2019). Transcriptional insights into key genes and pathways controlling muscle lipid metabolism in broiler chickens. BMC Genom..

[32] Wu Y.Q., Wang Y.L., Yin D.F., Mahmood T., Yuan J.M. (2020). Transcriptome analysis reveals a molecular understanding of nicotinamide and butyrate sodium on meat quality of broilers under high stocking density. BMC Genom..

[33] Xiong X., Yu C.L., Qiu M.H., Zhang Z.R., Hu C.M., Zhu S.L., Yang L., Peng H., Song X.Y., Chen J.L. (2024). Genomic and gut microbiome evaluations of growth and feed efficiency traits in broilers. Animals.

[34] Li X.D., Chen Z.M., Li J.T. (2024). Effects of guanidine acetic acid on the growth and slaughter performance, meat quality, antioxidant capacity, and cecal microbiota of broiler chickens. Vet. Sci..

[35] Wang Q.X., Wang L., Li L.W., Sun M.Q., Li P., Yu Y., Zhang Y.H., Xu Z.Y., Gao P., Ma J.Y. (2024). Effects of dietary supplementation of fermented Artemisia argyi on growth performance, slaughter performance, and meat quality in broilers. Poult. Sci..

[36] Ding Y.A., Jiang X.D., Yao X.F., Zhang H.H., Song Z.H., He X., Cao R. (2021). Effects of feeding fermented mulberry leaf powder on growth performance, slaughter performance, and meat quality in chicken broilers. Animals.

[37] Guo X., Wang J.X., Chen H., Su H., Wang Z.C., Wan Y., Huang Y.Y., Jiang R.S. (2019). Effects of exercise on carcass composition, meat quality, and mRNA expression profiles in breast muscle of a Chinese indigenous chicken breed. Poult. Sci..

[38] Xu T.S., Jiang Q.C., Xu C.H., Xiao Z.P., Zheng X.L., Gu L.H. (2025). Exploring the effects of feeding methods on the growth and meat flavor of Wenchang chicken. Poult. Sci..

[39] Dobin A., Davis C.A., Schlesinger F., Drenkow J., Zaleski C., Jha S., Batut P., Chaisson M., Gingeras T.R. (2013). STAR: Ultrafast universal RNA-seq aligner. Bioinformatics.

[40] Li B., Dewey C.N. (2011). RSEM: Accurate transcript quantification from RNA-Seq data with or without a reference genome. BMC Bioinform..

[41] Love M.I., Huber W., Anders S. (2014). Moderated estimation of fold change and dispersion for RNA-seq data with DESeq2. Genome Biol..

[42] Yu G.C., Wang L.G., Han Y.Y., He Q.Y. (2012). clusterProfiler: An R package for comparing biological themes among gene clusters. OMICS J. Integr. Biol..

[43] Livak K.J., Schmittgen T.D. (2001). Analysis of relative gene expression data using real-time quantitative PCR and the 2 (-Delta Delta C(T)) Method. Methods.

[44] Du P.F., Zhang X.L., Wang Z.Y., Su C.Q., Zhang H.Y., Zheng R., Si X.M., Chen W., Huang Y.Q. (2026). Temporal dynamics of insulin-responsive long non-coding RNAs in chicken skeletal muscle: Unraveling LncFKBP5 as a dual regulator of myogenesis and glucose homeostasis. Int. J. Biol. Macromol..

[45] Wu L.T., Peng L.L., Zhao X.L. (2026). Regulation of skeletal muscle development and metabolism in broiler chickens by Urolithin A through threonine kinase 1 pathway activation. Poult. Sci..

[46] Song J.L., Lv Z.P., Guo Y.M. (2025). Research advances in intramuscular fat deposition and chicken meat quality: Genetics and nutrition. J. Anim. Sci. Biotechnol..

[47] Xu H.D., Li T., Wang Z., Adu-Asiamah P., Leng Q.Y., Zheng J.H., Zhao Z.H., An L.L., Zhang X.Q., Zhang L. (2019). Roles of chicken growth hormone receptor antisense transcript in chicken muscle development and myoblast differentiation. Poult. Sci..

[48] Ren W.L., Wang J.W., Zeng Y.Q., Wang T.L., Sun Z.W., Meng J., Yao X.K. (2024). Investigating age-related differences in muscles of Kazakh horse through transcriptome analysis. Gene.

[49] Hopkins D.L., Allingham P.G., Colgrave M., van de Ven R.J. (2013). Interrelationship between measures of collagen, compression, shear force and tenderness. Meat Sci..

[50] Berri C., Le Bihan-Duval E., Debut M., Sante-Lhoutellier V., Baeza E., Gigaud V., Jego Y., Duclos M.J. (2007). Consequence of muscle hypertrophy on characteristics of Pectoralis major muscle and breast meat quality of broiler chickens. J. Anim. Sci..

[51] Scheuermann G.N., Bilgili S.F., Tuzun S., Mulvaney D.R. (2004). Comparison of chicken genotypes: Myofiber number in pectoralis muscle and myostatin ontogeny. Poult. Sci..

[52] Mahdy M.A.A. (2019). Skeletal muscle fibrosis: An overview. Cell Tissue Res..

[53] Lou M.Y., Zhang S.H., Yang W.X., Li S., Cao H.G., Zhang Z.J., Ling Y.H. (2025). Transcriptome analysis revealed the mechanism of skeletal muscle growth and development in different hybrid sheep. Anim. Biosci..

[54] Xue Q., Zhang G.X., Li T.T., Ling J.J., Zhang X.Q., Wang J.Y. (2017). Transcriptomic profile of leg muscle during early growth in chicken. PLoS ONE.

[55] Saneyasu T., Tsuchihashi T., Kitashiro A., Tsuchii N., Kimura S., Honda K., Kamisoyama H. (2017). The IGF-1/Akt/S6 pathway and expressions of glycolytic myosin heavy chain isoforms are upregulated in chicken skeletal muscle during the first week after hatching. Anim. Sci. J..

[56] Guo X., Wang H., Liu M., Xu J.M., Liu Y.N., Zhang H., He X.X., Wang J.X., Wei W., Ren D.L. (2024). Weighted gene co-expression network analysis identifies important modules and hub genes involved in the regulation of breast muscle yield in broilers. Anim. Biosci..

[57] Zhao J.Y., Ge X.H., Li T., Yang M., Zhao R.H., Yan S.X., Wu H., Liu Y., Wang K., Xu Z.Q. (2024). Integrating metabolomics and transcriptomics to analyze the differences of breast muscle quality and flavor formation between Daweishan mini chicken and broiler. Poult. Sci..

