# Supplementary material for: Effects of Aging on Muscle Development Based on Transcriptome Sequencing in Dahen Broilers
Source: Animals (Basel). 2026 Sep 2;16(17):2743. doi: 10.3390/ani16172743 (PMC13565069; doi:10.3390/ani16172743)
Supplement: Supplementary file 1 [file animals-16-02743-s001.zip › animals-4532554-supplementary.pdf]

Table S1. Composition and nutrient levels of the basal diet (air-dry basis).

| Items | 1 to 30days of age | 30 days of age to 72 months of age |
|---|---|---|
| Ingredients | | |
| Corn, % | 61.69 | 69.46 |
| Soybean meal, % | 25.6 | 17 |
| Corn gluten meal, % | 4 | 4 |
| Corn DDGS, % | 3 | 3 |
| Soybean oil, % | 1 | 2.5 |
| Limestone, % | 1.4 | 1.4 |
| CaHPO4, % | 1.5 | 1 |
| NaCl, % | 0.26 | 0.26 |
| Lys, % | 0.38 | 0.3 |
| Met, % | 0.2 | 0.11 |
| Thr, % | 0.05 | 0.06 |
| Choline chloride, % | 0.09 | 0.1 |
| Premix, % | 0.83[1)] | 0.81[2)] |
| Total, % | 100 | 100 |
| Nutrient levels[3)] | | |
| ME,（MJ/kg） | 12.21 | 12.93 |
| CP, % | 19.84 | 16.15 |
| Ca, % | 0.92 | 0.74 |
| TP, % | 0.63 | 0.48 |
| Lys, % | 1.12 | 0.93 |
| Met, % | 0.47 | 0.42 |

[1)]The premix provided the following per kilogram of diet: vitamin A, 9,900 IU; vitamin $D_3$, 1,800 IU; vitamin E, 38 IU; vitamin $K_3$, 3.15 mg; vitamin $B_1$, 2.7 mg; vitamin $B_2$, 6.75 mg; vitamin $B_6$, 4.5 mg; vitamin $B_{12}$, 31.5 μg; biotin, 0.23 mg; pantothenic acid, 15.75 mg; folic acid, 1.1 mg; nicotinic acid, 45 mg; Fe, 150 mg; Cu, 19 mg; Mn, 132 mg; Zn, 110 mg; I, 0.41 mg; Se, 0.49 mg.

[2)]The premix provided the following per kilogram of diet: vitamin A, 8,800 IU; vitamin $D_3$, 1,600 IU; vitamin E, 34 IU; vitamin $K_3$, 2.8 mg; vitamin $B_1$, 2.4 mg; vitamin $B_2$, 6.0 mg; vitamin $B_6$, 4.0 mg; vitamin $B_{12}$, 28 μg; biotin, 0.2 mg; pantothenic acid, 14 mg; folic acid, 1.0 mg; nicotinic acid, 40 mg; Fe, 135 mg; Cu, 15 mg; Mn, 110 mg; Zn, 114 mg; I, 0.53 mg; Se, 0.5 mg.

[3)]ME was a calculated value, while the others were measured values.

Table S2. Primers for qRT-PCR.

| Gene ID | Gene name | Primer sequence (5′→3′) | Size(bp) |
|---|---|---|---|
| ENSGALG00010025092 | COL1A1 | F-CGGGCTGACGTACAACGATA<br>R-GGATCGGTACCGTCGACATC | 180 |
| ENSGALG00010003307 | COL1A2 | F-TGTCTCTAGGGTCCACCAGG<br>R-TGCATGTCTTACTCCGCCAA | 105 |
| ENSGALG00010013170 | COL3A1 | F-TGCACAGAGAGTCTCATGTCT<br>R-TACTCACCGTCCTGTTGTGC | 118 |
| ENSGALG00010004760 | THBS2 | F-GAAGACGTGGACCTTGCTGA<br>R-GGACCCTTTCGCCACATACA | 171 |
| ENSGALG00010011984 | FN1 | F-TTACCTGGGGAACACGTTGG<br>R-ACCAACTTACGTTCGGGCTT | 84 |
| ENSGALG00010011558 | MYBPC1 | F-AAATGCTCTACCGCCTCCAG<br>R-AGTCCCATGCCATTCTTTTGT | 82 |
| ENSGALG00010005249 | PDGFD | F-GCAGTTACCCCAGGAACCTC<br>R-TTCCACACCAGAGGACTTGC | 145 |
| ENSGALG00010010970 | TPM2 | F-GGGCACAGAAGATGAGGTGG<br>R-CTACGTACGTCCGTGGCTTT | 96 |
| ENSGALG00010005510 | MYOZ2 | F-GAGACGTCAGCGGAGATCTG<br>R-CCAGGCTAGTGCAACAGTGT | 115 |
| ENSGALG00010014929 | FHL1 | F-TCGCTGCAAGGCAAGAAGTA<br>R-AGTTACCTTGGAGTCAGCGC | 126 |
| ENSGALG00000014442 | GAPDH | F-CCTCTCTGGCAAAGTCCAAG<br>R-CATCTGCCCATTTGATGTTG | 200 |

Table S3. Summary of RNA-seq data.

| Sample ID | Obtained Reads(bp) | Obtained Base(bp) | Q20(%) | Q30(%) | GC(%) | Unique mapped read ratio(%) |
|---|---|---|---|---|---|---|
| LX1 | 40051840 | 5975999436 | 99.01 | 96.59 | 49.91 | 90.37 |
| LX2 | 40059978 | 5954966798 | 99.23 | 97.33 | 49.57 | 92.57 |
| LX3 | 40053262 | 5969180956 | 99.22 | 97.23 | 48.71 | 91.20 |
| NX1 | 40019616 | 5972593556 | 99.14 | 96.97 | 49.88 | 89.64 |
| NX2 | 40018538 | 5964807534 | 99.20 | 97.21 | 48.47 | 90.62 |
| NX3 | 40020160 | 5969707533 | 99.24 | 97.27 | 49.63 | 91.05 |

LX1, LX2 and LX3 is the 72-month-old group, NX1, NX2 and NX3 is 3-month-old group.

Table S4. Detailed information on DEGs between the LX and NX comparison group in Dahen chickens.

| Gene ID | Gene name | Log2FoldChange |
|---|---|---|
| ENSGALG00010004729 | SBK3 | -9.168036308 |
| ENSGALG00010021433 | - | -9.14479846 |
| ENSGALG00010017604 | GATM | -8.114989284 |
| ENSGALG00010014325 | - | -8.106210467 |
| novel395 | - | -8.010658354 |
| ENSGALG00010024546 | KY | -7.879844873 |
| novel281 | - | -7.845917996 |
| ENSGALG00010000591 | SBK2 | -6.995185248 |
| ENSGALG00010013099 | SIM2 | -6.761539154 |
| ENSGALG00010012046 | LOXHD1 | -6.691384773 |
| ENSGALG00010010315 | - | -6.468441226 |
| ENSGALG00010013295 | CA3B | -6.399196025 |
| ENSGALG00010011032 | - | -6.315567864 |
| ENSGALG00010005920 | - | -6.136535866 |
| ENSGALG00010021081 | KCNH6 | -5.950520456 |
| ENSGALG00010003972 | PENK | -5.932608291 |
| ENSGALG00010000869 | - | -5.898969265 |
| novel525 | - | -5.674569055 |
| ENSGALG00010008091 | - | -5.658772144 |
| ENSGALG00010022766 | - | -5.62725829 |
| ENSGALG00010009589 | - | -5.595082656 |
| ENSGALG00010016390 | ACSM4 | -5.565756575 |
| novel204 | - | -4.987263283 |
| ENSGALG00010011814 | CYLY | -4.898120174 |
| ENSGALG00010023157 | STOX1 | -4.670489016 |
| ENSGALG00010029214 | PROCA1 | -4.609287231 |
| ENSGALG00010018361 | ASIC4 | -4.604872125 |
| ENSGALG00010009112 | - | -4.477342192 |
| ENSGALG00010011767 | - | -4.383221669 |
| ENSGALG00010006441 | - | -4.375548521 |
| ENSGALG00010005544 | AQP4 | -4.329492517 |
| ENSGALG00010006037 | METTL21EP | -4.303398364 |
| ENSGALG00010002693 | MSC | -4.228342984 |
| ENSGALG00010005444 | ACTC1 | -4.07143435 |
| ENSGALG00010028873 | TMEM8C | -3.936161779 |
| ENSGALG00010023212 | RIMBP2 | -3.868447149 |
| ENSGALG00010025194 | - | -3.868261731 |
| ENSGALG00010020565 | - | -3.817955665 |
| ENSGALG00010025931 | TMEM136 | -3.806538549 |
| ENSGALG00010011482 | DMRT2 | -3.800830263 |
| ENSGALG00010024834 | INSIG1 | -3.799501322 |
| ENSGALG00010009106 | THSD7B | -3.72754573 |

| | | |
|---|---|---|
| ENSGALG00010012091 | - | -3.72130054 |
| ENSGALG00010016453 | MYCL | -3.630437705 |
| novel512 | - | -3.53978252 |
| ENSGALG00010023545 | PTPRT | -3.539245352 |
| ENSGALG00010024697 | IGF2BP1 | -3.519252283 |
| ENSGALG00010016825 | CD164L2 | -3.516656507 |
| ENSGALG00010001125 | CLRN1 | -3.511146986 |
| ENSGALG00010021367 | GRIP2 | -3.491560423 |
| ENSGALG00010014648 | C1QTNF6 | -3.419772964 |
| ENSGALG00010015097 | MFSD2A | -3.416330001 |
| ENSGALG00010015960 | CHRNA9 | -3.292735759 |
| ENSGALG00010017645 | SYNC | -3.266018477 |
| ENSGALG00010026292 | - | -3.243766193 |
| ENSGALG00010002358 | FREM2 | -3.226362606 |
| ENSGALG00010006275 | - | -3.217951402 |
| ENSGALG00010014033 | SLC38A4 | -3.216023299 |
| ENSGALG00010026410 | - | -3.200657369 |
| ENSGALG00010003552 | PI15 | -3.190576548 |
| ENSGALG00010000797 | CMBL | -3.156344978 |
| ENSGALG00010014980 | - | -3.154678539 |
| ENSGALG00010028014 | THEM4 | -3.136285211 |
| novel547 | - | -3.116116375 |
| ENSGALG00010021183 | CFAP100 | -3.083496805 |
| ENSGALG00010011206 | - | -3.076978046 |
| ENSGALG00010005834 | - | -3.069816987 |
| ENSGALG00010017591 | ESR2 | -3.054870307 |
| ENSGALG00010019221 | VASH2 | -3.054648414 |
| ENSGALG00010006279 | HACD1 | -3.042158278 |
| ENSGALG00010004172 | ST8SIA6 | -3.037825318 |
| ENSGALG00010004996 | GPR158 | -3.036249362 |
| ENSGALG00010001192 | MLF1 | -3.026842074 |
| ENSGALG00010015306 | BCAT1 | -3.003347909 |
| ENSGALG00010012836 | - | -2.967257793 |
| ENSGALG00010016976 | IL9R | -2.961279784 |
| ENSGALG00010021083 | SCD | -2.916618074 |
| ENSGALG00010002062 | GSTA4 | -2.868111094 |
| ENSGALG00010020196 | - | -2.866203099 |
| ENSGALG00010010984 | MAT1A | -2.833838614 |
| ENSGALG00010002373 | POSTN | -2.816942758 |
| ENSGALG00010009600 | LRRC17 | -2.808627029 |
| ENSGALG00010006096 | EDIL3 | -2.807917839 |
| ENSGALG00010026773 | - | -2.801133797 |
| ENSGALG00010017786 | KAZALD1 | -2.787822852 |
| ENSGALG00010018463 | - | -2.748449058 |

| | | |
|---|---|---|
| ENSGALG00010006348 | - | -2.747940676 |
| ENSGALG00010001858 | ADAMTS20 | -2.741692622 |
| ENSGALG00010028562 | ACSBG2 | -2.734349305 |
| ENSGALG00010025092 | COL1A1 | -2.721937874 |
| ENSGALG00010012114 | TAL2 | -2.693699329 |
| ENSGALG00010008515 | FAM19A5 | -2.667422069 |
| ENSGALG00010013382 | ASB12 | -2.655533232 |
| ENSGALG00010006443 | - | -2.652575375 |
| ENSGALG00010017399 | CPXM1 | -2.649162474 |
| ENSGALG00010020181 | LEUTX | -2.632194505 |
| ENSGALG00010019483 | SCUBE2 | -2.626247499 |
| ENSGALG00010012083 | MGP | -2.585883274 |
| ENSGALG00010006289 | SKIDA1 | -2.577960759 |
| ENSGALG00010004760 | THBS2 | -2.550686626 |
| ENSGALG00010023996 | MYO7B | -2.540551375 |
| ENSGALG00010013232 | FHL2 | -2.517817923 |
| ENSGALG00010003541 | CNR1 | -2.49308809 |
| ENSGALG00010022932 | OPCML | -2.479083874 |
| ENSGALG00010010390 | KCNK3 | -2.476873444 |
| ENSGALG00010025215 | MYOG | -2.467432364 |
| ENSGALG00010028364 | MHM2 | -2.461164147 |
| ENSGALG00010028027 | NIT1 | -2.451295496 |
| ENSGALG00010003652 | MYCN | -2.448880121 |
| novel612 | - | -2.435686748 |
| ENSGALG00010005241 | C4H4orf54 | -2.42891829 |
| ENSGALG00010009620 | TMOD1 | -2.410047277 |
| novel509 | - | -2.404575278 |
| ENSGALG00010028056 | SLC25A25 | -2.400587699 |
| ENSGALG00010015589 | FRMD3 | -2.373704187 |
| ENSGALG00010012390 | MMP16 | -2.35858152 |
| ENSGALG00010029671 | GRIN2C | -2.346649747 |
| ENSGALG00010009400 | CNTFR | -2.345660604 |
| ENSGALG00010005547 | LRP11 | -2.339933236 |
| ENSGALG00010003307 | COL1A2 | -2.335239695 |
| ENSGALG00010000646 | - | -2.332830796 |
| ENSGALG00010013170 | COL3A1 | -2.33276788 |
| ENSGALG00010003382 | ASNS | -2.319088291 |
| ENSGALG00010021247 | GNAO1 | -2.318880156 |
| ENSGALG00010014598 | - | -2.31855646 |
| ENSGALG00010010285 | - | -2.302788677 |
| ENSGALG00010023193 | PPDPF | -2.30152908 |
| ENSGALG00010006584 | - | -2.30004054 |
| ENSGALG00010024690 | TUBA1C | -2.268288638 |
| ENSGALG00010019323 | LRIG1 | -2.264325505 |

| | | |
|---|---|---|
| ENSGALG00010028881 | SARDH | -2.255499825 |
| ENSGALG00010015104 | CNGA2 | -2.253101495 |
| ENSGALG00010010603 | - | -2.245078546 |
| ENSGALG00010024463 | FBXL22 | -2.225659629 |
| ENSGALG00010007865 | GABRR3 | -2.205721354 |
| ENSGALG00010012978 | HSD17B7 | -2.193348654 |
| ENSGALG00010028502 | PLEKHO1 | -2.184419063 |
| ENSGALG00010019325 | SLITRK4 | -2.179171675 |
| ENSGALG00010015068 | ESM1 | -2.178697937 |
| ENSGALG00010010279 | CCSAP | -2.175418005 |
| ENSGALG00010022579 | TNFAIP8L3 | -2.16761059 |
| ENSGALG00010005553 | PPP1R14C | -2.159025305 |
| ENSGALG00010008177 | NREP | -2.156191016 |
| ENSGALG00010004980 | FNDC1 | -2.15054024 |
| ENSGALG00010003543 | SOX11 | -2.143759145 |
| ENSGALG00010017482 | CORO2B | -2.140975012 |
| ENSGALG00010023369 | ACE | -2.136825049 |
| ENSGALG00010012915 | SELP | -2.136059716 |
| ENSGALG00010014087 | - | -2.127583655 |
| ENSGALG00010001476 | IGF2BP3 | -2.102753513 |
| ENSGALG00010027200 | G0S2 | -2.102683882 |
| ENSGALG00010021871 | - | -2.097263523 |
| ENSGALG00010014060 | - | -2.092306634 |
| ENSGALG00010026800 | - | -2.062950233 |
| ENSGALG00010018391 | STAC | -2.050993874 |
| ENSGALG00010014704 | - | -2.046619691 |
| ENSGALG00010029222 | TRAF4 | -2.045933114 |
| ENSGALG00010019591 | HSP90AB1 | -2.043193838 |
| ENSGALG00010026036 | TUB5A | -2.037863143 |
| ENSGALG00010029318 | C17orf58 | -2.036014987 |
| ENSGALG00010001854 | CHRDL2 | -2.030125507 |
| ENSGALG00010029496 | TTYH1 | -2.022497018 |
| ENSGALG00010010461 | SLC7A5 | -2.021193927 |
| ENSGALG00010017563 | ALK | -2.015927887 |
| ENSGALG00010012958 | OLFML2B | -2.008092071 |
| ENSGALG00010000764 | - | -2.002866555 |
| ENSGALG00010024001 | - | -2.000473683 |
| ENSGALG00010017671 | ANTXR1 | -1.987844715 |
| ENSGALG00010012229 | FSBP | -1.986046237 |
| ENSGALG00010023625 | - | -1.98333529 |
| ENSGALG00010016399 | ACSM5 | -1.963618278 |
| ENSGALG00010023453 | ARHGAP33 | -1.953508347 |
| ENSGALG00010011171 | - | -1.948908897 |
| ENSGALG00010023748 | - | -1.94753944 |

| | | |
|---|---|---|
| ENSGALG00010002840 | SRPX | -1.933193683 |
| ENSGALG00010006018 | - | -1.91765391 |
| ENSGALG00010004705 | - | -1.915537921 |
| ENSGALG00010028048 | S100A10 | -1.91165946 |
| ENSGALG00010007106 | SLC7A1 | -1.896838189 |
| ENSGALG00010017848 | CYP3A4 | -1.888926882 |
| ENSGALG00010009257 | - | -1.884398244 |
| ENSGALG00010001947 | TINAG | -1.884157745 |
| ENSGALG00010012303 | CA9 | -1.877406632 |
| ENSGALG00010004281 | VIM | -1.862533619 |
| ENSGALG00010001914 | - | -1.857305064 |
| ENSGALG00010019206 | RNF207 | -1.849197933 |
| ENSGALG00010018936 | SYT14 | -1.843622411 |
| ENSGALG00010011552 | - | -1.842826794 |
| ENSGALG00010024595 | MYBPHL | -1.835156767 |
| ENSGALG00010007936 | COL15A1 | -1.824723418 |
| ENSGALG00010025426 | SMARCD3 | -1.820263262 |
| ENSGALG00010005470 | SEMA3A | -1.819179563 |
| ENSGALG00010007069 | - | -1.818298341 |
| ENSGALG00010003920 | LPAR6 | -1.80386984 |
| ENSGALG00010017337 | ABAT | -1.802355658 |
| ENSGALG00010016306 | - | -1.800653752 |
| ENSGALG00010022117 | FAM20C | -1.791167979 |
| ENSGALG00010025538 | ANO1 | -1.791125692 |
| ENSGALG00010012282 | - | -1.780416238 |
| ENSGALG00010020700 | MTHFD2 | -1.779339778 |
| ENSGALG00010004907 | FRMD1 | -1.777420709 |
| novel285 | - | -1.775511336 |
| ENSGALG00010001206 | RSRC1 | -1.761672167 |
| novel680 | - | -1.757866507 |
| ENSGALG00010024716 | GTF2A2 | -1.751442555 |
| ENSGALG00010021556 | CAMK1 | -1.748327063 |
| ENSGALG00010012354 | DCBLD1 | -1.740151686 |
| ENSGALG00010000484 | - | -1.735219745 |
| novel709 | - | -1.734794398 |
| ENSGALG00010006202 | SACS | -1.720218402 |
| ENSGALG00010001901 | PRPS2 | -1.716088578 |
| ENSGALG00010023639 | KLHL23 | -1.698447129 |
| ENSGALG00010010991 | C2CD2 | -1.694576105 |
| ENSGALG00010016562 | GLIS2 | -1.688505517 |
| novel651 | - | -1.66780978 |
| ENSGALG00010029739 | P2RX5 | -1.662488031 |
| ENSGALG00010021378 | KIF11 | -1.655222069 |
| ENSGALG00010027364 | FSTL3 | -1.655147013 |

| | | |
|---|---|---|
| ENSGALG00010013104 | HLCS | -1.645123023 |
| ENSGALG00010014880 | SFRP2 | -1.643550904 |
| ENSGALG00010028025 | TNNC1 | -1.629642906 |
| ENSGALG00010009640 | NPR2 | -1.623149206 |
| ENSGALG00010001556 | EPB41L3 | -1.62102843 |
| ENSGALG00010014390 | SPARC | -1.620661214 |
| ENSGALG00010004929 | ACCSL | -1.615014229 |
| ENSGALG00010013930 | DDO | -1.60456196 |
| ENSGALG00010013207 | C1QTNF3 | -1.595382247 |
| ENSGALG00010005524 | STRIP2 | -1.593267262 |
| ENSGALG00010029424 | MAP2K6 | -1.588915981 |
| ENSGALG00010005990 | SLC2A12 | -1.588219838 |
| ENSGALG00010024871 | POLQ | -1.583070124 |
| ENSGALG00010008211 | ADGRB1 | -1.578950287 |
| ENSGALG00010004040 | LGALS1B | -1.575958732 |
| ENSGALG00010002102 | PAK1 | -1.575682299 |
| novel494 | - | -1.571562539 |
| ENSGALG00010021420 | PLXNB3 | -1.559452662 |
| novel597 | - | -1.554125651 |
| ENSGALG00010019451 | CAV3 | -1.551652425 |
| ENSGALG00010007162 | - | -1.548769571 |
| ENSGALG00010010358 | ASPM | -1.545066765 |
| ENSGALG00010013301 | PARVB | -1.542138127 |
| ENSGALG00010012879 | RCAN1 | -1.542051448 |
| ENSGALG00010005621 | CDH2 | -1.541577346 |
| ENSGALG00010008247 | FAM198B | -1.540962161 |
| ENSGALG00010002365 | RAI2 | -1.528639747 |
| ENSGALG00010019697 | EIF4EBP1 | -1.528021087 |
| ENSGALG00010022429 | ADK | -1.521692635 |
| novel384 | - | -1.503201395 |
| ENSGALG00010023362 | GADD45A | -1.500952439 |
| ENSGALG00010016466 | TMEM164 | -1.496262783 |
| ENSGALG00010024682 | TUBA1B | -1.493443919 |
| ENSGALG00010014031 | RPL22L1 | -1.493388392 |
| ENSGALG00010008455 | FKBP1B | -1.488157487 |
| ENSGALG00010013078 | COL5A2 | -1.485948887 |
| ENSGALG00010018149 | PITPNC1L | -1.484708723 |
| ENSGALG00010012876 | KDELR3 | -1.472716776 |
| ENSGALG00010021215 | SH3PXD2A | -1.46792419 |
| ENSGALG00010024709 | TEAD4 | -1.467194191 |
| ENSGALG00010006885 | RHOU | -1.463630662 |
| ENSGALG00010010528 | GPD2 | -1.463624026 |
| ENSGALG00010029943 | HS3ST3A1 | -1.462943406 |
| ENSGALG00010007994 | FAM65B | -1.452747579 |

| | | |
|---|---|---|
| ENSGALG00010011246 | DNAJB5 | -1.452372679 |
| ENSGALG00010013967 | MLLT3 | -1.452112069 |
| ENSGALG00010011926 | DYRK2 | -1.451514522 |
| ENSGALG00010028434 | COL5A1 | -1.447425227 |
| ENSGALG00010022925 | - | -1.44371222 |
| ENSGALG00010020193 | VSTM4 | -1.443567272 |
| ENSGALG00010023724 | CYP2R1 | -1.436238868 |
| ENSGALG00010025335 | - | -1.434567955 |
| ENSGALG00010008852 | KHDRBS3 | -1.433676939 |
| ENSGALG00010014162 | COL18A1 | -1.428649709 |
| ENSGALG00010020440 | PAX7 | -1.428242295 |
| ENSGALG00010015862 | PDGFRL | -1.426433087 |
| ENSGALG00010019418 | ZNF703 | -1.422848539 |
| ENSGALG00010010290 | - | -1.420851356 |
| novel403 | - | -1.41314693 |
| ENSGALG00010007232 | - | -1.40335764 |
| ENSGALG00010009469 | DNMT3A | -1.397200288 |
| ENSGALG00010002342 | BAG2 | -1.396201284 |
| ENSGALG00010011922 | PIK3R1 | -1.395185061 |
| ENSGALG00010020390 | KLHL25 | -1.394163935 |
| ENSGALG00010000313 | - | -1.388036993 |
| ENSGALG00010016519 | MEX3B | -1.386310514 |
| ENSGALG00010000595 | MYO10 | -1.383594639 |
| ENSGALG00010022515 | CHRNA1 | -1.382099264 |
| novel684 | - | -1.381558057 |
| ENSGALG00010000586 | - | -1.375681108 |
| ENSGALG00010013174 | ADAMTS12 | -1.372404105 |
| ENSGALG00010012264 | MYF5 | -1.371614297 |
| ENSGALG00010002554 | EGFL6 | -1.364816147 |
| ENSGALG00010011984 | FN1 | -1.350083971 |
| ENSGALG00010029618 | TEX2 | -1.349780988 |
| ENSGALG00010024676 | - | -1.348694965 |
| ENSGALG00010008291 | PTGFRN | -1.345041807 |
| ENSGALG00010021877 | PLEKHN1 | -1.33885095 |
| ENSGALG00010022900 | MMP17 | -1.336604791 |
| ENSGALG00010012614 | LHFPL2 | -1.335788784 |
| ENSGALG00010024744 | KLF13 | -1.334285077 |
| ENSGALG00010025357 | OLFML3 | -1.333966516 |
| ENSGALG00010008429 | IGFBP3 | -1.333642625 |
| ENSGALG00010018085 | PGP | -1.330639878 |
| ENSGALG00010016339 | SETD3 | -1.329915672 |
| ENSGALG00010028005 | IER5L | -1.329388041 |
| novel1 | - | -1.316220625 |
| ENSGALG00010026595 | PRKAA2 | -1.30974616 |

| | | |
|---|---|---|
| ENSGALG00010012869 | HTATIP2 | -1.307928507 |
| ENSGALG00010024401 | KCNC1 | -1.29279706 |
| ENSGALG00010004060 | ETV1 | -1.290685686 |
| ENSGALG00010018193 | ANK1 | -1.288383551 |
| ENSGALG00010014295 | CACNA1C | -1.286089781 |
| ENSGALG00010001152 | FEM1C | -1.283634939 |
| ENSGALG00010009135 | TENM4 | -1.280249729 |
| ENSGALG00010018747 | FLRT3 | -1.278199306 |
| ENSGALG00010000684 | COBL | -1.273279972 |
| ENSGALG00010008168 | MURC | -1.272215165 |
| ENSGALG00010012654 | FAM46B | -1.272008043 |
| ENSGALG00010027173 | AES | -1.266755387 |
| ENSGALG00010029386 | SLC16A6 | -1.265473307 |
| ENSGALG00010008688 | HBEGF | -1.263080935 |
| ENSGALG00010022881 | CCNB1 | -1.262639942 |
| ENSGALG00010016463 | FREM1 | -1.260491888 |
| ENSGALG00010022303 | DUSP13A | -1.258194223 |
| ENSGALG00010022828 | PRKG1 | -1.254580693 |
| ENSGALG00010025705 | ACSL3 | -1.252247936 |
| ENSGALG00010009477 | ZNF365 | -1.250579316 |
| ENSGALG00010018166 | MSS51 | -1.247420531 |
| ENSGALG00010016266 | PDLIM4 | -1.247215917 |
| ENSGALG00010027821 | NMRK2 | -1.241668746 |
| ENSGALG00010012599 | DMGDH | -1.234262351 |
| ENSGALG00010004534 | CUBN | -1.232279428 |
| ENSGALG00010022769 | FXYD6 | -1.226048264 |
| ENSGALG00010021867 | CXCL12 | -1.222102832 |
| ENSGALG00010027717 | ATXN7L2 | -1.221195224 |
| ENSGALG00010023533 | FKBP7 | -1.21554984 |
| ENSGALG00010025562 | GRAMD1B | -1.213962544 |
| ENSGALG00010028375 | LMNB2 | -1.20142277 |
| ENSGALG00010005437 | PDE7B | -1.19813243 |
| ENSGALG00010013012 | TMEM182 | -1.194866892 |
| ENSGALG00010022698 | SEMA6D | -1.189947141 |
| ENSGALG00010005068 | PLCXD2 | -1.186398192 |
| ENSGALG00010003372 | ENOX1 | -1.182846487 |
| ENSGALG00010003273 | NFIX | -1.18140652 |
| ENSGALG00010028076 | ST6GALNAC6 | -1.17948073 |
| ENSGALG00010027977 | BOLA1 | -1.177640917 |
| ENSGALG00010005288 | CNKSR3 | -1.173004485 |
| ENSGALG00010023620 | IRS1 | -1.170053062 |
| ENSGALG00010003201 | MBOAT2 | -1.169629009 |
| ENSGALG00010003860 | TMEM70 | -1.158502463 |
| ENSGALG00010027217 | SEMA6B | -1.157282556 |

| | | |
|---|---|---|
| ENSGALG00010025968 | FADS2 | -1.156441702 |
| ENSGALG00010001195 | ZNF608 | -1.149105486 |
| ENSGALG00010002923 | SERPINH1 | -1.147204516 |
| ENSGALG00010021121 | PLA2R1 | -1.144646503 |
| ENSGALG00010004365 | TPD52 | -1.137685208 |
| ENSGALG00010018672 | PKDCCA | -1.134754756 |
| ENSGALG00010017362 | ENO1 | -1.130585547 |
| ENSGALG00010027803 | SAPCD2 | -1.128699802 |
| ENSGALG00010027075 | SLC41A1 | -1.123940168 |
| ENSGALG00010028031 | NISCH | -1.122288455 |
| ENSGALG00010024234 | ARHGEF4 | -1.113621669 |
| ENSGALG00010023802 | SYNJ2BP | -1.112465289 |
| ENSGALG00010001826 | HOXA7 | -1.11060851 |
| ENSGALG00010024592 | COMT | -1.105318707 |
| ENSGALG00010005367 | H2AZ1 | -1.09943048 |
| ENSGALG00010026566 | - | -1.09770984 |
| ENSGALG00010024950 | NCAM1 | -1.096400436 |
| ENSGALG00010002235 | MXRA5 | -1.091788173 |
| ENSGALG00010026115 | MMACHC | -1.091533685 |
| ENSGALG00010025342 | TCEANC2 | -1.087799766 |
| ENSGALG00010012015 | - | -1.08690369 |
| ENSGALG00010001840 | HOXA9 | -1.085866693 |
| ENSGALG00010011913 | CXXC5 | -1.082648123 |
| ENSGALG00010013356 | AMER1 | -1.082387297 |
| ENSGALG00010009724 | ISOC1 | -1.075603279 |
| ENSGALG00010017878 | CHRNG | -1.073921041 |
| ENSGALG00010012962 | SLC25A17 | -1.069685817 |
| ENSGALG00010012885 | PMS1 | -1.064347242 |
| ENSGALG00010025442 | ABCF2 | -1.060645489 |
| ENSGALG00010003806 | EGF | -1.056446009 |
| ENSGALG00010025418 | CASQ2 | -1.055183906 |
| ENSGALG00010017332 | FGFR1 | -1.04330527 |
| ENSGALG00010027201 | BGN | -1.04025391 |
| ENSGALG00010004622 | ZNF628 | -1.036545723 |
| ENSGALG00010025032 | FSTL1 | -1.026766301 |
| ENSGALG00010024823 | ASB10 | -1.026590028 |
| ENSGALG00010002134 | ANKRD28 | -1.024665591 |
| ENSGALG00010021714 | TSPAN18 | -1.023983697 |
| ENSGALG00010008146 | SOX4 | -1.021886411 |
| ENSGALG00010024735 | OTUD7A | -1.021672313 |
| ENSGALG00010028447 | OLFM1 | -1.021063211 |
| ENSGALG00010024732 | AHCY | -1.016198392 |
| ENSGALG00010024402 | GPC1 | -1.013816869 |
| ENSGALG00010023668 | RCN1 | -1.0083106 |

| | | |
|---|---|---|
| ENSGALG00010005255 | BZW2 | -1.00434993 |
| ENSGALG00010012194 | LRRFIP1 | -1.003981995 |
| ENSGALG00010018901 | TCF7L2 | -1.000861627 |
| ENSGALG00010017730 | TARP | 1.008882604 |
| ENSGALG00010018943 | ACKR4 | 1.433148225 |
| ENSGALG00010012726 | IL7R | 1.522562594 |
| ENSGALG00010014416 | H2BC32 | 1.60477039 |
| ENSGALG00010002768 | TRIM39.2 | 2.395933229 |
| ENSGALG00010021102 | GOLGA3 | 1.00972794088477 |
| ENSGALG00010019158 | - | 1.0116832681121999 |
| ENSGALG00010009202 | FABP6 | 1.0124722439051599 |
| ENSGALG00010028046 | SMIM4 | 1.0140926179128 |
| ENSGALG00010019576 | - | 1.01426465913583 |
| ENSGALG00010020509 | CRBN | 1.01462734565365 |
| ENSGALG00010015852 | ZFAND5 | 1.02905066843072 |
| ENSGALG00010016880 | AR | 1.03345594462979 |
| ENSGALG00010025685 | NCKAP1L | 1.03386206335275 |
| ENSGALG00010013044 | NOS1AP | 1.03805376880365 |
| ENSGALG00010015544 | RTCB | 1.03883594595532 |
| ENSGALG00010019213 | RHBDF1 | 1.04092160638473 |
| ENSGALG00010013355 | FAM171B | 1.0433710498888 |
| ENSGALG00010028116 | HYAL1 | 1.04639731740059 |
| ENSGALG00010002556 | FGL1B | 1.04661198533664 |
| ENSGALG00010010431 | SOCS2 | 1.04661819524156 |
| ENSGALG00010002614 | GPAT3 | 1.04889092933499 |
| ENSGALG00010019267 | STARD5 | 1.05024216407152 |
| ENSGALG00010007786 | SAMSN1 | 1.0521389331763 |
| ENSGALG00010021953 | PITPNM1 | 1.05694933053443 |
| ENSGALG00010024516 | EHHADH | 1.06071468957632 |
| ENSGALG00010008395 | ENDOD1 | 1.06098531333787 |
| ENSGALG00010023740 | PPP1R16B | 1.0610558154382401 |
| ENSGALG00010020282 | MAGI1 | 1.06605646528596 |
| ENSGALG00010005249 | PDGFD | 1.0682600783405 |
| ENSGALG00010013706 | - | 1.07608160630054 |
| ENSGALG00010025210 | HSPB2 | 1.08720285222139 |
| ENSGALG00010012352 | PTPRC | 1.09511683448276 |
| ENSGALG00010010742 | LCP2 | 1.1020012633086 |
| ENSGALG00010014399 | GAS6 | 1.1026400593007402 |
| ENSGALG00010018404 | IL2RG | 1.10677946809303 |
| ENSGALG00010001606 | PIGA | 1.1088062995850199 |
| ENSGALG00010008316 | ADGRF5 | 1.11190572101288 |
| ENSGALG00010008453 | AMOTL1 | 1.11278613161872 |
| ENSGALG00010000722 | ANKH | 1.1145932694765701 |
| ENSGALG00010017207 | - | 1.1159240714195902 |

| | | |
|---|---|---|
| ENSGALG00010009373 | ARHGAP15 | 1.11862607607212 |
| ENSGALG00010027081 | PDE4B | 1.12256380048749 |
| ENSGALG00010021645 | ASCC1 | 1.12810038752861 |
| ENSGALG00010007653 | - | 1.1300894096350902 |
| ENSGALG00010020557 | - | 1.13280280738054 |
| ENSGALG00010009163 | SAMD12 | 1.13409626179929 |
| ENSGALG00010002999 | CMTM7 | 1.1391330903605201 |
| ENSGALG00010023891 | MYLKSML | 1.1407280120001202 |
| ENSGALG00010015017 | FAM184A | 1.14960265930274 |
| ENSGALG00010001545 | SELENOO | 1.15265238182023 |
| ENSGALG00010005042 | SLC46A3 | 1.15599661685976 |
| ENSGALG00010011988 | HINTW | 1.1584843640371298 |
| ENSGALG00010002405 | KCNQ5 | 1.1596656399302299 |
| ENSGALG00010024897 | ADAL | 1.16559287185355 |
| ENSGALG00010022081 | FAS | 1.16593124959302 |
| ENSGALG00010029315 | TBC1D16 | 1.16660432872757 |
| ENSGALG00010024074 | ADCY7 | 1.1697972577384799 |
| ENSGALG00010024881 | CASP10 | 1.1721071641369498 |
| ENSGALG00010013264 | DNAJC21 | 1.1775993572682901 |
| ENSGALG00010016281 | SLC22A4 | 1.17996335482815 |
| ENSGALG00010023819 | BIN2 | 1.18336395512973 |
| ENSGALG00010013898 | EMB | 1.18482821337622 |
| ENSGALG00010012917 | RUNX1 | 1.19596661416256 |
| ENSGALG00010028918 | AGPAT2 | 1.2035644376744699 |
| ENSGALG00010027619 | JAK3 | 1.2037061762407801 |
| ENSGALG00010010923 | F13A1 | 1.2063390890181298 |
| ENSGALG00010025979 | - | 1.20677398161673 |
| ENSGALG00010016874 | ADAMTSL3 | 1.20880182179637 |
| ENSGALG00010009307 | DIRAS2 | 1.2090296969049699 |
| ENSGALG00010005656 | CYP2U1 | 1.20939595498019 |
| ENSGALG00010029997 | - | 1.21656778956893 |
| ENSGALG00010008142 | CD247 | 1.22137140334873 |
| ENSGALG00010014705 | ARAP2 | 1.2222841219711698 |
| ENSGALG00010009524 | FAM185A | 1.22524746836428 |
| ENSGALG00010016987 | ASB2 | 1.22919807354786 |
| ENSGALG00010009193 | EDARADD | 1.2319576980031102 |
| ENSGALG00010025623 | RAB44 | 1.23755783696634 |
| ENSGALG00010007768 | - | 1.24332791015253 |
| ENSGALG00010024975 | - | 1.24840314110756 |
| ENSGALG00010010970 | TPM2 | 1.25478731758107 |
| ENSGALG00010003437 | PNRC1 | 1.25533350155513 |
| ENSGALG00010029198 | PPM1D | 1.25560096330911 |
| ENSGALG00010005386 | CPOX | 1.25670283709934 |
| ENSGALG00010024675 | NR1D1 | 1.26096582602982 |

| | | |
|---|---|---|
| ENSGALG00010026062 | ITGA4 | 1.26169987891934 |
| ENSGALG00010025195 | P2RX7 | 1.26178193049875 |
| ENSGALG00010008960 | LRRC2 | 1.26232312785444 |
| ENSGALG00010008405 | FAM84B | 1.27561665074367 |
| ENSGALG00010029417 | ABCA9 | 1.27946393561874 |
| ENSGALG00010003439 | GMIP | 1.28366819084178 |
| ENSGALG00010024717 | TF | 1.28627750829749 |
| ENSGALG00010003164 | C3 | 1.28865019307317 |
| ENSGALG00010020809 | PITX3 | 1.29055174155085 |
| ENSGALG00010008392 | CD36 | 1.29220562949348 |
| ENSGALG00010021370 | - | 1.30143762324558 |
| ENSGALG00010025580 | RASSF7 | 1.30312987565322 |
| ENSGALG00010003895 | THSD7A | 1.30979328572181 |
| ENSGALG00010011932 | VAV3 | 1.3121120472768402 |
| ENSGALG00010027244 | ARHGEF18 | 1.32372999469288 |
| ENSGALG00010001320 | CPED1 | 1.3268841265723799 |
| ENSGALG00010018822 | TRAF5 | 1.33270759146904 |
| ENSGALG00010024655 | IGF2BP2 | 1.3417027140581699 |
| ENSGALG00010007781 | MBP | 1.3446749859717602 |
| ENSGALG00010029341 | SLC43A2 | 1.3460312936773797 |
| ENSGALG00010029158 | CD7 | 1.34650535086763 |
| ENSGALG00010007007 | ACSL1 | 1.34843772017034 |
| ENSGALG00010016978 | FAM181A | 1.34878227848907 |
| ENSGALG00010009199 | SEMA4D | 1.3493182219057598 |
| ENSGALG00010026846 | MICAL1 | 1.35311208547008 |
| ENSGALG00010023221 | AMY1A | 1.3556507276499798 |
| ENSGALG00010014573 | CEBPB | 1.35813627060599 |
| ENSGALG00010027040 | CLIP1 | 1.35892222769292 |
| ENSGALG00010013859 | MYOC | 1.3598325018586201 |
| ENSGALG00010019271 | CDKL1 | 1.36011645354007 |
| ENSGALG00010027911 | PTPN22 | 1.36397930877381 |
| ENSGALG00010024885 | SLC4A1 | 1.36513789737187 |
| ENSGALG00010022251 | WNK4 | 1.3667307935208202 |
| ENSGALG00010012106 | TMTC1 | 1.3703810758559498 |
| ENSGALG00010022717 | ELOVL1 | 1.37398091265037 |
| ENSGALG00010023397 | - | 1.3843899647293898 |
| ENSGALG00010005521 | SYTL3 | 1.3885512370475102 |
| ENSGALG00010010779 | DOCK2 | 1.39182298925917 |
| ENSGALG00010025331 | - | 1.39702010576146 |
| ENSGALG00010017004 | CX3CR1 | 1.3985010611020499 |
| ENSGALG00010002520 | TTC32 | 1.40421041898093 |
| ENSGALG00010016404 | ESRRG | 1.4057488957090403 |
| ENSGALG00010029255 | RAP1GAP2 | 1.41698912178391 |
| ENSGALG00010027110 | IKBKE | 1.41945237427569 |

| | | |
|---|---|---|
| ENSGALG00010011573 | CMAS | 1.42020859286524 |
| ENSGALG00010009730 | C2H8ORF76 | 1.42062923726928 |
| ENSGALG00010012248 | H2AC39 | 1.42623296025902 |
| ENSGALG00010013899 | WNT5B | 1.42867160967709 |
| ENSGALG00010009253 | SLC45A4 | 1.4300098977806002 |
| ENSGALG00010000674 | GRB10 | 1.43140746686856 |
| ENSGALG00010026183 | SESTD1 | 1.43211975123106 |
| ENSGALG00010003815 | RASAL3 | 1.4358737873716598 |
| ENSGALG00010012529 | BEND3 | 1.43616103328308 |
| ENSGALG00010001385 | MME | 1.4380810194663 |
| ENSGALG00010021617 | SORBS1 | 1.4383315163495298 |
| ENSGALG00010013954 | LPL | 1.43861266570792 |
| ENSGALG00010023743 | CD3D | 1.4405697451391 |
| ENSGALG00010002914 | SLC27A1 | 1.4424666416838101 |
| ENSGALG00010017706 | FGFRL1 | 1.44654517395646 |
| ENSGALG00010023715 | CD3E | 1.45397333044182 |
| ENSGALG00010017441 | SPSB1 | 1.45619618336238 |
| ENSGALG00010029722 | SPECC1 | 1.46382951145234 |
| ENSGALG00010013958 | TUSC3 | 1.4645577597957098 |
| ENSGALG00010018758 | TNFRSF9 | 1.4645730111265303 |
| ENSGALG00010013172 | TNFSF13B | 1.47545310815251 |
| ENSGALG00010028550 | ZAP70 | 1.4760303057014001 |
| ENSGALG00010008101 | CENPQ | 1.4803751486986798 |
| ENSGALG00010013213 | SH2D1A | 1.4835507696555599 |
| ENSGALG00010015196 | PDE4D | 1.48646113050057 |
| ENSGALG00010006843 | STON2 | 1.4886729199462199 |
| ENSGALG00010019282 | IGFBP2 | 1.4918800626497501 |
| novel100 | - | 1.4959991884262902 |
| ENSGALG00010024835 | FAM189A1 | 1.4985034367519199 |
| ENSGALG00010011636 | GRIK1 | 1.5050186646511998 |
| ENSGALG00010017849 | FBXO48 | 1.5094210078018897 |
| ENSGALG00010025044 | TMEM9 | 1.51039399733947 |
| ENSGALG00010011989 | H1-010 | 1.51206529898979 |
| ENSGALG00010014929 | FHL1 | 1.5240782593368198 |
| ENSGALG00010000576 | NOS1 | 1.52528418119152 |
| novel411 | - | 1.5345291426328 |
| ENSGALG00010008509 | MGARP | 1.5346364507584798 |
| ENSGALG00010019318 | NOG2 | 1.53528697241795 |
| ENSGALG00010009166 | CLGN | 1.5434482767967002 |
| ENSGALG00010011738 | FGD6 | 1.5439412180583798 |
| ENSGALG00010028419 | GBP4L | 1.5478580419122099 |
| ENSGALG00010024254 | - | 1.5482779662789 |
| ENSGALG00010016676 | NLRC3 | 1.55014536615354 |
| ENSGALG00010011313 | RASSF9 | 1.56344688774776 |

| | | |
|---|---|---|
| ENSGALG00010011139 | UBASH3A | 1.56451642834308 |
| ENSGALG00010021634 | PLCD4 | 1.56985275986924 |
| ENSGALG00010023331 | CD4 | 1.56998151888652 |
| ENSGALG00010028450 | PTGDS | 1.5716628856602401 |
| novel266 | - | 1.5746219512738702 |
| ENSGALG00010002136 | ACAP1 | 1.58058218330641 |
| ENSGALG00010027209 | TRAF3IP3 | 1.59064474225067 |
| ENSGALG00010019050 | TNS1 | 1.5951972674261499 |
| ENSGALG00010013147 | - | 1.5957185855434 |
| ENSGALG00010000318 | - | 1.59857276570752 |
| ENSGALG00010024142 | DNAJC6 | 1.6098764658670903 |
| ENSGALG00010015339 | - | 1.61068345421592 |
| ENSGALG00010000687 | IKZF1 | 1.61154566314425 |
| ENSGALG00010003820 | SH3YL1 | 1.61374581889592 |
| ENSGALG00010012145 | - | 1.61449814386013 |
| ENSGALG00010016963 | CD8A | 1.62275739741775 |
| ENSGALG00010006943 | - | 1.62377668940718 |
| ENSGALG00010020106 | TMEM52 | 1.6336167686546001 |
| ENSGALG00010003364 | ANKRD6 | 1.67150154767704 |
| ENSGALG00010016456 | LAMP3 | 1.6821876943070802 |
| ENSGALG00010023441 | C15orf52 | 1.69333685459279 |
| novel404 | - | 1.69787822538522 |
| novel681 | - | 1.69869762313959 |
| ENSGALG00010018384 | LAMP5 | 1.7113110695403702 |
| ENSGALG00010017030 | - | 1.71430958778097 |
| ENSGALG00010010586 | CYTIP | 1.7243962616914499 |
| ENSGALG00010017439 | PPP4R4 | 1.7300327675173799 |
| ENSGALG00010011278 | TDH | 1.7311430079758603 |
| ENSGALG00010018013 | - | 1.7380852817131698 |
| novel390 | - | 1.74925515948989 |
| ENSGALG00010012307 | - | 1.7508628324286202 |
| ENSGALG00010029403 | FAM20A | 1.76426722967939 |
| novel524 | - | 1.7673148147472502 |
| ENSGALG00010017747 | DUSP26 | 1.7787470298839299 |
| ENSGALG00010017668 | INPPL1 | 1.7860740395718 |
| ENSGALG00010007678 | CDH8 | 1.7881602749164098 |
| ENSGALG00010029409 | ABCA8 | 1.7943539293540298 |
| ENSGALG00010013098 | - | 1.7979349087381002 |
| ENSGALG00010013309 | CGTL | 1.80301181573397 |
| ENSGALG00010009777 | ST3GAL1 | 1.8117385901082 |
| ENSGALG00010019027 | GNLY | 1.81402802468644 |
| ENSGALG00010021191 | C5H11ORF9 | 1.81507971231722 |
| ENSGALG00010004602 | TCTE3 | 1.83371446575198 |
| ENSGALG00010021711 | - | 1.8352091037480802 |

| | | |
|---|---|---|
| ENSGALG00010027314 | PDE4C | 1.84199370943629 |
| ENSGALG00010002473 | - | 1.84466222024587 |
| ENSGALG00010016274 | ZNF831 | 1.8454772136024198 |
| ENSGALG00010023204 | SRGN | 1.8476969682981101 |
| ENSGALG00010011461 | IRF4 | 1.84954795448836 |
| ENSGALG00010025653 | IGSF11 | 1.8587636685188502 |
| ENSGALG00010012582 | CD24 | 1.86061153845209 |
| ENSGALG00010017475 | STUM | 1.86504559932105 |
| ENSGALG00010018421 | - | 1.8653724757431 |
| ENSGALG00010002557 | - | 1.8658419321956299 |
| ENSGALG00010029427 | SPNS3 | 1.8687437021612099 |
| novel152 | - | 1.8783438302284199 |
| ENSGALG00010010791 | H-10 | 1.87997086220584 |
| ENSGALG00010023251 | TRBV6-5 | 1.8846407625398303 |
| ENSGALG00010016928 | RGS11 | 1.88883328718952 |
| ENSGALG00010007388 | CYP1B1 | 1.8910444131683002 |
| ENSGALG00010025890 | SCG2 | 1.89391349121522 |
| ENSGALG00010009036 | - | 1.90005787745538 |
| ENSGALG00010024567 | IKZF3 | 1.9016057995583 |
| ENSGALG00010028016 | PRF1 | 1.9223488021654698 |
| ENSGALG00010017999 | GPR174 | 1.92248086054894 |
| ENSGALG00010007769 | - | 1.92291218722167 |
| ENSGALG00010002605 | EOMES | 1.92425540069141 |
| ENSGALG00010029947 | CCL5 | 1.9285705445468602 |
| novel535 | - | 1.94252252693273 |
| ENSGALG00010011886 | NTNG1 | 1.95032558131818 |
| ENSGALG00010024421 | DAPK2 | 1.95158454262638 |
| ENSGALG00010014000 | STAT4 | 1.9563551604052498 |
| ENSGALG00010008100 | - | 1.9611075674471 |
| ENSGALG00010006277 | GNG10 | 1.9639400612203899 |
| ENSGALG00010009972 | PTK2B | 1.9668164066859901 |
| ENSGALG00010008676 | - | 1.97272751719253 |
| ENSGALG00010015076 | ITK | 1.9836578368661397 |
| ENSGALG00010011917 | HSF2BP | 1.99593234034464 |
| ENSGALG00010016619 | SLC25A47 | 2.00199887146224 |
| ENSGALG00010002414 | MHCY4 | 2.00364161626708 |
| ENSGALG00010006816 | - | 2.01106957086642 |
| ENSGALG00010025985 | CHCHD10 | 2.01167211007029 |
| ENSGALG00010013194 | GRAP2 | 2.0136512049523403 |
| ENSGALG00010021157 | - | 2.02442518959938 |
| ENSGALG00010005510 | MYOZ2 | 2.0269046692680703 |
| ENSGALG00010020212 | JMJD7 | 2.03255806158553 |
| ENSGALG00010012191 | BEST3 | 2.03402883944135 |
| ENSGALG00010010638 | ACVR1C | 2.05468117985098 |

| | | |
|---|---|---|
| ENSGALG00010016287 | BCL11B | 2.05864692690141 |
| novel642 | - | 2.06724852471092 |
| ENSGALG00010024433 | GPC2 | 2.07543804495689 |
| ENSGALG00010023158 | - | 2.07603522444707 |
| ENSGALG00010025305 | GPR182 | 2.07644046802438 |
| ENSGALG00010022343 | GATA5 | 2.08329285809654 |
| ENSGALG00010013419 | - | 2.09021793845374 |
| ENSGALG00010006681 | CRISPLD2 | 2.09101560496746 |
| ENSGALG00010014760 | - | 2.10160939729052 |
| ENSGALG00010002866 | BTN1 | 2.1045263988408403 |
| ENSGALG00010016599 | - | 2.1074376449351298 |
| ENSGALG00010021171 | - | 2.1435192781261 |
| novel207 | - | 2.15259567727415 |
| ENSGALG00010008275 | CELSR1 | 2.15885690562312 |
| ENSGALG00010022873 | NGEF | 2.16573823619698 |
| ENSGALG00010016328 | USH2A | 2.1721913367147 |
| ENSGALG00010018877 | CST7 | 2.17746024482573 |
| ENSGALG00010012133 | RAB3IP | 2.19493041560627 |
| ENSGALG00010025042 | BCO2 | 2.19612211103724 |
| ENSGALG00010005384 | MAPRE2 | 2.1996752550901 |
| ENSGALG00010027034 | - | 2.21453187244962 |
| ENSGALG00010002403 | ZNFY3 | 2.2300252463500496 |
| ENSGALG00010022886 | PLA2G15 | 2.2339124241422503 |
| ENSGALG00010022619 | MADD | 2.2359996219590497 |
| ENSGALG00010022267 | - | 2.2679001166712798 |
| ENSGALG00010028517 | RNF208 | 2.28029337502872 |
| ENSGALG00010008824 | GREM2 | 2.28678083893452 |
| ENSGALG00010027212 | IRF6 | 2.2971256453763997 |
| ENSGALG00010017285 | IL21R | 2.3183176029123103 |
| ENSGALG00010020087 | MUC13 | 2.34671346825246 |
| ENSGALG00010004924 | TMPRSS11F | 2.3507160480041405 |
| ENSGALG00010017053 | - | 2.35535814157452 |
| ENSGALG00010024305 | - | 2.37372501353509 |
| ENSGALG00010027232 | ARRDC2 | 2.3767613879430898 |
| ENSGALG00010009776 | - | 2.39036810120893 |
| ENSGALG00010024340 | NETO2 | 2.3915391287319703 |
| ENSGALG00010022231 | ZNF503 | 2.4111408194568797 |
| ENSGALG00010023621 | CD79B | 2.4143297910471 |
| ENSGALG00010003869 | LY96 | 2.4341384399760098 |
| ENSGALG00010004300 | VNN2 | 2.4514456056691802 |
| ENSGALG00010005067 | - | 2.4601307386318703 |
| ENSGALG00010018270 | - | 2.4831607976664998 |
| ENSGALG00010014375 | H2BC35 | 2.49112306929048 |
| ENSGALG00010004866 | SLC25A21 | 2.49212684660875 |

| | | |
|---|---|---|
| ENSGALG00010001708 | AADAC | 2.50096639122247 |
| ENSGALG00010009021 | HNF4beta | 2.50529592017621 |
| ENSGALG00010015105 | GZMK | 2.50960897605314 |
| ENSGALG00010002784 | TRIM27.2 | 2.5168494632968397 |
| ENSGALG00010025905 | CPT1A | 2.55863157353058 |
| ENSGALG00010005005 | - | 2.55985609434698 |
| ENSGALG00010015732 | - | 2.56286180316587 |
| ENSGALG00010019875 | PLEKHH1 | 2.56751436443906 |
| ENSGALG00010012324 | - | 2.5683934776967 |
| ENSGALG00010006608 | KL | 2.5780397973109 |
| ENSGALG00010024958 | - | 2.58595949647159 |
| ENSGALG00010018311 | NKX2-3 | 2.5873457555290296 |
| ENSGALG00010022088 | DKK3 | 2.6552520076196604 |
| novel114 | - | 2.67155363412117 |
| ENSGALG00010026016 | SLC2A11 | 2.68037340613454 |
| ENSGALG00010012371 | ATP6V1G3 | 2.69456521202981 |
| ENSGALG00010005238 | TMEM63C | 2.69756902452847 |
| ENSGALG00010008074 | RHAG | 2.69851621552441 |
| novel703 | - | 2.71309975666224 |
| ENSGALG00010024226 | POU2AF1 | 2.7257130632156605 |
| ENSGALG00010005609 | GJB6 | 2.7444124118088196 |
| novel528 | - | 2.74556098307945 |
| novel83 | - | 2.7457869283864804 |
| ENSGALG00010004375 | - | 2.74725721799787 |
| ENSGALG00010001111 | SUCNR1 | 2.75319218338286 |
| ENSGALG00010003641 | - | 2.7561730032820297 |
| ENSGALG00010019253 | HBAD | 2.7654968341255097 |
| ENSGALG00010000358 | - | 2.79818472564484 |
| ENSGALG00010015673 | - | 2.80314518181233 |
| ENSGALG00010002819 | BMP3 | 2.8179574503419196 |
| ENSGALG00010002810 | TRIM27.1 | 2.8548441586594904 |
| ENSGALG00010016165 | KK34 | 2.8593859744992405 |
| ENSGALG00010008245 | FBXO32 | 2.87330336415716 |
| ENSGALG00010015090 | GZMA | 2.8900899581943897 |
| ENSGALG00010013322 | IRS2 | 2.9060294495117795 |
| ENSGALG00010003756 | MHCY7 | 2.92916884322002 |
| ENSGALG00010022482 | PRTG | 2.9399244378756295 |
| ENSGALG00010023485 | BARX2 | 2.9620391102879102 |
| ENSGALG00010004692 | - | 3.03679738977379 |
| ENSGALG00010007181 | GJB2 | 3.0386537626516 |
| ENSGALG00010004158 | JCHAIN | 3.04763883812955 |
| ENSGALG00010022965 | - | 3.08892077050173 |
| novel595 | - | 3.11011992834404 |
| ENSGALG00010003192 | RGCC | 3.11633012265095 |

| | | |
|---|---|---|
| ENSGALG00010006618 | - | 3.1375681917743794 |
| ENSGALG00010024056 | - | 3.14767732135903 |
| ENSGALG00010015138 | - | 3.2448444344838703 |
| ENSGALG00010026238 | - | 3.2686634641761603 |
| ENSGALG00010024021 | IGLL1 | 3.2720574328839507 |
| ENSGALG00010004872 | KIAA0408 | 3.30389443676784 |
| ENSGALG00010021611 | GREM1 | 3.3896128778376298 |
| ENSGALG00010018190 | - | 3.41442682661087 |
| ENSGALG00010022309 | WSCD2 | 3.41581909592709 |
| ENSGALG00010004665 | - | 3.4435895293184 |
| novel311 | - | 3.4630133615439505 |
| novel523 | - | 3.47086800209266 |
| ENSGALG00010007638 | - | 3.52465200400709 |
| novel8 | - | 3.53751629331438 |
| ENSGALG00010003074 | TTPA | 3.55296728898044 |
| ENSGALG00010023559 | DFNB59 | 3.55755508870291 |
| ENSGALG00010025664 | GPR55 | 3.61585042276672 |
| ENSGALG00010002765 | MAP7D2 | 3.63933935467026 |
| novel103 | - | 3.6572727860180803 |
| ENSGALG00010003777 | - | 3.6843728116417402 |
| novel604 | - | 3.69483056489258 |
| ENSGALG00010024089 | - | 3.69878851463884 |
| ENSGALG00010024462 | ZBTB32 | 3.71780149503914 |
| ENSGALG00010002274 | - | 3.72829306969761 |
| novel521 | - | 3.7693051479057504 |
| novel332 | - | 3.82460160963036 |
| ENSGALG00010005333 | PLXNA4 | 3.85343624593442 |
| ENSGALG00010027120 | UNC13A | 3.88202032004107 |
| ENSGALG00010016602 | HSPB7 | 3.8908277462017 |
| ENSGALG00010024069 | - | 3.89934291602823 |
| ENSGALG00010008277 | MZB1 | 4.10038130703241 |
| novel214 | - | 4.12655787343819 |
| ENSGALG00010013296 | AGXT2 | 4.25078792791627 |
| ENSGALG00010017718 | ATOH8 | 4.31271526980088 |
| ENSGALG00010018689 | - | 4.43004168483604 |
| ENSGALG00010017320 | - | 4.46259622827515 |
| ENSGALG00010029345 | RILP | 4.53656456316516 |
| ENSGALG00010020417 | ZP1 | 4.59002339298463 |
| ENSGALG00010015132 | GCNT1 | 4.62416111658246 |
| ENSGALG00010013351 | EDAR | 4.63117251490541 |
| ENSGALG00010014889 | PVALB | 4.6608702032660405 |
| ENSGALG00010011357 | UNC80 | 4.69125289189045 |
| ENSGALG00010015107 | MB | 4.69511827629056 |
| ENSGALG00010015802 | TRPC7 | 4.70585925835267 |

| | | |
|---|---|---|
| novel341 | - | 4.7899933199824405 |
| ENSGALG00010002423 | - | 4.7919078497020005 |
| novel339 | - | 4.8933098610446795 |
| ENSGALG00010002122 | - | 4.92097701775432 |
| ENSGALG00010002436 | MHCY4 | 5.0455865228190495 |
| ENSGALG00010018687 | - | 5.18978745534266 |
| ENSGALG00010015160 | SIM1 | 5.39451434976591 |
| ENSGALG00010019493 | - | 5.48496576788684 |
| novel577 | - | 5.54243614354927 |
| novel154 | - | 5.55283701621976 |
| novel677 | - | 5.59987989022248 |
| ENSGALG00010018467 | TLX1 | 5.65484818295703 |
| ENSGALG00010003707 | - | 5.705984729518451 |
| ENSGALG00010024499 | CA12 | 5.779527922418691 |
| ENSGALG00010003079 | SP8 | 5.88717526297992 |
| ENSGALG00010024838 | EN2 | 5.892398088071049 |
| novel590 | - | 5.928009281144639 |
| novel223 | - | 5.968510920701919 |
| ENSGALG00010016770 | - | 6.22048958414577 |
| ENSGALG00010023302 | - | 6.43044271073269 |
| ENSGALG00010024453 | HTR3A | 6.48806354904858 |
| ENSGALG00010011974 | - | 6.512896373612031 |
| ENSGALG00010003749 | MHCY15 | 6.558449674361779 |
| ENSGALG00010011558 | MYBPC1 | 6.60460927804044 |
| novel222 | - | 7.04537841834793 |
| ENSGALG00010009067 | - | 7.15421495136927 |
| novel330 | - | 7.32428222521913 |
| novel326 | - | 8.731769538812651 |
| ENSGALG00010018616 | - | 9.310715113426891 |

Table S5.Enriched GO terms for DEGs identified between the LX and NX comparison group.

| GO ID | Source | Term | Pvalue | Number |
|---|---|---|---|---|
| GO:0005576 | CC | extracellular region | 1.68879558978088e-09 | 101 |
| GO:0071944 | CC | cell periphery | 2.63079229503245e-08 | 191 |
| GO:0005615 | CC | extracellular space | 1.93045024865663e-07 | 75 |
| GO:0009897 | CC | external side of plasma membrane | 1.93337375798202e-07 | 28 |
| GO:0009986 | CC | cell surface | 3.06516017723073e-07 | 39 |
| GO:0042612 | CC | MHC class I protein complex | 3.74561329036568e-07 | 9 |
| GO:0098553 | CC | lumenal side of endoplasmic reticulum membrane | 3.74561329036568e-07 | 9 |
| GO:0098576 | CC | lumenal side of membrane | 3.74561329036568e-07 | 9 |
| GO:0098552 | CC | side of membrane | 3.89589614352956e-07 | 36 |
| GO:0062023 | CC | collagen-containing extracellular matrix | 6.50448897392735e-07 | 25 |
| GO:0030312 | CC | external encapsulating structure | 5.69441384601735e-06 | 32 |
| GO:0031012 | CC | extracellular matrix | 5.69441384601735e-06 | 32 |
| GO:0042611 | CC | MHC protein complex | 7.77960795777618e-06 | 9 |
| GO:0005886 | CC | plasma membrane | 1.8847676072818e-05 | 160 |
| GO:0030670 | CC | phagocytic vesicle membrane | 3.38574296744263e-05 | 9 |
| GO:0030666 | CC | endocytic vesicle membrane | 0.0001342053851455 | 9 |
| GO:0098644 | CC | complex of collagen trimers | 0.000213850924632 | 4 |
| GO:0005583 | CC | fibrillar collagen trimer | 0.0003149370173979 | 3 |
| GO:0042105 | CC | alpha-beta T cell receptor complex | 0.0003149370173979 | 3 |
| GO:0098643 | CC | banded collagen fibril | 0.0003149370173979 | 3 |
| GO:0098797 | CC | plasma membrane protein complex | 0.000341487002173 | 31 |
| GO:0042101 | CC | T cell receptor complex | 0.0007618930438472 | 3 |
| GO:0005581 | CC | collagen trimer | 0.001316930563105 | 6 |
| GO:0098802 | CC | plasma membrane signaling receptor complex | 0.0015846084688366 | 11 |
| GO:0030017 | CC | sarcomere | 0.0017363080214402 | 11 |
| GO:0005584 | CC | collagen type I trimer | 0.0018808483322223 | 2 |
| GO:0071753 | CC | IgM immunoglobulin complex | 0.0018808483322223 | 2 |
| GO:0045211 | CC | postsynaptic membrane | 0.0018989976528633 | 14 |
| GO:0031252 | CC | cell leading edge | 0.003044471602398 | 15 |
| GO:0016020 | CC | membrane | 0.0031956060625438 | 300 |
| GO:0030424 | CC | axon | 0.0035759919912932 | 21 |
| GO:0150034 | CC | distal axon | 0.0036727200750718 | 10 |
| GO:0045335 | CC | phagocytic vesicle | 0.004189789365109 | 9 |
| GO:0030016 | CC | myofibril | 0.0046653507424556 | 11 |
| GO:0005884 | CC | actin filament | 0.0051405118425524 | 8 |
| GO:0014802 | CC | terminal cisterna | 0.0054798047305704 | 2 |
| GO:0002116 | CC | semaphorin receptor complex | 0.0056158474241162 | 3 |
| GO:0034702 | CC | ion channel complex | 0.0059027955926796 | 18 |

| | | | | |
|---|---|---|---|---|
| GO:0043292 | CC | contractile fiber | 0.0062357972889455 | 11 |
| GO:0000786 | CC | nucleosome | 0.0071099808165217 | 6 |
| GO:0030018 | CC | Z disc | 0.0076228604180497 | 7 |
| GO:0030426 | CC | growth cone | 0.008030735544866 | 6 |
| GO:0042383 | CC | sarcolemma | 0.0093183902161249 | 7 |
| GO:0043235 | CC | receptor complex | 0.0096771240915639 | 19 |
| GO:0044295 | CC | axonal growth cone | 0.0103380085255781 | 3 |
| GO:0031674 | CC | I band | 0.0112744902513725 | 7 |
| GO:0097060 | CC | synaptic membrane | 0.0112767239292831 | 15 |
| GO:0030315 | CC | T-tubule | 0.013345471482018 | 3 |
| GO:0098590 | CC | plasma membrane region | 0.0155607056082312 | 32 |
| GO:0019814 | CC | immunoglobulin complex | 0.0172331920436735 | 2 |
| GO:0030427 | CC | site of polarized growth | 0.0186838638009822 | 6 |
| GO:0043005 | CC | neuron projection | 0.0195819503114415 | 38 |
| GO:0015629 | CC | actin cytoskeleton | 0.0222537274410867 | 20 |
| GO:0005604 | CC | basement membrane | 0.0223869465005363 | 6 |
| GO:0005577 | CC | fibrinogen complex | 0.0251124678544974 | 2 |
| GO:0005614 | CC | interstitial matrix | 0.0251124678544974 | 2 |
| GO:0044853 | CC | plasma membrane raft | 0.0278875228523826 | 4 |
| GO:0098796 | CC | membrane protein complex | 0.0302101383816792 | 49 |
| GO:0048471 | CC | perinuclear region of cytoplasm | 0.0308324735570016 | 14 |
| GO:0045178 | CC | basal part of cell | 0.0348337476982471 | 9 |
| GO:0098794 | CC | postsynapse | 0.0357445933457848 | 18 |
| GO:0030139 | CC | endocytic vesicle | 0.0367328186538141 | 9 |
| GO:0005588 | CC | collagen type V trimer | 0.0434041922820532 | 1 |
| GO:0032144 | CC | 4-aminobutyrate transaminase complex | 0.0434041922820532 | 1 |
| GO:0034669 | CC | integrin alpha4-beta7 complex | 0.0434041922820532 | 1 |
| GO:0034777 | CC | recycling endosome lumen | 0.0434041922820532 | 1 |
| GO:0036194 | CC | muscle cell projection | 0.0434041922820532 | 1 |
| GO:0036195 | CC | muscle cell projection membrane | 0.0434041922820532 | 1 |
| GO:0042571 | CC | immunoglobulin complex, circulating | 0.0434041922820532 | 1 |
| GO:0044294 | CC | dendritic growth cone | 0.0434041922820532 | 1 |
| GO:0070110 | CC | ciliary neurotrophic factor receptor complex | 0.0434041922820532 | 1 |
| GO:0071664 | CC | catenin-TCF7L2 complex | 0.0434041922820532 | 1 |
| GO:0071683 | CC | sensory dendrite | 0.0434041922820532 | 1 |
| GO:0071745 | CC | IgA immunoglobulin complex | 0.0434041922820532 | 1 |
| GO:0071746 | CC | IgA immunoglobulin complex, circulating | 0.0434041922820532 | 1 |
| GO:0071748 | CC | monomeric IgA immunoglobulin complex | 0.0434041922820532 | 1 |
| GO:0071749 | CC | polymeric IgA immunoglobulin | 0.0434041922820532 | 1 |

| | | | | |
|---|---|---|---|---|
| | | complex | | |
| GO:0071750 | CC | dimeric IgA immunoglobulin complex | 0.0434041922820532 | 1 |
| GO:0071751 | CC | secretory IgA immunoglobulin complex | 0.0434041922820532 | 1 |
| GO:0071752 | CC | secretory dimeric IgA immunoglobulin complex | 0.0434041922820532 | 1 |
| GO:0071754 | CC | IgM immunoglobulin complex, circulating | 0.0434041922820532 | 1 |
| GO:0071755 | CC | IgM B cell receptor complex | 0.0434041922820532 | 1 |
| GO:0071756 | CC | pentameric IgM immunoglobulin complex | 0.0434041922820532 | 1 |
| GO:0097059 | CC | CNTFR-CLCF1 complex | 0.0434041922820532 | 1 |
| GO:0097512 | CC | cardiac myofibril | 0.0434041922820532 | 1 |
| GO:1901588 | CC | dendritic microtubule | 0.0434041922820532 | 1 |
| GO:1990578 | CC | perinuclear endoplasmic reticulum membrane | 0.0434041922820532 | 1 |
| GO:1990584 | CC | cardiac Troponin complex | 0.0434041922820532 | 1 |
| GO:0045121 | CC | membrane raft | 0.0437384923296646 | 7 |
| GO:0098857 | CC | membrane microdomain | 0.0437384923296646 | 7 |
| GO:0032584 | CC | growth cone membrane | 0.0442552977089355 | 2 |
| GO:0034703 | CC | cation channel complex | 0.0469145072974305 | 11 |
| GO:0032420 | CC | stereocilium | 0.0469781659913686 | 3 |
| GO:0043679 | CC | axon terminus | 0.0480524012065752 | 5 |
| GO:0042605 | MF | peptide antigen binding | 2.58576586316503e-06 | 9 |
| GO:0003823 | MF | antigen binding | 1.43985730338357e-05 | 9 |
| GO:0005201 | MF | extracellular matrix structural constituent | 2.39317407354846e-05 | 10 |
| GO:0015645 | MF | fatty acid ligase activity | 5.70171645895495e-05 | 6 |
| GO:0030020 | MF | extracellular matrix structural constituent conferring tensile strength | 0.000124807384526 | 7 |
| GO:0030527 | MF | structural constituent of chromatin | 0.00013335897686846 | 8 |
| GO:0016405 | MF | CoA-ligase activity | 0.0002848966606885 | 6 |
| GO:0099094 | MF | ligand-gated cation channel activity | 0.0003574094733361 | 12 |
| GO:0016878 | MF | acid-thiol ligase activity | 0.000604401906249 | 6 |
| GO:0017134 | MF | fibroblast growth factor binding | 0.0006250352503075 | 4 |
| GO:0044325 | MF | transmembrane transporter binding | 0.0006797577459979 | 9 |
| GO:0005102 | MF | signaling receptor binding | 0.0013077089655794 | 51 |
| GO:0004467 | MF | long-chain fatty acid-CoA ligase activity | 0.0013732638731503 | 4 |
| GO:0015276 | MF | ligand-gated ion channel activity | 0.0014555959548755 | 13 |
| GO:0022834 | MF | ligand-gated channel activity | 0.0014555959548755 | 13 |
| GO:0017159 | MF | pantetheine hydrolase activity | 0.0019253765873526 | 2 |

| | | | | |
|---|---|---|---|---|
| GO:0140260 | MF | mitochondrial proton-transporting ATP synthase complex binding | 0.0019253765873526 | 2 |
| GO:0005231 | MF | excitatory extracellular ligand-gated ion channel activity | 0.0020091256930366 | 6 |
| GO:0005230 | MF | extracellular ligand-gated ion channel activity | 0.0020225347200411 | 9 |
| GO:0005158 | MF | insulin receptor binding | 0.0025821609553052 | 3 |
| GO:0120013 | MF | lipid transfer activity | 0.0028058913400124 | 6 |
| GO:0022836 | MF | gated channel activity | 0.0032086231550412 | 21 |
| GO:0005215 | MF | transporter activity | 0.0035081134386353 | 59 |
| GO:0046983 | MF | protein dimerization activity | 0.0036470728839277 | 29 |
| GO:0016877 | MF | ligase activity, forming carbon-sulfur bonds | 0.0038182062149249 | 6 |
| GO:0005229 | MF | intracellular calcium activated chloride channel activity | 0.0039973154895556 | 3 |
| GO:0005347 | MF | ATP transmembrane transporter activity | 0.0039973154895556 | 3 |
| GO:0034056 | MF | estrogen response element binding | 0.0039973154895556 | 3 |
| GO:0061778 | MF | intracellular chloride channel activity | 0.0039973154895556 | 3 |
| GO:0019838 | MF | growth factor binding | 0.0040426148629563 | 8 |
| GO:0045499 | MF | chemorepellent activity | 0.0041029009558915 | 5 |
| GO:0004115 | MF | 3',5'-cyclic-AMP phosphodiesterase activity | 0.0043897060755787 | 4 |
| GO:0015605 | MF | organophosphate ester transmembrane transporter activity | 0.0048995662762638 | 5 |
| GO:0015267 | MF | channel activity | 0.0055314614380671 | 28 |
| GO:0022803 | MF | passive transmembrane transporter activity | 0.0055314614380671 | 28 |
| GO:0022848 | MF | acetylcholine-gated cation-selective channel activity | 0.0055437799105292 | 4 |
| GO:0004321 | MF | fatty-acyl-CoA synthase activity | 0.0056076250125236 | 2 |
| GO:0008526 | MF | phosphatidylinositol transfer activity | 0.0058017374191061 | 3 |
| GO:0030296 | MF | protein tyrosine kinase activator activity | 0.0058017374191061 | 3 |
| GO:0035091 | MF | phosphatidylinositol binding | 0.007843850072568 | 14 |
| GO:0005518 | MF | collagen binding | 0.0084220436705447 | 4 |
| GO:0008483 | MF | transaminase activity | 0.0084220436705447 | 4 |
| GO:0022857 | MF | transmembrane transporter activity | 0.009541574159414 | 53 |
| GO:0015215 | MF | nucleotide transmembrane transporter activity | 0.0101687512766475 | 4 |
| GO:0016769 | MF | transferase activity, transferring nitrogenous groups | 0.0101687512766475 | 4 |

| | | | | |
|---|---|---|---|---|
| GO:0030215 | MF | semaphorin receptor binding | 0.0101687512766475 | 4 |
| GO:0017154 | MF | semaphorin receptor activity | 0.0106723549054014 | 3 |
| GO:0043394 | MF | proteoglycan binding | 0.0108892806384668 | 2 |
| GO:0070974 | MF | POU domain binding | 0.0108892806384668 | 2 |
| GO:0046982 | MF | protein heterodimerization activity | 0.0129529080814848 | 11 |
| GO:1901981 | MF | phosphatidylinositol phosphate binding | 0.0149199224854557 | 9 |
| GO:0005261 | MF | cation channel activity | 0.0162742896469994 | 20 |
| GO:0022839 | MF | ion gated channel activity | 0.0167546845383006 | 4 |
| GO:0005007 | MF | fibroblast growth factor receptor activity | 0.0176232771633131 | 2 |
| GO:0016819 | MF | hydrolase activity, acting on acid anhydrides, in sulfonyl-containing anhydrides | 0.0176232771633131 | 2 |
| GO:0047627 | MF | adenylylsulfatase activity | 0.0176232771633131 | 2 |
| GO:0003700 | MF | DNA-binding transcription factor activity | 0.0176991161262414 | 49 |
| GO:0016836 | MF | hydro-lyase activity | 0.017890668386883 | 6 |
| GO:0005216 | MF | ion channel activity | 0.0193433814312145 | 24 |
| GO:0004114 | MF | 3',5'-cyclic-nucleotide phosphodiesterase activity | 0.0194249812167681 | 4 |
| GO:0022824 | MF | transmitter-gated ion channel activity | 0.0196556577102539 | 6 |
| GO:0022835 | MF | transmitter-gated channel activity | 0.0196556577102539 | 6 |
| GO:0000295 | MF | adenine nucleotide transmembrane transporter activity | 0.0213491068457808 | 3 |
| GO:0004089 | MF | carbonate dehydratase activity | 0.0213491068457808 | 3 |
| GO:0005346 | MF | purine ribonucleotide transmembrane transporter activity | 0.0213491068457808 | 3 |
| GO:0005319 | MF | lipid transporter activity | 0.0214092062310553 | 10 |
| GO:0099529 | MF | neurotransmitter receptor activity involved in regulation of postsynaptic membrane potential | 0.0218873758309803 | 5 |
| GO:1904315 | MF | transmitter-gated ion channel activity involved in regulation of postsynaptic membrane potential | 0.0218873758309803 | 5 |
| GO:0000978 | MF | RNA polymerase II cis-regulatory region sequence-specific DNA binding | 0.024228290325337 | 40 |
| GO:0008374 | MF | O-acyltransferase activity | 0.0243230369766126 | 5 |
| GO:0000981 | MF | DNA-binding transcription factor activity, RNA polymerase II-specific | 0.0245467921077867 | 46 |
| GO:0004112 | MF | cyclic-nucleotide phosphodiesterase activity | 0.0255150920438969 | 4 |

| | | | | |
|---|---|---|---|---|
| GO:0030551 | MF | cyclic nucleotide binding | 0.0255150920438969 | 4 |
| GO:0050840 | MF | extracellular matrix binding | 0.0255150920438969 | 4 |
| GO:0001091 | MF | RNA polymerase II general transcription initiation factor binding | 0.025672339754432 | 2 |
| GO:0031628 | MF | opioid receptor binding | 0.025672339754432 | 2 |
| GO:0070300 | MF | phosphatidic acid binding | 0.025672339754432 | 2 |
| GO:0003707 | MF | nuclear steroid receptor activity | 0.0258340758770006 | 3 |
| GO:0015216 | MF | purine nucleotide transmembrane transporter activity | 0.0258340758770006 | 3 |
| GO:0036122 | MF | BMP binding | 0.0258340758770006 | 3 |
| GO:0016835 | MF | carbon-oxygen lyase activity | 0.0278928304621198 | 6 |
| GO:0000977 | MF | RNA polymerase II transcription regulatory region sequence-specific DNA binding | 0.0280655808928443 | 45 |
| GO:0005515 | MF | protein binding | 0.030489788431739 | 168 |
| GO:0033218 | MF | amide binding | 0.0315926543261413 | 15 |
| GO:0000987 | MF | cis-regulatory region sequence-specific DNA binding | 0.0323712185447478 | 40 |
| GO:0019209 | MF | kinase activator activity | 0.0327544691387418 | 6 |
| GO:0015075 | MF | ion transmembrane transporter activity | 0.0333804137210749 | 38 |
| GO:0003841 | MF | 1-acylglycerol-3-phosphate O-acyltransferase activity | 0.0349084112740827 | 2 |
| GO:0004931 | MF | extracellularly ATP-gated cation channel activity | 0.0349084112740827 | 2 |
| GO:0010314 | MF | phosphatidylinositol-5-phosphate binding | 0.0349084112740827 | 2 |
| GO:0030297 | MF | transmembrane receptor protein tyrosine kinase activator activity | 0.0349084112740827 | 2 |
| GO:0038100 | MF | nodal binding | 0.0349084112740827 | 2 |
| GO:0043325 | MF | phosphatidylinositol-3,4-bisphosphate binding | 0.0349084112740827 | 2 |
| GO:1990247 | MF | N6-methyladenosine-containing RNA binding | 0.0349084112740827 | 2 |
| GO:0005164 | MF | tumor necrosis factor receptor binding | 0.0361911330339506 | 3 |
| GO:0008395 | MF | steroid hydroxylase activity | 0.0361911330339506 | 3 |
| GO:0120014 | MF | phospholipid transfer activity | 0.0361911330339506 | 3 |
| GO:0000976 | MF | transcription cis-regulatory region binding | 0.0363395534635772 | 47 |
| GO:0001067 | MF | transcription regulatory region nucleic acid binding | 0.0363395534635772 | 47 |
| GO:0004713 | MF | protein tyrosine kinase activity | 0.0370496208251855 | 9 |

| | | | | |
|---|---|---|---|---|
| GO:0008201 | MF | heparin binding | 0.0381350157550343 | 6 |
| GO:0005509 | MF | calcium ion binding | 0.0392906742176774 | 30 |
| GO:0005546 | MF | phosphatidylinositol-4,5-bisphosphate binding | 0.040797866093635 | 4 |
| GO:1990837 | MF | sequence-specific double-stranded DNA binding | 0.0409095514168404 | 48 |
| GO:0008324 | MF | cation transmembrane transporter activity | 0.0411106930349661 | 30 |
| GO:0003690 | MF | double-stranded DNA binding | 0.0425702187378382 | 50 |
| GO:0003829 | MF | beta-1,3-galactosyl-O-glycosyl-glycoprotein beta-1,6-N-acetylglucosaminyltransferase activity | 0.0439192020786187 | 1 |
| GO:0003867 | MF | 4-aminobutyrate transaminase activity | 0.0439192020786187 | 1 |
| GO:0004001 | MF | adenosine kinase activity | 0.0439192020786187 | 1 |
| GO:0004077 | MF | biotin-[acetyl-CoA-carboxylase] ligase activity | 0.0439192020786187 | 1 |
| GO:0004084 | MF | branched-chain-amino-acid transaminase activity | 0.0439192020786187 | 1 |
| GO:0004368 | MF | glycerol-3-phosphate dehydrogenase (quinone) activity | 0.0439192020786187 | 1 |
| GO:0005046 | MF | KDEL sequence binding | 0.0439192020786187 | 1 |
| GO:0005171 | MF | hepatocyte growth factor receptor binding | 0.0439192020786187 | 1 |
| GO:0005252 | MF | open rectifier potassium channel activity | 0.0439192020786187 | 1 |
| GO:0005497 | MF | androgen binding | 0.0439192020786187 | 1 |
| GO:0008445 | MF | D-aspartate oxidase activity | 0.0439192020786187 | 1 |
| GO:0008480 | MF | sarcosine dehydrogenase activity | 0.0439192020786187 | 1 |
| GO:0008743 | MF | L-threonine 3-dehydrogenase activity | 0.0439192020786187 | 1 |
| GO:0008781 | MF | N-acylneuraminate cytidylyltransferase activity | 0.0439192020786187 | 1 |
| GO:0015067 | MF | amidinotransferase activity | 0.0439192020786187 | 1 |
| GO:0015068 | MF | glycine amidinotransferase activity | 0.0439192020786187 | 1 |
| GO:0015188 | MF | L-isoleucine transmembrane transporter activity | 0.0439192020786187 | 1 |
| GO:0015190 | MF | L-leucine transmembrane transporter activity | 0.0439192020786187 | 1 |
| GO:0015191 | MF | L-methionine transmembrane transporter activity | 0.0439192020786187 | 1 |
| GO:0015225 | MF | biotin transmembrane transporter activity | 0.0439192020786187 | 1 |

| | | | | |
|---|---|---|---|---|
| GO:0016901 | MF | oxidoreductase activity, acting on the CH-OH group of donors, quinone or similar compound as acceptor | 0.0439192020786187 | 1 |
| GO:0017020 | MF | myosin phosphatase regulator activity | 0.0439192020786187 | 1 |
| GO:0018271 | MF | biotin-protein ligase activity | 0.0439192020786187 | 1 |
| GO:0019862 | MF | IgA binding | 0.0439192020786187 | 1 |
| GO:0022850 | MF | serotonin-gated cation-selective channel activity | 0.0439192020786187 | 1 |
| GO:0030504 | MF | inorganic diphosphate transmembrane transporter activity | 0.0439192020786187 | 1 |
| GO:0032896 | MF | palmitoyl-CoA 9-desaturase activity | 0.0439192020786187 | 1 |
| GO:0033787 | MF | cyanocobalamin reductase (cyanide-eliminating) activity | 0.0439192020786187 | 1 |
| GO:0034386 | MF | 4-aminobutyrate:2-oxoglutarate transaminase activity | 0.0439192020786187 | 1 |
| GO:0043125 | MF | ErbB-3 class receptor binding | 0.0439192020786187 | 1 |
| GO:0043262 | MF | ADP phosphatase activity | 0.0439192020786187 | 1 |
| GO:0043395 | MF | heparan sulfate proteoglycan binding | 0.0439192020786187 | 1 |
| GO:0043559 | MF | insulin binding | 0.0439192020786187 | 1 |
| GO:0043865 | MF | methionine transmembrane transporter activity | 0.0439192020786187 | 1 |
| GO:0044610 | MF | FMN transmembrane transporter activity | 0.0439192020786187 | 1 |
| GO:0046997 | MF | oxidoreductase activity, acting on the CH-NH group of donors, flavin as acceptor | 0.0439192020786187 | 1 |
| GO:0047024 | MF | 5alpha-androstane-3beta,17beta-diol dehydrogenase activity | 0.0439192020786187 | 1 |
| GO:0047298 | MF | (S)-3-amino-2-methylpropionate transaminase activity | 0.0439192020786187 | 1 |
| GO:0047961 | MF | glycine N-acyltransferase activity | 0.0439192020786187 | 1 |
| GO:0051978 | MF | lysophospholipid:sodium symporter activity | 0.0439192020786187 | 1 |
| GO:0052739 | MF | phosphatidylserine 1-acylhydrolase activity | 0.0439192020786187 | 1 |
| GO:0052740 | MF | 1-acyl-2-lysophosphatidylserine acylhydrolase activity | 0.0439192020786187 | 1 |
| GO:0060002 | MF | plus-end directed microfilament motor activity | 0.0439192020786187 | 1 |
| GO:0080122 | MF | AMP transmembrane transporter activity | 0.0439192020786187 | 1 |

| | | | | |
|---|---|---|---|---|
| GO:0106262 | MF | 1-acylglycerophosphoethanolamine O-acyltransferase activity | 0.0439192020786187 | 1 |
| GO:0106263 | MF | 1-acylglycerophosphoserine O-acyltransferase activity | 0.0439192020786187 | 1 |
| GO:0140314 | MF | calcium ion sequestering activity | 0.0439192020786187 | 1 |
| GO:1990698 | MF | palmitoleoyltransferase activity | 0.0439192020786187 | 1 |
| GO:0042277 | MF | peptide binding | 0.0447007436960292 | 13 |
| GO:0005520 | MF | insulin-like growth factor binding | 0.0452121098689251 | 2 |
| GO:0035381 | MF | ATP-gated ion channel activity | 0.0452121098689251 | 2 |
| GO:0042802 | MF | identical protein binding | 0.048073988266424 | 25 |
| GO:0015101 | MF | organic cation transmembrane transporter activity | 0.0483582836012472 | 3 |
| GO:0002376 | BP | immune system process | 3.28200112680485e-08 | 77 |
| GO:0007165 | BP | signal transduction | 4.8876301568979e-08 | 175 |
| GO:0007166 | BP | cell surface receptor signaling pathway | 4.93254729204669e-08 | 90 |
| GO:0050896 | BP | response to stimulus | 5.87977716525244e-08 | 242 |
| GO:0001913 | BP | T cell mediated cytotoxicity | 2.29273761738564e-07 | 11 |
| GO:0007154 | BP | cell communication | 2.59034079100392e-07 | 187 |
| GO:0009888 | BP | tissue development | 3.38606499722326e-07 | 54 |
| GO:0002428 | BP | antigen processing and presentation of peptide antigen via MHC class Ib | 3.45816721557524e-07 | 9 |
| GO:0002476 | BP | antigen processing and presentation of endogenous peptide antigen via MHC class Ib | 3.45816721557524e-07 | 9 |
| GO:0002484 | BP | antigen processing and presentation of endogenous peptide antigen via MHC class I via ER pathway | 3.45816721557524e-07 | 9 |
| GO:0002486 | BP | antigen processing and presentation of endogenous peptide antigen via MHC class I via ER pathway, TAP-independent | 3.45816721557524e-07 | 9 |
| GO:0048513 | BP | animal organ development | 6.38175060955871e-07 | 83 |
| GO:0002475 | BP | antigen processing and presentation via MHC class Ib | 7.96943477598439e-07 | 9 |
| GO:0023052 | BP | signaling | 9.55974840946197e-07 | 184 |
| GO:0002483 | BP | antigen processing and presentation of endogenous peptide antigen | 1.17026259659709e-06 | 9 |
| GO:0019885 | BP | antigen processing and presentation of endogenous peptide antigen via MHC class I | 1.17026259659709e-06 | 9 |
| GO:0001909 | BP | leukocyte mediated cytotoxicity | 1.30461377918324e-06 | 12 |
| GO:0032502 | BP | developmental process | 1.43615774342306e-06 | 151 |
| GO:0001914 | BP | regulation of T cell mediated | 1.63180213819504e-06 | 10 |

| | | | | |
|---|---|---|---|---|
| | | cytotoxicity | | |
| GO:0048583 | BP | regulation of response to stimulus | 1.9306490416079e-06 | 101 |
| GO:0001910 | BP | regulation of leukocyte mediated cytotoxicity | 2.90713813056375e-06 | 11 |
| GO:0019883 | BP | antigen processing and presentation of endogenous antigen | 3.32035320208577e-06 | 9 |
| GO:0001906 | BP | cell killing | 5.4698990754661e-06 | 13 |
| GO:0006955 | BP | immune response | 5.4865872391959e-06 | 53 |
| GO:0002474 | BP | antigen processing and presentation of peptide antigen via MHC class I | 6.16244823094438e-06 | 9 |
| GO:0001912 | BP | positive regulation of leukocyte mediated cytotoxicity | 6.44684154147695e-06 | 10 |
| GO:0031341 | BP | regulation of cell killing | 7.26406929916796e-06 | 11 |
| GO:0050789 | BP | regulation of biological process | 7.70830228502613e-06 | 299 |
| GO:0001916 | BP | positive regulation of T cell mediated cytotoxicity | 8.23285689759278e-06 | 9 |
| GO:0031343 | BP | positive regulation of cell killing | 8.25130837056221e-06 | 10 |
| GO:0048856 | BP | anatomical structure development | 1.22161212873233e-05 | 132 |
| GO:0002709 | BP | regulation of T cell mediated immunity | 1.65100443349347e-05 | 10 |
| GO:0051716 | BP | cellular response to stimulus | 3.69638985224698e-05 | 193 |
| GO:0048002 | BP | antigen processing and presentation of peptide antigen | 3.75202067726042e-05 | 9 |
| GO:0065007 | BP | biological regulation | 4.46818401249872e-05 | 314 |
| GO:0002250 | BP | adaptive immune response | 5.01512380493097e-05 | 23 |
| GO:0002711 | BP | positive regulation of T cell mediated immunity | 5.80460283336335e-05 | 9 |
| GO:0048009 | BP | insulin-like growth factor receptor signaling pathway | 6.75540779434387e-05 | 6 |
| GO:0045321 | BP | leukocyte activation | 7.08451278461449e-05 | 26 |
| GO:0002456 | BP | T cell mediated immunity | 7.34090844129272e-05 | 13 |
| GO:0001503 | BP | ossification | 8.01767463437707e-05 | 16 |
| GO:0001775 | BP | cell activation | 8.06081627483395e-05 | 29 |
| GO:0050794 | BP | regulation of cellular process | 8.58225586353028e-05 | 277 |
| GO:0048584 | BP | positive regulation of response to stimulus | 9.84809617880328e-05 | 54 |
| GO:0030154 | BP | cell differentiation | 0.0001010391208135 | 101 |
| GO:0002449 | BP | lymphocyte mediated immunity | 0.000111945229255 | 19 |
| GO:0048869 | BP | cellular developmental process | 0.0001171313161669 | 101 |
| GO:0002706 | BP | regulation of lymphocyte mediated immunity | 0.0001239404559636 | 11 |
| GO:0002708 | BP | positive regulation of lymphocyte mediated immunity | 0.0001316021758716 | 10 |
| GO:0002460 | BP | adaptive immune response based on | 0.0001825752608232 | 19 |

| | | | | |
|---|---|---|---|---|
| | | somatic recombination of immune receptors built from immunoglobulin superfamily domains | | |
| GO:0048675 | BP | axon extension | 0.0001835576220815 | 9 |
| GO:0002443 | BP | leukocyte mediated immunity | 0.0002014575525378 | 20 |
| GO:0007275 | BP | multicellular organism development | 0.0002105135149078 | 104 |
| GO:0048731 | BP | system development | 0.0002146275941983 | 97 |
| GO:0009653 | BP | anatomical structure morphogenesis | 0.0002300823598204 | 67 |
| GO:0002705 | BP | positive regulation of leukocyte mediated immunity | 0.0002438930101487 | 10 |
| GO:0043567 | BP | regulation of insulin-like growth factor receptor signaling pathway | 0.0002513225317475 | 4 |
| GO:0032501 | BP | multicellular organismal process | 0.0002543186503411 | 142 |
| GO:0046649 | BP | lymphocyte activation | 0.000261751758153 | 22 |
| GO:0030198 | BP | extracellular matrix organization | 0.0002721541922326 | 17 |
| GO:0045229 | BP | external encapsulating structure organization | 0.0002721541922326 | 17 |
| GO:1990138 | BP | neuron projection extension | 0.0002819819780038 | 10 |
| GO:0043062 | BP | extracellular structure organization | 0.0002956785582959 | 17 |
| GO:0009966 | BP | regulation of signal transduction | 0.0003507093076229 | 72 |
| GO:0002252 | BP | immune effector process | 0.0003509166686131 | 24 |
| GO:0002824 | BP | positive regulation of adaptive immune response based on somatic recombination of immune receptors built from immunoglobulin superfamily domains | 0.0004144394183053 | 9 |
| GO:1901222 | BP | regulation of NIK/NF-kappaB signaling | 0.0004367851268895 | 6 |
| GO:0071526 | BP | semaphorin-plexin signaling pathway | 0.0004694513509093 | 7 |
| GO:0019882 | BP | antigen processing and presentation | 0.0004814294620367 | 9 |
| GO:0002822 | BP | regulation of adaptive immune response based on somatic recombination of immune receptors built from immunoglobulin superfamily domains | 0.0004877006425092 | 10 |
| GO:0048762 | BP | mesenchymal cell differentiation | 0.0005043973784661 | 13 |
| GO:0002821 | BP | positive regulation of adaptive immune response | 0.000557059176642 | 9 |
| GO:0002819 | BP | regulation of adaptive immune response | 0.0007129822245403 | 10 |
| GO:0002703 | BP | regulation of leukocyte mediated immunity | 0.0007318574433504 | 11 |

| | | | | |
|---|---|---|---|---|
| GO:0048585 | BP | negative regulation of response to stimulus | 0.000796889387483 | 40 |
| GO:0051241 | BP | negative regulation of multicellular organismal process | 0.0007996581792701 | 22 |
| GO:0061564 | BP | axon development | 0.0007996581792701 | 22 |
| GO:0030516 | BP | regulation of axon extension | 0.0008337412580052 | 7 |
| GO:0050778 | BP | positive regulation of immune response | 0.0009371820727163 | 19 |
| GO:0072359 | BP | circulatory system development | 0.000981284458173 | 30 |
| GO:0030098 | BP | lymphocyte differentiation | 0.0010369089779904 | 14 |
| GO:1903131 | BP | mononuclear cell differentiation | 0.0010698917759955 | 15 |
| GO:0038061 | BP | NIK/NF-kappaB signaling | 0.0010977810369516 | 6 |
| GO:0002684 | BP | positive regulation of immune system process | 0.0011423353489277 | 25 |
| GO:0002699 | BP | positive regulation of immune effector process | 0.0012390502558869 | 11 |
| GO:0007167 | BP | enzyme-linked receptor protein signaling pathway | 0.0012688560897315 | 32 |
| GO:0000768 | BP | syncytium formation by plasma membrane fusion | 0.0012787926951998 | 5 |
| GO:0006949 | BP | syncytium formation | 0.0012787926951998 | 5 |
| GO:0140253 | BP | cell-cell fusion | 0.0012787926951998 | 5 |
| GO:0006730 | BP | one-carbon metabolic process | 0.0013444615185845 | 6 |
| GO:0048588 | BP | developmental cell growth | 0.0014218310547901 | 10 |
| GO:0003012 | BP | muscle system process | 0.0015437932314385 | 14 |
| GO:0055117 | BP | regulation of cardiac muscle contraction | 0.0016422772229991 | 5 |
| GO:0050793 | BP | regulation of developmental process | 0.0017438722360109 | 48 |
| GO:0060560 | BP | developmental growth involved in morphogenesis | 0.0017572671341303 | 10 |
| GO:0035556 | BP | intracellular signal transduction | 0.0017578912385191 | 76 |
| GO:0048534 | BP | hematopoietic or lymphoid organ development | 0.0019078543350026 | 22 |
| GO:0010648 | BP | negative regulation of cell communication | 0.0019578828619891 | 35 |
| GO:0043271 | BP | negative regulation of ion transport | 0.0019623222440463 | 6 |
| GO:0009968 | BP | negative regulation of signal transduction | 0.0019678619389164 | 33 |
| GO:0010646 | BP | regulation of cell communication | 0.0020413013286491 | 78 |
| GO:0015939 | BP | pantothenate metabolic process | 0.0020455882666764 | 2 |
| GO:0021636 | BP | trigeminal nerve morphogenesis | 0.0020455882666764 | 2 |
| GO:0021637 | BP | trigeminal nerve structural organization | 0.0020455882666764 | 2 |
| GO:0060485 | BP | mesenchyme development | 0.002063555761816 | 13 |

| | | | | |
|---|---|---|---|---|
| GO:0023057 | BP | negative regulation of signaling | 0.0021042176551252 | 35 |
| GO:0002521 | BP | leukocyte differentiation | 0.0021794553654002 | 17 |
| GO:0061387 | BP | regulation of extent of cell growth | 0.0022044347985163 | 7 |
| GO:0043408 | BP | regulation of MAPK cascade | 0.0022474569558727 | 20 |
| GO:0051216 | BP | cartilage development | 0.002377751913416 | 10 |
| GO:0051239 | BP | regulation of multicellular organismal process | 0.0025897618192211 | 50 |
| GO:0003013 | BP | circulatory system process | 0.0025920883369585 | 16 |
| GO:0003382 | BP | epithelial cell morphogenesis | 0.0028163378819628 | 3 |
| GO:0030097 | BP | hemopoiesis | 0.0028185395064884 | 21 |
| GO:0002697 | BP | regulation of immune effector process | 0.0029855587418363 | 12 |
| GO:0048646 | BP | anatomical structure formation involved in morphogenesis | 0.0030208627501856 | 27 |
| GO:0002682 | BP | regulation of immune system process | 0.0030521042115586 | 33 |
| GO:0008361 | BP | regulation of cell size | 0.0030686580708568 | 9 |
| GO:0000165 | BP | MAPK cascade | 0.0031117664707258 | 21 |
| GO:0048841 | BP | regulation of axon extension involved in axon guidance | 0.0031857866542967 | 5 |
| GO:0048846 | BP | axon extension involved in axon guidance | 0.0031857866542967 | 5 |
| GO:1902284 | BP | neuron projection extension involved in neuron projection guidance | 0.0031857866542967 | 5 |
| GO:0060048 | BP | cardiac muscle contraction | 0.0033428700480041 | 7 |
| GO:0061448 | BP | connective tissue development | 0.0033918057302339 | 11 |
| GO:0023051 | BP | regulation of signaling | 0.0035496426252576 | 77 |
| GO:0060047 | BP | heart contraction | 0.0037921973370074 | 10 |
| GO:0006942 | BP | regulation of striated muscle contraction | 0.0038766967958779 | 5 |
| GO:0000904 | BP | cell morphogenesis involved in differentiation | 0.0043474759504462 | 24 |
| GO:0016477 | BP | cell migration | 0.0043564183597663 | 36 |
| GO:0001659 | BP | temperature homeostasis | 0.0044299149736031 | 6 |
| GO:0003015 | BP | heart process | 0.0045166716695837 | 10 |
| GO:0006936 | BP | muscle contraction | 0.0046938190439106 | 12 |
| GO:0002520 | BP | immune system development | 0.0047122014412344 | 22 |
| GO:0006941 | BP | striated muscle contraction | 0.0048503760067663 | 8 |
| GO:0050770 | BP | regulation of axonogenesis | 0.0048503760067663 | 8 |
| GO:0008015 | BP | blood circulation | 0.0050369554386063 | 15 |
| GO:0007517 | BP | muscle organ development | 0.0050589437044189 | 11 |
| GO:0001501 | BP | skeletal system development | 0.0052668218788302 | 16 |
| GO:0030217 | BP | T cell differentiation | 0.0053459044412287 | 10 |

| | | | | |
|---|---|---|---|---|
| GO:0050776 | BP | regulation of immune response | 0.0054880920567524 | 23 |
| GO:0001837 | BP | epithelial to mesenchymal transition | 0.0054982689782408 | 7 |
| GO:0050954 | BP | sensory perception of mechanical stimulus | 0.0054982689782408 | 7 |
| GO:0061061 | BP | muscle structure development | 0.0058034831214838 | 19 |
| GO:0010881 | BP | regulation of cardiac muscle contraction by regulation of the release of sequestered calcium ion | 0.0059522105737963 | 2 |
| GO:0050852 | BP | T cell receptor signaling pathway | 0.0059541167229578 | 8 |
| GO:0040011 | BP | locomotion | 0.0061095063043902 | 32 |
| GO:0030334 | BP | regulation of cell migration | 0.0062330780744016 | 21 |
| GO:0001574 | BP | ganglioside biosynthetic process | 0.006314915728939 | 3 |
| GO:0007409 | BP | axonogenesis | 0.0063836066286928 | 18 |
| GO:0006633 | BP | fatty acid biosynthetic process | 0.0065564902354895 | 9 |
| GO:0090257 | BP | regulation of muscle system process | 0.0069024359094201 | 7 |
| GO:0042330 | BP | taxis | 0.0072304895707762 | 20 |
| GO:0072330 | BP | monocarboxylic acid biosynthetic process | 0.007358371553766 | 10 |
| GO:0035914 | BP | skeletal muscle cell differentiation | 0.0077241875064345 | 5 |
| GO:0045595 | BP | regulation of cell differentiation | 0.0080647241157009 | 31 |
| GO:0090287 | BP | regulation of cellular response to growth factor stimulus | 0.0084132424039314 | 11 |
| GO:0050851 | BP | antigen receptor-mediated signaling pathway | 0.0084521771234297 | 9 |
| GO:0001502 | BP | cartilage condensation | 0.0087208501539739 | 3 |
| GO:0043368 | BP | positive T cell selection | 0.0087208501539739 | 3 |
| GO:0048678 | BP | response to axon injury | 0.0087208501539739 | 3 |
| GO:0051926 | BP | negative regulation of calcium ion transport | 0.0087208501539739 | 3 |
| GO:1902285 | BP | semaphorin-plexin signaling pathway involved in neuron projection guidance | 0.0087208501539739 | 3 |
| GO:1902287 | BP | semaphorin-plexin signaling pathway involved in axon guidance | 0.0087208501539739 | 3 |
| GO:0042110 | BP | T cell activation | 0.009043661291553 | 14 |
| GO:0001667 | BP | ameboidal-type cell migration | 0.0091892075018973 | 13 |
| GO:0034763 | BP | negative regulation of transmembrane transport | 0.0093546732936129 | 4 |
| GO:0051149 | BP | positive regulation of muscle cell differentiation | 0.0093546732936129 | 4 |
| GO:0006937 | BP | regulation of muscle contraction | 0.009765842816824 | 6 |
| GO:0090497 | BP | mesenchymal cell migration | 0.009765842816824 | 6 |
| GO:0040012 | BP | regulation of locomotion | 0.009933263881758 | 22 |
| GO:0007169 | BP | transmembrane receptor protein | 0.0101697092227071 | 20 |

| | | | | |
|---|---|---|---|---|
| | | tyrosine kinase signaling pathway | | |
| GO:0043410 | BP | positive regulation of MAPK cascade | 0.0103171582590259 | 13 |
| GO:0035295 | BP | tube development | 0.0103239431212505 | 22 |
| GO:0007519 | BP | skeletal muscle tissue development | 0.0104846084569384 | 7 |
| GO:0040017 | BP | positive regulation of locomotion | 0.0106485639369506 | 14 |
| GO:0048518 | BP | positive regulation of biological process | 0.010709584398732 | 118 |
| GO:0006575 | BP | cellular modified amino acid metabolic process | 0.0107376414051203 | 9 |
| GO:0014902 | BP | myotube differentiation | 0.0109694611135693 | 6 |
| GO:0009887 | BP | animal organ morphogenesis | 0.0115248736992306 | 23 |
| GO:0001991 | BP | regulation of systemic arterial blood pressure by circulatory renin-angiotensin | 0.011547778879586 | 2 |
| GO:0010649 | BP | regulation of cell communication by electrical coupling | 0.011547778879586 | 2 |
| GO:0019731 | BP | antibacterial humoral response | 0.011547778879586 | 2 |
| GO:0021604 | BP | cranial nerve structural organization | 0.011547778879586 | 2 |
| GO:0038098 | BP | sequestering of BMP from receptor via BMP binding | 0.011547778879586 | 2 |
| GO:0045059 | BP | positive thymic T cell selection | 0.011547778879586 | 2 |
| GO:0048012 | BP | hepatocyte growth factor receptor signaling pathway | 0.011547778879586 | 2 |
| GO:0060244 | BP | negative regulation of cell proliferation involved in contact inhibition | 0.011547778879586 | 2 |
| GO:0070934 | BP | CRD-mediated mRNA stabilization | 0.011547778879586 | 2 |
| GO:0106070 | BP | regulation of adenylate cyclase-activating G protein-coupled receptor signaling pathway | 0.011547778879586 | 2 |
| GO:0035336 | BP | long-chain fatty-acyl-CoA metabolic process | 0.011592760964253 | 3 |
| GO:0035987 | BP | endodermal cell differentiation | 0.011592760964253 | 3 |
| GO:0098743 | BP | cell aggregation | 0.011592760964253 | 3 |
| GO:0042221 | BP | response to chemical | 0.0119476127634531 | 75 |
| GO:2000145 | BP | regulation of cell motility | 0.0120196615276109 | 21 |
| GO:0050905 | BP | neuromuscular process | 0.0122743454502812 | 6 |
| GO:0003008 | BP | system process | 0.0124448860710901 | 36 |
| GO:0002429 | BP | immune response-activating cell surface receptor signaling pathway | 0.0125002515928896 | 9 |
| GO:0002757 | BP | immune response-activating signal transduction | 0.0125002515928896 | 9 |

| | | | | |
|---|---|---|---|---|
| GO:0006935 | BP | chemotaxis | 0.013381345157505 | 19 |
| GO:0035239 | BP | tube morphogenesis | 0.013381345157505 | 19 |
| GO:0048667 | BP | cell morphogenesis involved in neuron differentiation | 0.013381345157505 | 19 |
| GO:0030199 | BP | collagen fibril organization | 0.013449020215878 | 4 |
| GO:0038066 | BP | p38MAPK cascade | 0.013449020215878 | 4 |
| GO:0048843 | BP | negative regulation of axon extension involved in axon guidance | 0.013449020215878 | 4 |
| GO:0070167 | BP | regulation of biomineral tissue development | 0.013449020215878 | 4 |
| GO:0110149 | BP | regulation of biomineralization | 0.013449020215878 | 4 |
| GO:0030335 | BP | positive regulation of cell migration | 0.0136103281373546 | 13 |
| GO:0060538 | BP | skeletal muscle organ development | 0.0139426297356973 | 7 |
| GO:0019221 | BP | cytokine-mediated signaling pathway | 0.0141205216420124 | 11 |
| GO:0048870 | BP | cell motility | 0.0144030867590137 | 37 |
| GO:0031175 | BP | neuron projection development | 0.0144973754185991 | 26 |
| GO:0009068 | BP | aspartate family amino acid catabolic process | 0.0149447036580525 | 3 |
| GO:0009595 | BP | detection of biotic stimulus | 0.0149447036580525 | 3 |
| GO:0030520 | BP | intracellular estrogen receptor signaling pathway | 0.0149447036580525 | 3 |
| GO:1901223 | BP | negative regulation of NIK/NF-kappaB signaling | 0.0149447036580525 | 3 |
| GO:0030182 | BP | neuron differentiation | 0.0149560511578215 | 36 |
| GO:2000026 | BP | regulation of multicellular organismal development | 0.0150870456756855 | 27 |
| GO:1901700 | BP | response to oxygen-containing compound | 0.0151596411388202 | 28 |
| GO:0000902 | BP | cell morphogenesis | 0.0151908010112152 | 29 |
| GO:0002768 | BP | immune response-regulating cell surface receptor signaling pathway | 0.0155360947768618 | 9 |
| GO:0006636 | BP | unsaturated fatty acid biosynthetic process | 0.0158625407912733 | 4 |
| GO:0033077 | BP | T cell differentiation in thymus | 0.0158625407912733 | 4 |
| GO:1905330 | BP | regulation of morphogenesis of an epithelium | 0.0158625407912733 | 4 |
| GO:0051146 | BP | striated muscle cell differentiation | 0.0159358653768297 | 10 |
| GO:0120035 | BP | regulation of plasma membrane bounded cell projection organization | 0.0166857539851157 | 15 |
| GO:0048729 | BP | tissue morphogenesis | 0.0168241779668012 | 14 |
| GO:0007605 | BP | sensory perception of sound | 0.0168367699645165 | 6 |

| | | | | |
|---|---|---|---|---|
| GO:0051403 | BP | stress-activated MAPK cascade | 0.0169940701567084 | 8 |
| GO:0042981 | BP | regulation of apoptotic process | 0.0170736056627456 | 29 |
| GO:0048863 | BP | stem cell differentiation | 0.0178404829037881 | 9 |
| GO:0090092 | BP | regulation of transmembrane receptor protein serine/threonine kinase signaling pathway | 0.0178404829037881 | 9 |
| GO:0008016 | BP | regulation of heart contraction | 0.0181482954525668 | 7 |
| GO:0009967 | BP | positive regulation of signal transduction | 0.0182474088559058 | 32 |
| GO:0031098 | BP | stress-activated protein kinase signaling cascade | 0.0183262713238636 | 8 |
| GO:0022612 | BP | gland morphogenesis | 0.0185313311358999 | 4 |
| GO:0030517 | BP | negative regulation of axon extension | 0.0185313311358999 | 4 |
| GO:0010882 | BP | regulation of cardiac muscle contraction by calcium ion signaling | 0.0186719241356975 | 2 |
| GO:0021559 | BP | trigeminal nerve development | 0.0186719241356975 | 2 |
| GO:0033148 | BP | positive regulation of intracellular estrogen receptor signaling pathway | 0.0186719241356975 | 2 |
| GO:0034143 | BP | regulation of toll-like receptor 4 signaling pathway | 0.0186719241356975 | 2 |
| GO:0035581 | BP | sequestering of extracellular ligand from receptor | 0.0186719241356975 | 2 |
| GO:0043931 | BP | ossification involved in bone maturation | 0.0186719241356975 | 2 |
| GO:0045721 | BP | negative regulation of gluconeogenesis | 0.0186719241356975 | 2 |
| GO:0048743 | BP | positive regulation of skeletal muscle fiber development | 0.0186719241356975 | 2 |
| GO:0050849 | BP | negative regulation of calcium-mediated signaling | 0.0186719241356975 | 2 |
| GO:0050860 | BP | negative regulation of T cell receptor signaling pathway | 0.0186719241356975 | 2 |
| GO:0060242 | BP | contact inhibition | 0.0186719241356975 | 2 |
| GO:0060245 | BP | detection of cell density | 0.0186719241356975 | 2 |
| GO:0070168 | BP | negative regulation of biomineral tissue development | 0.0186719241356975 | 2 |
| GO:0070885 | BP | negative regulation of calcineurin-NFAT signaling cascade | 0.0186719241356975 | 2 |
| GO:0086004 | BP | regulation of cardiac muscle cell contraction | 0.0186719241356975 | 2 |
| GO:0106057 | BP | negative regulation of calcineurin-mediated signaling | 0.0186719241356975 | 2 |
| GO:0110150 | BP | negative regulation of | 0.0186719241356975 | 2 |

| | | | | |
|---|---|---|---|---|
| | | biomineralization | | |
| GO:1900115 | BP | extracellular regulation of signal transduction | 0.0186719241356975 | 2 |
| GO:1900116 | BP | extracellular negative regulation of signal transduction | 0.0186719241356975 | 2 |
| GO:1990349 | BP | gap junction-mediated intercellular transport | 0.0186719241356975 | 2 |
| GO:0043029 | BP | T cell homeostasis | 0.0187857681922919 | 3 |
| GO:0045058 | BP | T cell selection | 0.0187857681922919 | 3 |
| GO:1900744 | BP | regulation of p38MAPK cascade | 0.0187857681922919 | 3 |
| GO:1904063 | BP | negative regulation of cation transmembrane transport | 0.0187857681922919 | 3 |
| GO:0007411 | BP | axon guidance | 0.0193819894940986 | 12 |
| GO:2000147 | BP | positive regulation of cell motility | 0.0195087486167546 | 13 |
| GO:0022603 | BP | regulation of anatomical structure morphogenesis | 0.0195863153651798 | 20 |
| GO:0032872 | BP | regulation of stress-activated MAPK cascade | 0.0197290223249236 | 7 |
| GO:0070302 | BP | regulation of stress-activated protein kinase signaling cascade | 0.0197290223249236 | 7 |
| GO:0010243 | BP | response to organonitrogen compound | 0.0197311357824066 | 19 |
| GO:0007507 | BP | heart development | 0.0203306893084029 | 16 |
| GO:0097485 | BP | neuron projection guidance | 0.0204193367970531 | 12 |
| GO:0031344 | BP | regulation of cell projection organization | 0.0208633589298314 | 15 |
| GO:0016049 | BP | cell growth | 0.0211710824063738 | 11 |
| GO:0000038 | BP | very long-chain fatty acid metabolic process | 0.0214622580039042 | 4 |
| GO:0106106 | BP | cold-induced thermogenesis | 0.0214622580039042 | 4 |
| GO:0120161 | BP | regulation of cold-induced thermogenesis | 0.0214622580039042 | 4 |
| GO:0060070 | BP | canonical Wnt signaling pathway | 0.021502578597123 | 13 |
| GO:0018108 | BP | peptidyl-tyrosine phosphorylation | 0.0224492015686035 | 6 |
| GO:0018212 | BP | peptidyl-tyrosine modification | 0.0224492015686035 | 6 |
| GO:1905475 | BP | regulation of protein localization to membrane | 0.0224492015686035 | 6 |
| GO:1903522 | BP | regulation of blood circulation | 0.022769491007914 | 8 |
| GO:0007155 | BP | cell adhesion | 0.0230013304651324 | 38 |
| GO:0034766 | BP | negative regulation of ion transmembrane transport | 0.0231206209968178 | 3 |
| GO:0035337 | BP | fatty-acyl-CoA metabolic process | 0.0231206209968178 | 3 |
| GO:0042761 | BP | very long-chain fatty acid biosynthetic process | 0.0231206209968178 | 3 |

| | | | | |
|---|---|---|---|---|
| GO:0051155 | BP | positive regulation of striated muscle cell differentiation | 0.0231206209968178 | 3 |
| GO:0010975 | BP | regulation of neuron projection development | 0.0235928706575347 | 11 |
| GO:0010941 | BP | regulation of cell death | 0.023858212064005 | 29 |
| GO:0043067 | BP | regulation of programmed cell death | 0.023858212064005 | 29 |
| GO:0050920 | BP | regulation of chemotaxis | 0.0250430064180884 | 7 |
| GO:0030155 | BP | regulation of cell adhesion | 0.025788638012498 | 15 |
| GO:0042113 | BP | B cell activation | 0.0261226350020352 | 8 |
| GO:0070887 | BP | cellular response to chemical stimulus | 0.02613493531793 | 57 |
| GO:0048812 | BP | neuron projection morphogenesis | 0.0263133053653499 | 19 |
| GO:0014032 | BP | neural crest cell development | 0.0268188822846821 | 6 |
| GO:0045765 | BP | regulation of angiogenesis | 0.0270127054968988 | 7 |
| GO:0048522 | BP | positive regulation of cellular process | 0.0270640636372939 | 103 |
| GO:1901698 | BP | response to nitrogen compound | 0.0270828455685255 | 21 |
| GO:0006658 | BP | phosphatidylserine metabolic process | 0.0271752910083946 | 2 |
| GO:0010644 | BP | cell communication by electrical coupling | 0.0271752910083946 | 2 |
| GO:0031103 | BP | axon regeneration | 0.0271752910083946 | 2 |
| GO:0031649 | BP | heat generation | 0.0271752910083946 | 2 |
| GO:0033145 | BP | positive regulation of intracellular steroid hormone receptor signaling pathway | 0.0271752910083946 | 2 |
| GO:0033615 | BP | mitochondrial proton-transporting ATP synthase complex assembly | 0.0271752910083946 | 2 |
| GO:0043461 | BP | proton-transporting ATP synthase complex assembly | 0.0271752910083946 | 2 |
| GO:0046851 | BP | negative regulation of bone remodeling | 0.0271752910083946 | 2 |
| GO:0048532 | BP | anatomical structure arrangement | 0.0271752910083946 | 2 |
| GO:0048643 | BP | positive regulation of skeletal muscle tissue development | 0.0271752910083946 | 2 |
| GO:0048742 | BP | regulation of skeletal muscle fiber development | 0.0271752910083946 | 2 |
| GO:0050858 | BP | negative regulation of antigen receptor-mediated signaling pathway | 0.0271752910083946 | 2 |
| GO:0070977 | BP | bone maturation | 0.0271752910083946 | 2 |
| GO:0072378 | BP | blood coagulation, fibrin clot formation | 0.0271752910083946 | 2 |

| | | | | |
|---|---|---|---|---|
| GO:0080154 | BP | regulation of fertilization | 0.0271752910083946 | 2 |
| GO:0097242 | BP | amyloid-beta clearance | 0.0271752910083946 | 2 |
| GO:1903170 | BP | negative regulation of calcium ion transmembrane transport | 0.0271752910083946 | 2 |
| GO:0003009 | BP | skeletal muscle contraction | 0.0279500027818864 | 3 |
| GO:0007520 | BP | myoblast fusion | 0.0279500027818864 | 3 |
| GO:0019730 | BP | antimicrobial humoral response | 0.0279500027818864 | 3 |
| GO:0043500 | BP | muscle adaptation | 0.0279500027818864 | 3 |
| GO:0070231 | BP | T cell apoptotic process | 0.0279500027818864 | 3 |
| GO:0120162 | BP | positive regulation of cold-induced thermogenesis | 0.0279500027818864 | 3 |
| GO:0010717 | BP | regulation of epithelial to mesenchymal transition | 0.0281323834402262 | 4 |
| GO:0050771 | BP | negative regulation of axonogenesis | 0.0281323834402262 | 4 |
| GO:0050922 | BP | negative regulation of chemotaxis | 0.0281323834402262 | 4 |
| GO:0009605 | BP | response to external stimulus | 0.0285982275620421 | 48 |
| GO:0032989 | BP | cellular component morphogenesis | 0.0290312045451986 | 22 |
| GO:0030510 | BP | regulation of BMP signaling pathway | 0.0292009335130849 | 6 |
| GO:0051051 | BP | negative regulation of transport | 0.0295730349634314 | 9 |
| GO:0046631 | BP | alpha-beta T cell activation | 0.0301735230166043 | 5 |
| GO:0120039 | BP | plasma membrane bounded cell projection morphogenesis | 0.0311986960441188 | 19 |
| GO:0048864 | BP | stem cell development | 0.0317178880201324 | 6 |
| GO:0048666 | BP | neuron development | 0.0317710751540674 | 27 |
| GO:0009719 | BP | response to endogenous stimulus | 0.0317765681746619 | 32 |
| GO:0009066 | BP | aspartate family amino acid metabolic process | 0.0318797630432412 | 4 |
| GO:0030518 | BP | intracellular steroid hormone receptor signaling pathway | 0.0318797630432412 | 4 |
| GO:0002009 | BP | morphogenesis of an epithelium | 0.0320662500241677 | 11 |
| GO:0046394 | BP | carboxylic acid biosynthetic process | 0.0323952185614456 | 13 |
| GO:0001755 | BP | neural crest cell migration | 0.0332469841186979 | 5 |
| GO:0001573 | BP | ganglioside metabolic process | 0.0332711852602904 | 3 |
| GO:0001706 | BP | endoderm formation | 0.0332711852602904 | 3 |
| GO:0006688 | BP | glycosphingolipid biosynthetic process | 0.0332711852602904 | 3 |
| GO:0010830 | BP | regulation of myotube differentiation | 0.0332711852602904 | 3 |
| GO:0032990 | BP | cell part morphogenesis | 0.0333317912568579 | 19 |
| GO:0048858 | BP | cell projection morphogenesis | 0.0333317912568579 | 19 |
| GO:1901342 | BP | regulation of vasculature development | 0.0335465297044119 | 7 |
| GO:0070661 | BP | leukocyte proliferation | 0.0338296668551473 | 8 |

| | | | | |
|---|---|---|---|---|
| GO:0014033 | BP | neural crest cell differentiation | 0.0343720479655316 | 6 |
| GO:0016053 | BP | organic acid biosynthetic process | 0.0352764447998679 | 13 |
| GO:0014070 | BP | response to organic cyclic compound | 0.0354803961980317 | 14 |
| GO:0002253 | BP | activation of immune response | 0.0355170461421732 | 10 |
| GO:0048699 | BP | generation of neurons | 0.0358137722066675 | 36 |
| GO:0008643 | BP | carbohydrate transport | 0.0359056922993756 | 4 |
| GO:0043401 | BP | steroid hormone mediated signaling pathway | 0.0359056922993756 | 4 |
| GO:1990845 | BP | adaptive thermogenesis | 0.0359056922993756 | 4 |
| GO:0002062 | BP | chondrocyte differentiation | 0.0365082537439581 | 5 |
| GO:0003081 | BP | regulation of systemic arterial blood pressure by renin-angiotensin | 0.0369188722589672 | 2 |
| GO:0006654 | BP | phosphatidic acid biosynthetic process | 0.0369188722589672 | 2 |
| GO:0010496 | BP | intercellular transport | 0.0369188722589672 | 2 |
| GO:0010880 | BP | regulation of release of sequestered calcium ion into cytosol by sarcoplasmic reticulum | 0.0369188722589672 | 2 |
| GO:0031954 | BP | positive regulation of protein autophosphorylation | 0.0369188722589672 | 2 |
| GO:0034104 | BP | negative regulation of tissue remodeling | 0.0369188722589672 | 2 |
| GO:0035338 | BP | long-chain fatty-acyl-CoA biosynthetic process | 0.0369188722589672 | 2 |
| GO:0044539 | BP | long-chain fatty acid import into cell | 0.0369188722589672 | 2 |
| GO:0045061 | BP | thymic T cell selection | 0.0369188722589672 | 2 |
| GO:0046473 | BP | phosphatidic acid metabolic process | 0.0369188722589672 | 2 |
| GO:0048641 | BP | regulation of skeletal muscle tissue development | 0.0369188722589672 | 2 |
| GO:0048799 | BP | animal organ maturation | 0.0369188722589672 | 2 |
| GO:0060343 | BP | trabecula formation | 0.0369188722589672 | 2 |
| GO:0070229 | BP | negative regulation of lymphocyte apoptotic process | 0.0369188722589672 | 2 |
| GO:0070233 | BP | negative regulation of T cell apoptotic process | 0.0369188722589672 | 2 |
| GO:0071391 | BP | cellular response to estrogen stimulus | 0.0369188722589672 | 2 |
| GO:0072376 | BP | protein activation cascade | 0.0369188722589672 | 2 |
| GO:1901863 | BP | positive regulation of muscle tissue development | 0.0369188722589672 | 2 |
| GO:1903115 | BP | regulation of actin filament-based movement | 0.0369188722589672 | 2 |
| GO:0060537 | BP | muscle tissue development | 0.0370287307125891 | 11 |

| | | | | |
|---|---|---|---|---|
| GO:0042127 | BP | regulation of cell population proliferation | 0.0371110151539106 | 27 |
| GO:0010721 | BP | negative regulation of cell development | 0.037165493272497 | 6 |
| GO:0002260 | BP | lymphocyte homeostasis | 0.0390783897037449 | 3 |
| GO:0008038 | BP | neuron recognition | 0.0390783897037449 | 3 |
| GO:0032787 | BP | monocarboxylic acid metabolic process | 0.0391363270702996 | 19 |
| GO:0010647 | BP | positive regulation of cell communication | 0.0400150790845092 | 34 |
| GO:0090101 | BP | negative regulation of transmembrane receptor protein serine/threonine kinase signaling pathway | 0.0401000774324666 | 6 |
| GO:0048519 | BP | negative regulation of biological process | 0.0403662109107948 | 95 |
| GO:0023056 | BP | positive regulation of signaling | 0.0417770281208528 | 34 |
| GO:1901701 | BP | cellular response to oxygen-containing compound | 0.0427788778211311 | 21 |
| GO:0051093 | BP | negative regulation of developmental process | 0.0433881361040354 | 16 |
| GO:0044283 | BP | small molecule biosynthetic process | 0.0442212771761901 | 20 |
| GO:0031589 | BP | cell-substrate adhesion | 0.0444493966148491 | 11 |
| GO:0071495 | BP | cellular response to endogenous stimulus | 0.0445957419891301 | 29 |
| GO:0002027 | BP | regulation of heart rate | 0.0447980752826008 | 4 |
| GO:0000183 | BP | rDNA heterochromatin formation | 0.0452674897119342 | 1 |
| GO:0000378 | BP | RNA exon ligation | 0.0452674897119342 | 1 |
| GO:0000413 | BP | protein peptidyl-prolyl isomerization | 0.0452674897119342 | 1 |
| GO:0000968 | BP | tRNA exon ligation | 0.0452674897119342 | 1 |
| GO:0000971 | BP | tRNA exon ligation utilizing 2',3' cyclic phosphate of 5'-exon as source of linkage phosphate | 0.0452674897119342 | 1 |
| GO:0001714 | BP | endodermal cell fate specification | 0.0452674897119342 | 1 |
| GO:0001886 | BP | endothelial cell morphogenesis | 0.0452674897119342 | 1 |
| GO:0001999 | BP | renal response to blood flow involved in circulatory renin-angiotensin regulation of systemic arterial blood pressure | 0.0452674897119342 | 1 |
| GO:0002001 | BP | renin secretion into blood stream | 0.0452674897119342 | 1 |
| GO:0002302 | BP | CD8-positive, alpha-beta T cell differentiation involved in immune response | 0.0452674897119342 | 1 |

| | | | | |
|---|---|---|---|---|
| GO:0003084 | BP | positive regulation of systemic arterial blood pressure | 0.0452674897119342 | 1 |
| GO:0003190 | BP | atrioventricular valve formation | 0.0452674897119342 | 1 |
| GO:0003345 | BP | proepicardium cell migration involved in pericardium morphogenesis | 0.0452674897119342 | 1 |
| GO:0003366 | BP | cell-matrix adhesion involved in ameboidal cell migration | 0.0452674897119342 | 1 |
| GO:0006931 | BP | substrate-dependent cell migration, cell attachment to substrate | 0.0452674897119342 | 1 |
| GO:0009087 | BP | methionine catabolic process | 0.0452674897119342 | 1 |
| GO:0009098 | BP | leucine biosynthetic process | 0.0452674897119342 | 1 |
| GO:0010813 | BP | neuropeptide catabolic process | 0.0452674897119342 | 1 |
| GO:0010814 | BP | substance P catabolic process | 0.0452674897119342 | 1 |
| GO:0010815 | BP | bradykinin catabolic process | 0.0452674897119342 | 1 |
| GO:0010961 | BP | cellular magnesium ion homeostasis | 0.0452674897119342 | 1 |
| GO:0014850 | BP | response to muscle activity | 0.0452674897119342 | 1 |
| GO:0014854 | BP | response to inactivity | 0.0452674897119342 | 1 |
| GO:0014870 | BP | response to muscle inactivity | 0.0452674897119342 | 1 |
| GO:0014874 | BP | response to stimulus involved in regulation of muscle adaptation | 0.0452674897119342 | 1 |
| GO:0014877 | BP | response to muscle inactivity involved in regulation of muscle adaptation | 0.0452674897119342 | 1 |
| GO:0014894 | BP | response to denervation involved in regulation of muscle adaptation | 0.0452674897119342 | 1 |
| GO:0015818 | BP | isoleucine transport | 0.0452674897119342 | 1 |
| GO:0018021 | BP | peptidyl-histidine methylation | 0.0452674897119342 | 1 |
| GO:0018208 | BP | peptidyl-proline modification | 0.0452674897119342 | 1 |
| GO:0019102 | BP | male somatic sex determination | 0.0452674897119342 | 1 |
| GO:0019481 | BP | L-alanine catabolic process, by transamination | 0.0452674897119342 | 1 |
| GO:0021564 | BP | vagus nerve development | 0.0452674897119342 | 1 |
| GO:0021644 | BP | vagus nerve morphogenesis | 0.0452674897119342 | 1 |
| GO:0021773 | BP | striatal medium spiny neuron differentiation | 0.0452674897119342 | 1 |
| GO:0021784 | BP | postganglionic parasympathetic fiber development | 0.0452674897119342 | 1 |
| GO:0021785 | BP | branchiomotor neuron axon guidance | 0.0452674897119342 | 1 |
| GO:0021793 | BP | chemorepulsion of branchiomotor axon | 0.0452674897119342 | 1 |
| GO:0030047 | BP | actin modification | 0.0452674897119342 | 1 |
| GO:0031000 | BP | response to caffeine | 0.0452674897119342 | 1 |

| | | | | |
|---|---|---|---|---|
| GO:0031652 | BP | positive regulation of heat generation | 0.0452674897119342 | 1 |
| GO:0032026 | BP | response to magnesium ion | 0.0452674897119342 | 1 |
| GO:0032286 | BP | central nervous system myelin maintenance | 0.0452674897119342 | 1 |
| GO:0032515 | BP | negative regulation of phosphoprotein phosphatase activity | 0.0452674897119342 | 1 |
| GO:0033197 | BP | response to vitamin E | 0.0452674897119342 | 1 |
| GO:0034165 | BP | positive regulation of toll-like receptor 9 signaling pathway | 0.0452674897119342 | 1 |
| GO:0034371 | BP | chylomicron remodeling | 0.0452674897119342 | 1 |
| GO:0034486 | BP | vacuolar transmembrane transport | 0.0452674897119342 | 1 |
| GO:0034505 | BP | tooth mineralization | 0.0452674897119342 | 1 |
| GO:0035740 | BP | CD8-positive, alpha-beta T cell proliferation | 0.0452674897119342 | 1 |
| GO:0036150 | BP | phosphatidylserine acyl-chain remodeling | 0.0452674897119342 | 1 |
| GO:0036152 | BP | phosphatidylethanolamine acyl-chain remodeling | 0.0452674897119342 | 1 |
| GO:0038009 | BP | regulation of signal transduction by receptor internalization | 0.0452674897119342 | 1 |
| GO:0042853 | BP | L-alanine catabolic process | 0.0452674897119342 | 1 |
| GO:0045720 | BP | negative regulation of integrin biosynthetic process | 0.0452674897119342 | 1 |
| GO:0045906 | BP | negative regulation of vasoconstriction | 0.0452674897119342 | 1 |
| GO:0050923 | BP | regulation of negative chemotaxis | 0.0452674897119342 | 1 |
| GO:0055095 | BP | lipoprotein particle mediated signaling | 0.0452674897119342 | 1 |
| GO:0055096 | BP | low-density lipoprotein particle mediated signaling | 0.0452674897119342 | 1 |
| GO:0060177 | BP | regulation of angiotensin metabolic process | 0.0452674897119342 | 1 |
| GO:0060215 | BP | primitive hemopoiesis | 0.0452674897119342 | 1 |
| GO:0060267 | BP | positive regulation of respiratory burst | 0.0452674897119342 | 1 |
| GO:0060299 | BP | negative regulation of sarcomere organization | 0.0452674897119342 | 1 |
| GO:0060319 | BP | primitive erythrocyte differentiation | 0.0452674897119342 | 1 |
| GO:0060357 | BP | regulation of leucine import | 0.0452674897119342 | 1 |
| GO:0060358 | BP | negative regulation of leucine import | 0.0452674897119342 | 1 |
| GO:0060514 | BP | prostate induction | 0.0452674897119342 | 1 |

| | | | | |
|---|---|---|---|---|
| GO:0060515 | BP | prostate field specification | 0.0452674897119342 | 1 |
| GO:0060520 | BP | activation of prostate induction by androgen receptor signaling pathway | 0.0452674897119342 | 1 |
| GO:0060605 | BP | tube lumen cavitation | 0.0452674897119342 | 1 |
| GO:0060662 | BP | salivary gland cavitation | 0.0452674897119342 | 1 |
| GO:0060768 | BP | regulation of epithelial cell proliferation involved in prostate gland development | 0.0452674897119342 | 1 |
| GO:0060769 | BP | positive regulation of epithelial cell proliferation involved in prostate gland development | 0.0452674897119342 | 1 |
| GO:0060974 | BP | cell migration involved in heart formation | 0.0452674897119342 | 1 |
| GO:0061552 | BP | ganglion morphogenesis | 0.0452674897119342 | 1 |
| GO:0061554 | BP | ganglion formation | 0.0452674897119342 | 1 |
| GO:0061555 | BP | ganglion structural organization | 0.0452674897119342 | 1 |
| GO:0061556 | BP | trigeminal ganglion morphogenesis | 0.0452674897119342 | 1 |
| GO:0061559 | BP | cranial ganglion morphogenesis | 0.0452674897119342 | 1 |
| GO:0061560 | BP | cranial ganglion formation | 0.0452674897119342 | 1 |
| GO:0061561 | BP | trigeminal ganglion formation | 0.0452674897119342 | 1 |
| GO:0061562 | BP | cranial ganglion structural organization | 0.0452674897119342 | 1 |
| GO:0061563 | BP | trigeminal ganglion structural organization | 0.0452674897119342 | 1 |
| GO:0070165 | BP | positive regulation of adiponectin secretion | 0.0452674897119342 | 1 |
| GO:0070170 | BP | regulation of tooth mineralization | 0.0452674897119342 | 1 |
| GO:0070171 | BP | negative regulation of tooth mineralization | 0.0452674897119342 | 1 |
| GO:0070425 | BP | negative regulation of nucleotide-binding oligomerization domain containing signaling pathway | 0.0452674897119342 | 1 |
| GO:0070433 | BP | negative regulation of nucleotide-binding oligomerization domain containing 2 signaling pathway | 0.0452674897119342 | 1 |
| GO:0070445 | BP | regulation of oligodendrocyte progenitor proliferation | 0.0452674897119342 | 1 |
| GO:0070981 | BP | L-asparagine biosynthetic process | 0.0452674897119342 | 1 |
| GO:0070982 | BP | L-asparagine metabolic process | 0.0452674897119342 | 1 |
| GO:0071286 | BP | cellular response to magnesium ion | 0.0452674897119342 | 1 |
| GO:0071307 | BP | cellular response to vitamin K | 0.0452674897119342 | 1 |

| | | | | |
|---|---|---|---|---|
| GO:0071313 | BP | cellular response to caffeine | 0.0452674897119342 | 1 |
| GO:0071344 | BP | diphosphate metabolic process | 0.0452674897119342 | 1 |
| GO:0071613 | BP | granzyme B production | 0.0452674897119342 | 1 |
| GO:0071661 | BP | regulation of granzyme B production | 0.0452674897119342 | 1 |
| GO:0071663 | BP | positive regulation of granzyme B production | 0.0452674897119342 | 1 |
| GO:0071678 | BP | olfactory bulb axon guidance | 0.0452674897119342 | 1 |
| GO:0090245 | BP | axis elongation involved in somitogenesis | 0.0452674897119342 | 1 |
| GO:0090291 | BP | negative regulation of osteoclast proliferation | 0.0452674897119342 | 1 |
| GO:0090301 | BP | negative regulation of neural crest formation | 0.0452674897119342 | 1 |
| GO:0097156 | BP | fasciculation of motor neuron axon | 0.0452674897119342 | 1 |
| GO:0097360 | BP | chorionic trophoblast cell proliferation | 0.0452674897119342 | 1 |
| GO:0097534 | BP | lymphoid lineage cell migration | 0.0452674897119342 | 1 |
| GO:0097535 | BP | lymphoid lineage cell migration into thymus | 0.0452674897119342 | 1 |
| GO:0098713 | BP | leucine import across plasma membrane | 0.0452674897119342 | 1 |
| GO:0098909 | BP | regulation of cardiac muscle cell action potential involved in regulation of contraction | 0.0452674897119342 | 1 |
| GO:0098976 | BP | excitatory chemical synaptic transmission | 0.0452674897119342 | 1 |
| GO:0110026 | BP | regulation of DNA strand resection involved in replication fork processing | 0.0452674897119342 | 1 |
| GO:0140039 | BP | cell-cell adhesion in response to extracellular stimulus | 0.0452674897119342 | 1 |
| GO:0150094 | BP | amyloid-beta clearance by cellular catabolic process | 0.0452674897119342 | 1 |
| GO:0150102 | BP | negative regulation of monocyte activation | 0.0452674897119342 | 1 |
| GO:1900086 | BP | positive regulation of peptidyl-tyrosine autophosphorylation | 0.0452674897119342 | 1 |
| GO:1900154 | BP | regulation of bone trabecula formation | 0.0452674897119342 | 1 |
| GO:1900155 | BP | negative regulation of bone trabecula formation | 0.0452674897119342 | 1 |
| GO:1900826 | BP | negative regulation of membrane | 0.0452674897119342 | 1 |

| | | | | |
|---|---|---|---|---|
| | | depolarization during cardiac muscle cell action potential | | |
| GO:1901053 | BP | sarcosine catabolic process | 0.0452674897119342 | 1 |
| GO:1901382 | BP | regulation of chorionic trophoblast cell proliferation | 0.0452674897119342 | 1 |
| GO:1901383 | BP | negative regulation of chorionic trophoblast cell proliferation | 0.0452674897119342 | 1 |
| GO:1902202 | BP | regulation of hepatocyte growth factor receptor signaling pathway | 0.0452674897119342 | 1 |
| GO:1902204 | BP | positive regulation of hepatocyte growth factor receptor signaling pathway | 0.0452674897119342 | 1 |
| GO:1902724 | BP | positive regulation of skeletal muscle satellite cell proliferation | 0.0452674897119342 | 1 |
| GO:1902726 | BP | positive regulation of skeletal muscle satellite cell differentiation | 0.0452674897119342 | 1 |
| GO:1902897 | BP | regulation of postsynaptic density protein 95 clustering | 0.0452674897119342 | 1 |
| GO:1903238 | BP | positive regulation of leukocyte tethering or rolling | 0.0452674897119342 | 1 |
| GO:1903801 | BP | L-leucine import across plasma membrane | 0.0452674897119342 | 1 |
| GO:1903964 | BP | monounsaturated fatty acid metabolic process | 0.0452674897119342 | 1 |
| GO:1903966 | BP | monounsaturated fatty acid biosynthetic process | 0.0452674897119342 | 1 |
| GO:1904180 | BP | negative regulation of membrane depolarization | 0.0452674897119342 | 1 |
| GO:1904235 | BP | regulation of substrate-dependent cell migration, cell attachment to substrate | 0.0452674897119342 | 1 |
| GO:1904237 | BP | positive regulation of substrate-dependent cell migration, cell attachment to substrate | 0.0452674897119342 | 1 |
| GO:1904669 | BP | ATP export | 0.0452674897119342 | 1 |
| GO:1904878 | BP | negative regulation of calcium ion transmembrane transport via high voltage-gated calcium channel | 0.0452674897119342 | 1 |
| GO:1905135 | BP | biotin import across plasma membrane | 0.0452674897119342 | 1 |
| GO:1905516 | BP | positive regulation of fertilization | 0.0452674897119342 | 1 |
| GO:1905532 | BP | regulation of leucine import across plasma membrane | 0.0452674897119342 | 1 |
| GO:1905533 | BP | negative regulation of leucine | 0.0452674897119342 | 1 |

| | | | | |
|---|---|---|---|---|
| | | import across plasma membrane | | |
| GO:1990379 | BP | lipid transport across blood-brain barrier | 0.0452674897119342 | 1 |
| GO:1990700 | BP | nucleolar chromatin organization | 0.0452674897119342 | 1 |
| GO:2000035 | BP | regulation of stem cell division | 0.0452674897119342 | 1 |
| GO:2000096 | BP | positive regulation of Wnt signaling pathway, planar cell polarity pathway | 0.0452674897119342 | 1 |
| GO:2000118 | BP | regulation of sodium-dependent phosphate transport | 0.0452674897119342 | 1 |
| GO:2000120 | BP | positive regulation of sodium-dependent phosphate transport | 0.0452674897119342 | 1 |
| GO:2000564 | BP | regulation of CD8-positive, alpha-beta T cell proliferation | 0.0452674897119342 | 1 |
| GO:2000566 | BP | positive regulation of CD8-positive, alpha-beta T cell proliferation | 0.0452674897119342 | 1 |
| GO:2000807 | BP | regulation of synaptic vesicle clustering | 0.0452674897119342 | 1 |
| GO:2000809 | BP | positive regulation of synaptic vesicle clustering | 0.0452674897119342 | 1 |
| GO:2001187 | BP | positive regulation of CD8-positive, alpha-beta T cell activation | 0.0452674897119342 | 1 |
| GO:0060688 | BP | regulation of morphogenesis of a branching structure | 0.0453631700635991 | 3 |
| GO:0070227 | BP | lymphocyte apoptotic process | 0.0453631700635991 | 3 |
| GO:0003002 | BP | regionalization | 0.0464531398010402 | 11 |
| GO:0019722 | BP | calcium-mediated signaling | 0.0466238924324603 | 7 |
| GO:0050768 | BP | negative regulation of neurogenesis | 0.0474378125834014 | 5 |
| GO:0010002 | BP | cardioblast differentiation | 0.0477733806479257 | 2 |
| GO:0010592 | BP | positive regulation of lamellipodium assembly | 0.0477733806479257 | 2 |
| GO:0030852 | BP | regulation of granulocyte differentiation | 0.0477733806479257 | 2 |
| GO:0032743 | BP | positive regulation of interleukin-2 production | 0.0477733806479257 | 2 |
| GO:0033146 | BP | regulation of intracellular estrogen receptor signaling pathway | 0.0477733806479257 | 2 |
| GO:0033273 | BP | response to vitamin | 0.0477733806479257 | 2 |
| GO:0035437 | BP | maintenance of protein localization in endoplasmic reticulum | 0.0477733806479257 | 2 |
| GO:0036037 | BP | CD8-positive, alpha-beta T cell activation | 0.0477733806479257 | 2 |
| GO:0046949 | BP | fatty-acyl-CoA biosynthetic process | 0.0477733806479257 | 2 |

| | | | | |
|---|---|---|---|---|
| GO:0050901 | BP | leukocyte tethering or rolling | 0.0477733806479257 | 2 |
| GO:0060973 | BP | cell migration involved in heart development | 0.0477733806479257 | 2 |
| GO:0140354 | BP | lipid import into cell | 0.0477733806479257 | 2 |
| GO:1901224 | BP | positive regulation of NIK/NF-kappaB signaling | 0.0477733806479257 | 2 |
| GO:1903670 | BP | regulation of sprouting angiogenesis | 0.0477733806479257 | 2 |
| GO:0060429 | BP | epithelium development | 0.0479060327450341 | 21 |
| GO:1905114 | BP | cell surface receptor signaling pathway involved in cell-cell signaling | 0.0480180115767829 | 16 |
| GO:0019932 | BP | second-messenger-mediated signaling | 0.0482448381402124 | 9 |
| GO:0060828 | BP | regulation of canonical Wnt signaling pathway | 0.0482448381402124 | 9 |
| GO:0001568 | BP | blood vessel development | 0.0490535531420349 | 15 |
| GO:1902533 | BP | positive regulation of intracellular signal transduction | 0.049254234759308 | 21 |
| GO:0006766 | BP | vitamin metabolic process | 0.0496645441281126 | 4 |

Table S6. Enriched KEGG terms for DEGs identified between the LX and NX comparison group.

| KEGG ID | Term | Pvalue | Number |
|---|---|---|---|
| map04145 | Phagosome | 3.23186759366966e-07 | 28 |
| map03320 | PPAR signaling pathway | 3.34479040131212e-06 | 13 |
| map04514 | Cell adhesion molecules | 3.57476814254615e-05 | 25 |
| map00260 | Glycine, serine and threonine metabolism | 0.0002583050133685 | 8 |
| map01212 | Fatty acid metabolism | 0.0003491809913313 | 10 |
| map04672 | Intestinal immune network for IgA production | 0.0033682040616585 | 11 |
| map05168 | Herpes simplex virus 1 infection | 0.0035362841110097 | 23 |
| map00270 | Cysteine and methionine metabolism | 0.0041502181109275 | 7 |
| map00071 | Fatty acid degradation | 0.0047331307320962 | 6 |
| map04820 | Cytoskeleton in muscle cells | 0.0061589013538984 | 21 |
| map04218 | Cellular senescence | 0.0072461703935644 | 15 |
| map04148 | Efferocytosis | 0.0092050363816084 | 14 |
| map04920 | Adipocytokine signaling pathway | 0.0096455011296741 | 8 |
| map01040 | Biosynthesis of unsaturated fatty acids | 0.010204850395053 | 5 |
| map04512 | ECM-receptor interaction | 0.0128107362055444 | 10 |
| map00250 | Alanine, aspartate and glutamate metabolism | 0.0148477618349146 | 5 |
| map01230 | Biosynthesis of amino acids | 0.0221262192400366 | 7 |
| map00062 | Fatty acid elongation | 0.0321264855253437 | 4 |
| map00650 | Butanoate metabolism | 0.0321264855253437 | 4 |
| map04020 | Calcium signaling pathway | 0.0404847779880873 | 21 |
| map04060 | Cytokine-cytokine receptor interaction | 0.0435511172937152 | 16 |
| map04510 | Focal adhesion | 0.0494640964860136 | 15 |
| map00770 | Pantothenate and CoA biosynthesis | 0.0498488251731883 | 3 |
| map00910 | Nitrogen metabolism | 0.0498488251731883 | 3 |
